# Synthesis of the ABC Ring of Calyciphylline A-Type Alkaloids by a Stereocontrolled Aldol Cyclization: Formal Synthesis of (±)-Himalensine A

**DOI:** 10.1021/acs.joc.2c01171

**Published:** 2022-07-21

**Authors:** Clàudia Marquès, Faïza Diaba, Enrique Gómez-Bengoa, Josep Bonjoch

**Affiliations:** † Laboratori de Química Orgànica, Facultat de Farmàcia, IBUB, Universitat de Barcelona, Av. Joan XXIII 27-31, 08028-Barcelona, Spain; ‡ Departamento de Química Orgánica I, Universidad del País Vasco, Manuel Lardizábal 3, 20018 San Sebastián, Spain

## Abstract

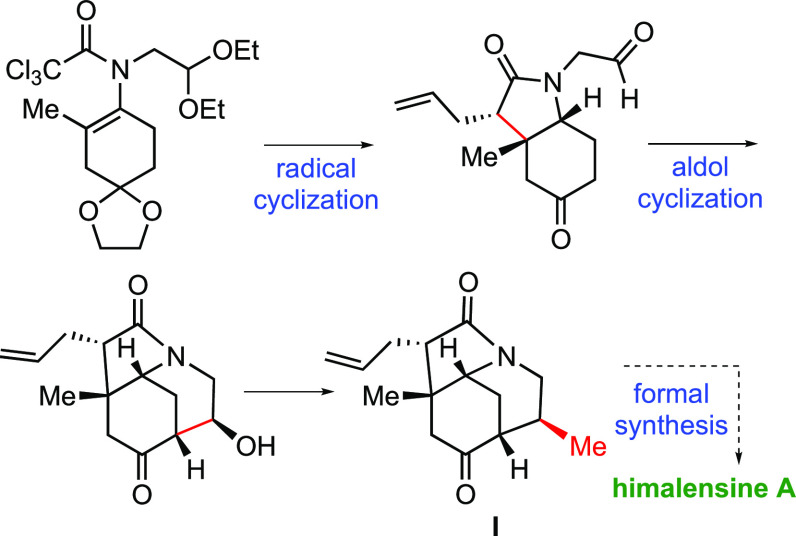

A synthetic approach to a functionalized ABC-tricyclic framework of calyciphilline A-type alkaloids, a building block toward this class of alkaloids, is reported. The key synthetic steps involve a radical cyclization to form the hydroindole system and piperidine ring closure through a stereocontrolled aldol cyclization. The resulting alcohol allows the methyl group to be installed in the bowl-shaped azatricyclic structure; this crucial reaction takes place with configuration retention. The synthesis of azatricyclic compound **I** constitutes a formal synthesis of himalensine A.

Calyciphylline A-type *Daphniphyllum* alkaloids^1^ feature a backbone of four rings [6–6–5–7] including a bridged morphan subunit with one or two additional five-membered rings fused at the seven-membered ring (Figure 1).

**Figure 1:**
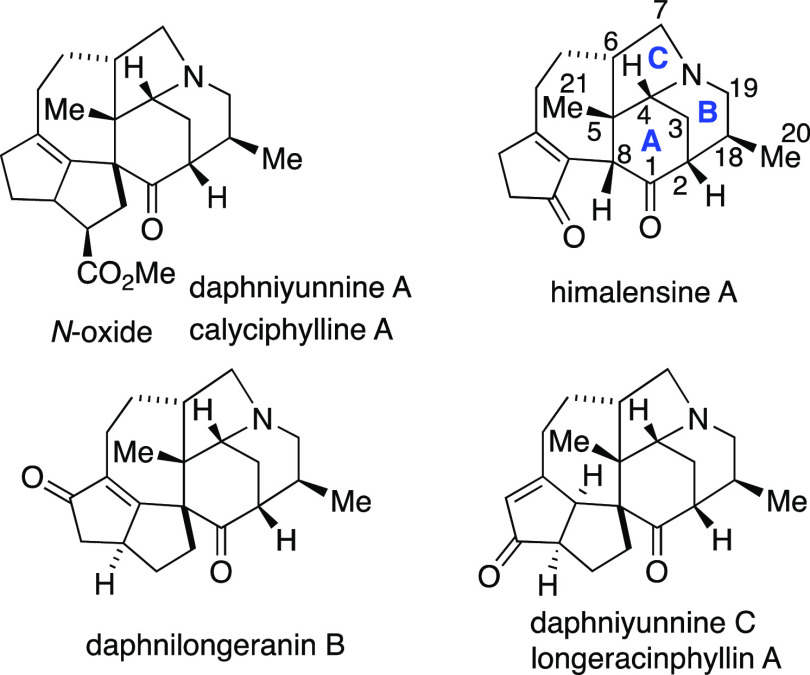
Representative *Daphniphyllum* alkaloids embodying the 1,6-ethanoindole ring (ABC framework; biosynthetic numbering).

Compounds embodying the compact azatricyclic ABC ring system, with an all-carbon quaternary center at C-5 and the methyl group installed at C-18, are valuable building blocks for the synthesis of calyciphylline-A type alkaloids as well as some other *Daphniphyllum* alkaloids. Synthetic precedents for compounds containing this 1,6-ethanooctahydroindole scaffold with a suitable substitution pattern and functionalization for the preparation of calyciphylline-A type alkaloids are summarized in Scheme 1. The different synthetic methodologies used for the last ring closure are as follows. (a) Pd-catalyzed alkenylation of ketones, a procedure developed in our research group (Scheme 1a),^2^ enabled the first synthesis of the ABC fragment of the target alkaloids^3^ and was subsequently used by Gao to achieve himalensine A,^4^ by Liang for the synthesis of the ABCE rings of daphenylline,^5^ and by Xue and Qin in studies devoted to a substructure of 21-deoxymacropodumine D.^6^ A variation was reported by Tang^7^ in which a Pd-catalyzed oxidative alkenylation using Pd(OAc)_2_ and Yb(OTf)_3_ was carried out from an alkene-tethered β-keto ester. (b) A radical tandem process was developed by Stockdill (Scheme 1b)^8^ in which the piperidine B ring was closed by construction of the morphan nucleus from an aminyl radical and trapping of the carbon-centered radical formed by an alkyne. (c) Intramolecular Michael addition from a β-amido ester to an enone was used intensively by Li (Scheme 1c)^9^ en route to the total syntheses of several *Daphniphyllum* alkaloids. Also, our group developed a process using a sulfone as a source of a carbanion to generate the pyrrolidine C ring with concomitant formation of the stereogenic quaternary carbon.^10,11^

**Scheme 1:**
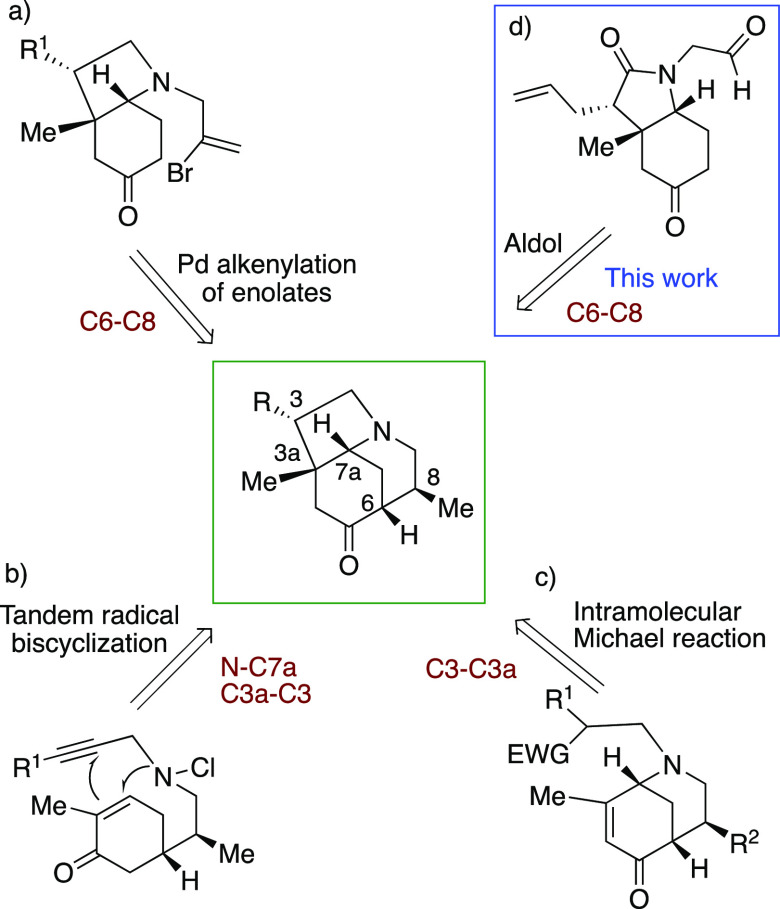
Previous Approaches to the Tricyclic ABC Core of the Calyciphylline A-Type Alkaloids^,^ Functionalized 3a,8-dimethyl-1,6-ethanohydroindoles (ABC ring of calyciphylline-A alkaloids). Systematic numbering is used in the results, discussion, and Experimental Section.

Herein, a new approach to the ABC ring is described, which differs from the aforementioned synthetic strategies in that an aldol cyclization is used to achieve the targeted azatricyclic ring (Scheme 1d). The synthetic route to the ABC ring of calyciphylline A-type alkaloids embodying suitable funcionalization and substituents toward the synthesis of *Daphniphyllum* alkaloids is depicted in Scheme 2. Control of the stereochemistry at C8 through a substrate-directable methylation process would prove to be crucial in this synthetic proposal (Scheme 3).

**Scheme 2:**
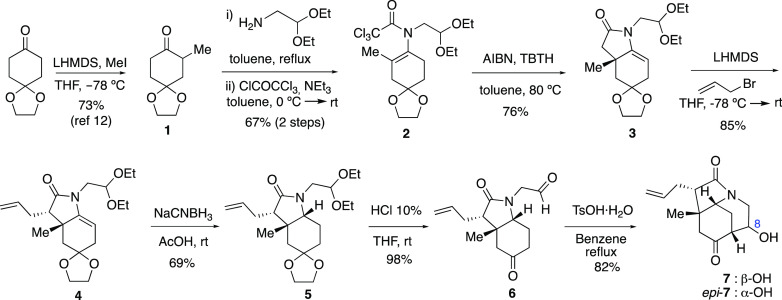
Synthesis of the ABC Azatricyclic Ring of Calyciphylline A-Type Alkaloids

**Scheme 3:**
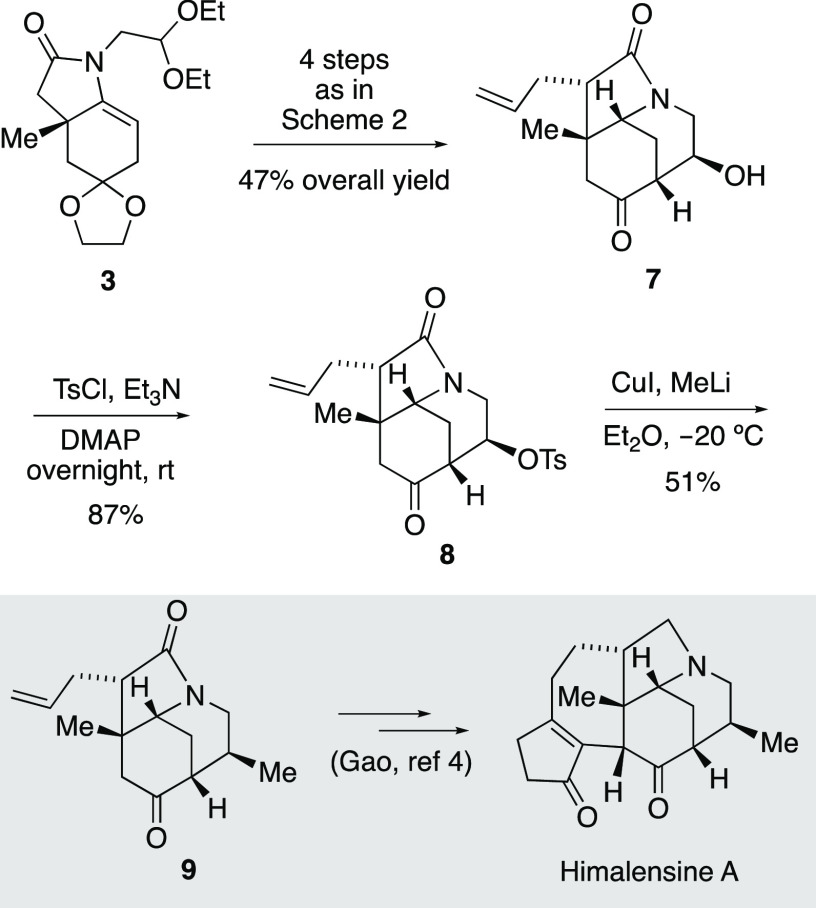
Formal Synthesis of Himalensine A

Commencing from the easily available ketone **1**,^12^ the cyclization precursor **2** was accessed via imine formation followed by trichloroacetylation using the general protocol to prepare related trichloroenamides,^13^ which allowed the gram-scale synthesis of the required polyfunctionalized starting material. Radical cyclization of trichloroacetamide **2**(14) furnished enelactam **3** (76%), which was diastereoselectively allylated to provide **4** in which the enelactam function was chemoselectively reduced to afford octahydroindole **5**. It is worth mentioning that both enelactams **3** and **4** were sensitive to the hydration process, being prone to undergo partial evolution to their corresponding hemiaminal (i.e., the 7a-hydroxylated derivative, Chart 1) whether in an open-air atmosphere or during a chromatographic process (SiO_2_). However, this transformation is reversible, as enelactams **3** and **4**, if formed, can be recovered by dehydration under acidic conditions (see Chart 1 and the Experimental Section). A double acetal deprotection from **5** provided the keto aldehyde required to carry out the piperidine ring closure through an aldol process. The aldol cyclization furnished a separable diastereomeric mixture (9:1) with 90% overall yield. At the time, the stereochemical course of the aldol process was not evident from the NMR data of the isolated azatricyclic ketols **7** and *ep*i-**7**.

**Chart 1:**
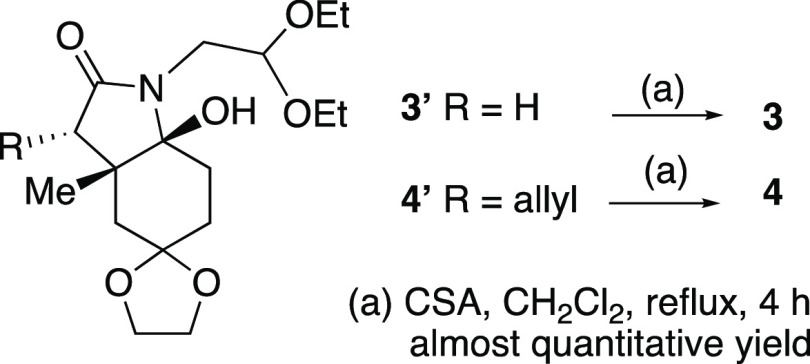


At this point, for the sake of efficiency, a modified protocol for rapid access to compound **7** was evaluated. Thus, a chromatography-free, four-step sequence for the transformation of enelactam **3** to azatricyclo **7** was tested. Bypassing the purification step of intermediate compounds avoided the expense of chromatography, but the overall yield was lower (30% [see SI] versus 47%), and the process was only moderately less time consuming than the one reported in Scheme 2.

At this stage, it was not possible to establish the configuration of the stereogenically formed C-8 in aldol **7** as the well-precedented nonchair conformation in the piperidine ring of this type of azatricyclic compounds makes it difficult to correlate the coupling constants of protons in the ^1^H NMR spectrum with their spatial arrangement. The configuration could not be conclusively determined by the NOESY spectrum of **7**.

We then investigated the origin of the stereoselectivity by means of DFT calculations.^15^ Although the two alcohol epimers at C-8 were isolated in a 9:1 ratio, the calculations showed that the stability difference between both isomers (**7** and *epi*-**7**) is negligible, less than 0.1 kcal/mol (Figure 2), which strongly indicates the absence of equilibrium between **7** and *epi*-**7** in the reaction conditions.^16^ We thus hypothesized that the stereochemical course of the aldol reaction promoted by the Brønsted acid is not the result of thermodynamic control, as previously thought. Instead, it can be rationalized by the kinetic preference in the approach of the enol to the aldehyde during the transition states of the reaction. To confirm the kinetic control, we analyzed the structures of the model attack of the ketone enol to the aldehyde in **6** in the presence of a *p*-TsOH molecule. Our model shows that the aldehyde group preferentially adopts a disposition that favors an attack from the *Si* face of the carbonyl group (**TS1**, Figure 2), which is 2.3 kcal/mol lower in energy than the *Re* approach (*epi*-**TS1**), disfavored by steric and electronic factors. Activation of the aldehyde presumably results in the dipole minimized orientation^17^ of the dicarbonyl unit, which can then be attacked by the tethered enol via the lower energy intermediate, thus providing **7**. The reaction is hence exothermic by more than 12 kcal/mol, corroborating the nonreversibility of the process in acidic conditions.

**Figure 2:**
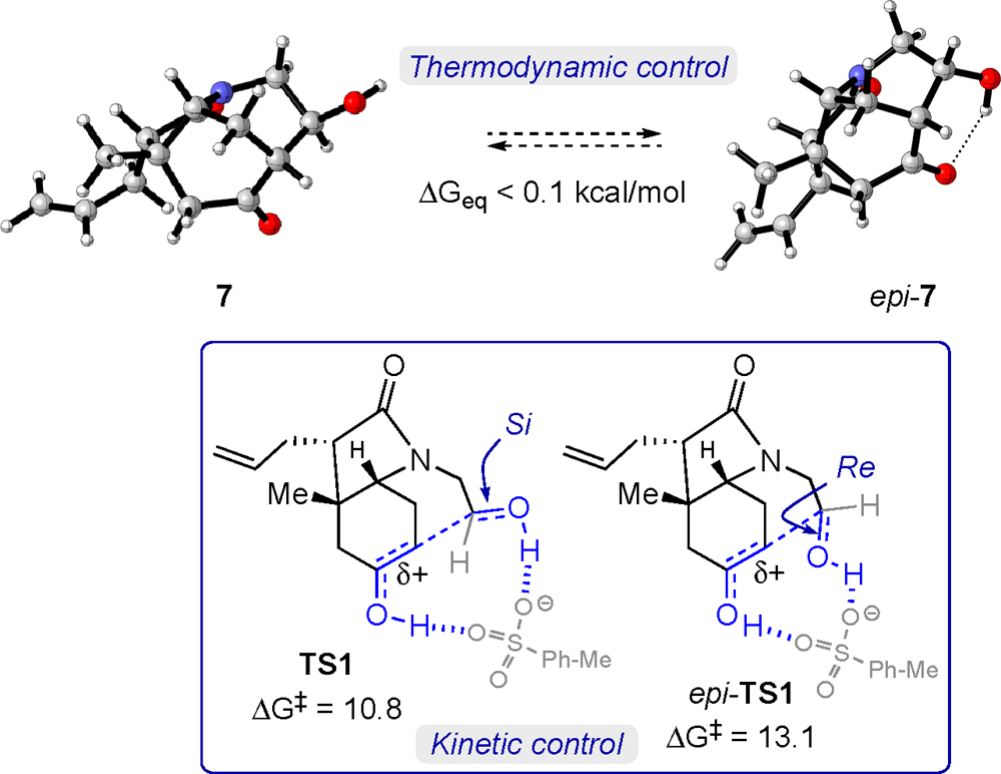
DFT calculations of the aldol cyclization of **6**: *si*-face attack (**TS1**) and *re*-face attack (*epi*-**TS1**) at the M06-2X/6-311++G(d,p) (IEFPCM, benzene) level. Energies are given in kcal/mol.

The DFT-based proposal for the relative configuration of cyclic alcohol **7** was confirmed after its transformation to tosylate **8**, whose configuration and hence that of its precursor (i.e., ketol **7**) at C-8 was ascertained by X-ray crystallography (Figure 3). The X-ray structure of compound **8** proved that the tosylate substituent at C-8 is cis to the bridged hydrogen atom at C-6 and occupies a pseudoequatorial position in the crystal that reflects the boat form of the morphan substructure and ensures the relative configuration of ketol **7**. Interestingly, it should be noted that in the related aldol process leading to a bicyclic morphan compound,^18^ in which the bicyclic system allows a chair–chair conformation, a reverse diastereoselectivity was observed for the keto-tethered aldehyde cyclization using the same reaction conditions.^19^

**Figure 3:**
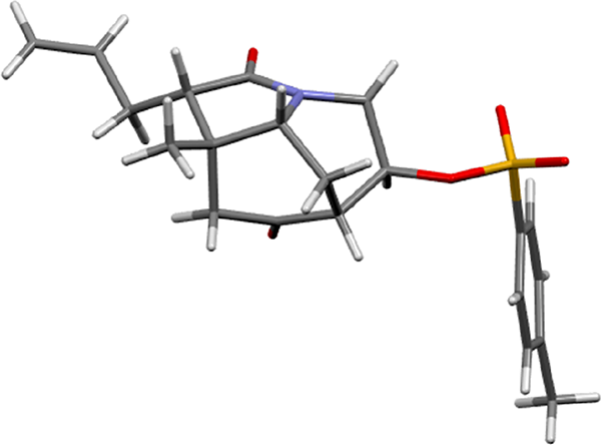
X-ray of tosylate **8**.

The last step in the synthesis of the targeted azatricycle **9** was the installation of the methyl group at C-8 with the correct relative configuration. Gratifyingly, the Me_2_CuLi formed in situ by treatment of MeLi with CuI led to a chemo- and diastereoselective reaction in which the tosylate group was substituted by a methyl with retention of configuration.^20^ In methodological studies, the possibility of configuration retention is sometimes observed, and a speculative mechanism involving radical species has been proposed.^21^ In natural product synthesis, there are few examples of a tosylate/sulfonate group being replaced by an alkyl or aryl group with stereochemical retention, and the displacement mechanism usually involves the participation of a neighboring group.^22^ Alternatively,^23^ if tosylate **8** undergoes a β-elimination involving a bridgehead enone formation,^24^ a conjugate addition of Me_2_CuLi upon the enone would be the origin of the diastereoselective formation of compound **9**.

The stereochemical assignment was unequivocally established considering that the spectroscopic data of the resulting compound **9** were identical in all respects to those reported by Gao for this compound structure^25^ en route to his recent total synthesis of himalensine A. Thus, the stereoselectively formed azatricycle **9** showed the same relative configuration in its five stereogenic centers as in all calyciphylline A-type alkaloids embodying this azatricyclic scaffold.

In summary, concise access to 1,6-ethanoperhydroindole azatricycle **9** (i.e., **I**) has been accomplished, thereby providing a formal synthesis of himalensine A^4,26^ The whole process requires 10 reaction steps and provides compound **9** in an overall yield of 8%. The synthesis, based on an intramolecular aldol process, is a new approach to the ABC ring system for the calyciphylline A-type subset of *Daphniphyllum* alkaloids.

## Experimental Section

### General

All reactions were carried out under an argon atmosphere with dry, freshly distilled solvents under anhydrous conditions. All product mixtures were analyzed by thin-layer chromatography using TLC silica gel plates with a fluorescent indicator (λ = 254 nm). Analytical thin-layer chromatography was performed on SiO_2_ (Merck Silica Gel 60 F_254_), and the spots were located by a UV light and/or a 1% KMnO_4_ aqueous solution or hexachloroplatinate reagent. Chromatography refers to flash chromatography and was carried out on SiO_2_ (Carlo Erba 60A, 35–70 μ) or on Al_2_O_3_ (neutral aluminum oxide, 0.063–0.2 mm). Drying of the organic extracts during the reaction workup was performed over anhydrous Na_2_SO_4_. Chemical shifts of the ^1^H and ^13^C NMR spectra are reported in ppm downfield (δ) from Me_4_Si (δ 0.00) and CDCl_3_ (δ = 77.00), respectively. All NMR data assignments are supported by gCOSY and gHSQC experiments. HRMS were obtained with an LC/MSD-TOF spectrometer (Agilent Technologies, ESI-MS).

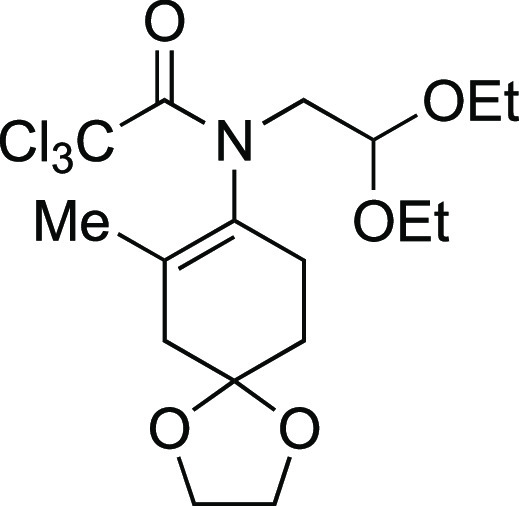



### 2,2,2-Trichloro-*N*-(2,2-diethoxyethyl)-*N*-(2-methyl-4-oxocyclohex-1-enyl)acetamide Ethylene Acetal (**2**)

2-Methylcyclohexane-1,4-dione monoethylene acetal (**1**,^12^ 3.20 g, 18.7 mmol) and aminoacetaldehyde diethyl acetal (2.7 mL, 18.7 mmol) were dissolved in toluene (45 mL) and placed under Dean–Stark conditions for 4 h. A solution of trichloroacetyl chloride (2.3 mL, 20.6 mmol, 1.1 equiv) in toluene (20 mL) was cooled to 0 °C, and the above solution containing the imine was added dropwise. The reaction was stirred at room temperature for 1 h and cooled to 0 °C, and a solution of NEt_3_ (7.8 mL, 56.2 mmol) in toluene (45 mL) was added. After being stirred for 2 h at room temperature, an aqueous Na_2_CO_3_-saturated solution (70 mL) was added, and the mixture was stirred for 1 h and extracted with Et_2_O (3 × 50 mL). The organics were combined, dried, concentrated, and purified by chromatography (hexane:EtOAc, 9.5:0.5 → 1:1) to afford compound **2** (5.07 g, 67%) as a colorless oil. IR (neat) 2975, 2932, 1716, 1672, 1375, 1127, 1060, 822 cm^–1^; ^1^H NMR (400 MHz, CDCl_3_) δ 4.88 (dd, *J* = 6.4, 3.2 Hz, 1H, CH), 4.00 (dd, *J* = 13.8, 2.0 Hz, 1H, NCH_2_), 4.00–3.94 (m, 4H, OCH_2_), 3.82–3.68 and 3.65–3.49 (2 m, 2H each, OCH_2_CH_3_), 3.11 (dd, *J* = 13.8, 6.6 Hz, 1H, NCH_2_), 2.68 (m, 1H, H-5), 2.51 (m, 1H, H-5), 2.31 and 2.20 (2 d, *J* = 18.0 Hz, 1H each, H-3), 1.80 (m, 2H, H-6), 1.62 (s, 3H, Me), 1.21 (t, *J* = 7.0 Hz, 6H, CH_3_); ^13^C{^1^H} NMR (101 MHz, CDCl_3_) δ 161.4 (CO), 133.4 (C-1), 131.3 (C-2), 107.1 (C-4), 99.2 (CH), 64.5 and 64.4 (OCH_2_), 63.9 and 63.2 (OCH_2_CH_3_), 56.9 (NCH_2_), 41.0 (C-3), 31.4 (C-5), 28.4 (C-6), 20.2 (Me), 15.3 and 15.2 (CH_3_); HRMS (ESI-TOF) *m*/*z* [M + H]^+^ calcd for C_17_H_27_Cl_3_NO_5_ 430.0955, found 430.0963.

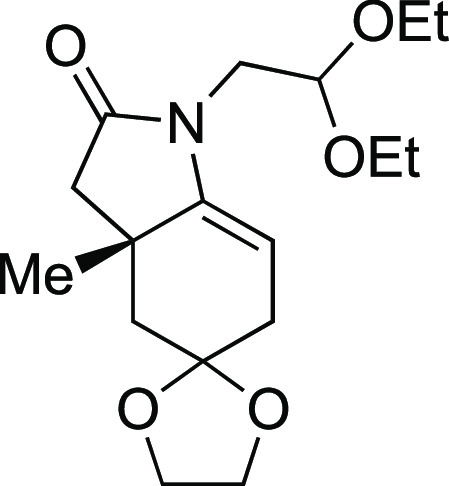



### 1-(2,2-Diethoxyehtyl)-3a-methyl-1,3a,4,6-tetrahydro-2*H*-indole-2,5(3*H*)-dione Monoethylene Acetal (**3**)

A solution of **2** (7.77 g, 17.9 mmol) in benzene (200 mL) was heated to 80 °C with a heating block, and a solution of AIBN (1.46 g, 8.95 mmol) and Bu_3_SnH (17 mL, 62.7 mmol) in benzene (20 mL) was added over 3 h using a syringe pump. The reaction was stirred for an additional hour at this temperature, cooled, and concentrated. The residue was purified by chromatography (hexane:EtOAc, 1:0 → 4:1) to give **3** as a colorless oil (4.14 g, 71%). In some runs, a small quantity of **3′** was isolated (less than 5%) as a colorless oil.^27^

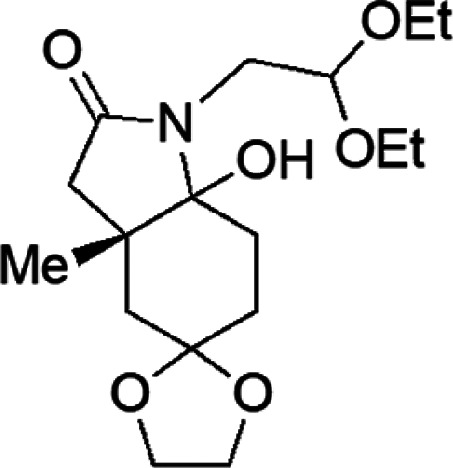



Compound **3**: IR (neat) 2975, 2885, 1726, 1686, 1118, 1067 cm^–1^; ^1^H NMR (400 MHz, CDCl_3_) δ 4.90 (t, *J* = 4.0 Hz, 1H, H-7), 4.72 (dd, *J* = 6.2, 5.0 Hz,1H, CH), 4.03–4.00 and 3.95–3.87 (2 m, 2H each, OCH_2_), 3.78 (dd, *J* = 14.0, 6.4 Hz, 1H, NCH_2_), 3.74–3.66 and 3.55–3.47 (2 m, 2H each, OCH_2_CH_3_), 3.36 (dd, *J* = 14.0, 5.0 Hz, 1H, NCH_2_), 2.50 (br t, *J* = 3.7 Hz, 2H, H-6), 2.31 and 2.27 (2d, *J* = 16.4 Hz, 1H each, H-3), 2.04 and 1.85 (2 d, *J* = 13.4 Hz, 1H each, H-4), 1.30 (s, 3H, Me), 1.19 and 1.17 (2 t, *J* = 7.0 Hz, 3H each, CH_3_); ^13^C{^1^H} NMR (101 MHz, CDCl_3_) δ 174.0 (C-2), 145.8 (C-7a), 108.6 (C-5), 98.8 (CH), 94.5 (C-7), 64.4 and 63.7 (OCH_2_), 62.5 and 62.1 (OCH_2_CH_3_), 46.7 (C-3), 43.3 (C-4), 42.4 (NCH_2_), 37.5 (C-3a), 35.1 (C-6), 25.6 (Me), 15.2 and 15.2 (CH_3_); HRMS (ESI-TOF) *m*/*z* [M + H]^+^ calcd for C_17_H_28_NO_5_ 326.1967, found 326.1975.

Compound **3′**: IR (neat) 3405, 2973, 2937, 1705, 1424, 1070 cm^–1^; ^1^H NMR (400 MHz, CDCl_3_) δ 4.63 (dd, *J* = 7.7, 2.8 Hz, 1H, CH), 3.98–3.85 (m, 5H, OCH_2_, NCH_2_), 3.82 (dq, *J* = 9.3, 7.1 Hz, 1H, OCH_2_), 3.76–3.63 (m, 2H, OCH_2_), 3.51 (dq, *J* = 9.3, 7.1 Hz, 1H, OCH_2_), 2.92 (dd, *J* = 14.6, 7.7 Hz, 1H, NCH_2_), 2.39 and 2.14 (2d, *J* = 16.0 Hz, 1H each, H-3), 2.10–1.96 (m, 2H, H-7), 1.65 (m, 1H, H-6), 1.66 and 1.55 (2d, *J* = 14.2 Hz, 1H each, H-4), 1.38 (td, *J* = 12.8, 4.0 Hz, 1H, 1H-6), 1.25 (t, *J* = 7.1 Hz, 3H, CH_3_), 1.24 (s, 3H, Me), 1.23 (t, *J* = 7.2 Hz, 3H, CH_3_). ^13^C{^1^H} NMR (101 MHz, CDCl_3_) δ 175.8 (C-2), 107.6 (C-5), 100.0 (CH), 90.3 (C-7a), 64.4 (OCH_2_), 64.2 (OCH_2_CH_3_), 64.0 (OCH_2_), 63.7 (OCH_2_CH_3_), 44.7 (C-3), 43.3 (C-4), 42.1 (NCH_2_ and C-3a), 30.8 (C-6), 29.1 (C-7), 20.4 (Me), 15.4 and 14.8 (CH_3_). HRMS (ESI-TOF) *m*/*z* [M + H – H_2_O]^+^ calcd for C_17_H_28_NO_5_ 326.1967, found 326.1965.

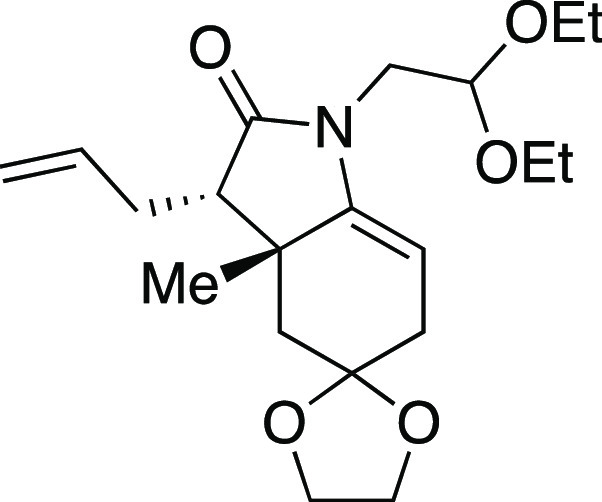



### 3-Allyl-1-(2,2-diethoxyehtyl)-3a-methyl-1,3a,4,6-tetrahydro-2*H*-indole-2,5(3*H*)-dione Monoethylene Acetal (**4**)

A solution of lactam **3** (524 mg, 1.6 mmol) was cooled to −78 °C, and a solution of LHMDS in THF (1 M, 2.08 mL) was added dropwise. After being stirred for 30 min at −78 °C, allyl bromide (0.29 mL, 3.2 mmol) was added. The reaction was left to reach room temperature over 2 h, quenched with a NH_4_Cl-saturated solution (50 mL), and extracted with Et_2_O (3 × 20 mL). The organics were dried and purified by chromatography (Al_2_O_3_, hexane:EtOAc 9:1) to give **4** (674 mg, 85%) as a colorless oil: IR (neat) 2975, 1724, 1684, 1406, 1340, 1128, 1069 cm^–1^; ^1^H NMR (400 MHz, CDCl_3_) δ 5.87–5.75 (m, 1H, =CH), 5.06 (d, *J* = 16.4 Hz, 1H, =CH_2_ H-*trans*), 5.03 (d, *J* = 9.2 Hz, 1H, =CH_2_ H-*cis*), 4.93 (t, *J* = 3.6 Hz, 1H, H-7), 4.71 (dd, *J* = 6.4, 4.8 Hz, 1H, CH), 4.04–4.01 and 3.99–3.87 (2 m, 2H each, OCH_2_), 3.76–3.62 (m, 3H, OCH_2_CH_3_ and NCH_2_), 3.58–3.48 (m, 2H, OCH_2_CH_3_), 3.44 (dd, *J* = 14.0, 4.8 Hz, 1H, NCH_2_), 2.47 (d, *J* = 3.8 Hz, 2H, H-6), 2.32–2.14 (m, 3H, 3-CH_2_ and H-3), 2.01 and 1.74 (2 d, *J* = 13.4 Hz, 1H each, H-4), 1.32 (s, 3H, Me), 1.18 (2 t, *J* = 7.0 Hz, 3H each, CH_3_); ^13^C{^1^H} NMR (101 MHz, CDCl_3_) δ 176.5 (C-2), 144.5 (C-7a), 135.3 (=CH), 116.7 (=CH_2_), 108.7 (C-5), 98.8 (CH), 95.4 (C-7), 64.5 and 63.6 (OCH_2_), 62.5 and 62.2 (OCH_2_CH_3_), 54.0 (C-3), 42.6 (NCH_2_), 40.6 (C-3a), 37.7 (C-4), 35.0 (C-6), 32.8 (3-CH_2_), 27.8 (Me), 15.2 (CH_3_). HRMS (ESI-TOF) *m*/*z* [M + H]^+^ calcd for C_20_H_32_NO_5_ 366.2280, found 366.2289.

When the crude reaction mixture was chromatographed using SiO_2_ (hexane:EtOAc, 9:1) the hydrated compound **4′** was isolated in 69% yield.^27^

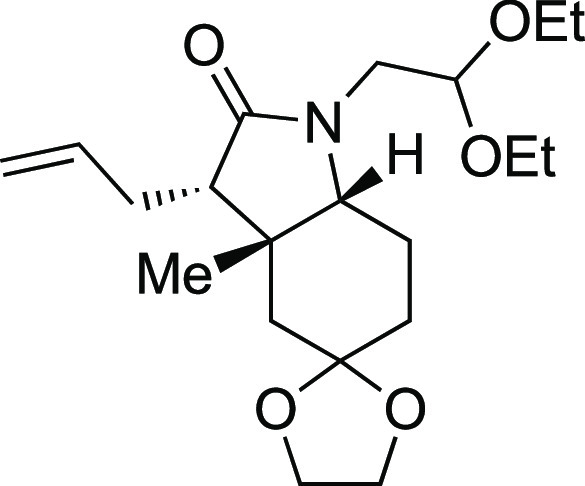



### (3*RS*,3a*SR*,7a*SR*)-3-Allyl-1-(2,2-diethoxyehtyl)-3a-methylhexahydro-2*H*-indole-2,5(3*H*)-dione Monoethylene Acetal (**5**)

To a solution of lactam **4** (472 mg, 1.29 mmol) in AcOH (2.2 mL) was added NaCNBH_3_ (162 mg, 2.58 mmol) portionwise, and the reaction mixture was stirred at room temperature for 2.5 h. MeOH was added, and after an additional 15 min of stirring, the mixture was concentrated; the residue was taken up in CH_2_Cl_2_, quenched with 15% NaOH, and extracted with CH_2_Cl_2_ (4 × 20 mL). The combined organics were dried, concentrated, and purified by chromatography (hexane:EtOAc, 1:0 → 1:1) to provide compound **5** (328 mg, 69%) as a colorless oil. IR (neat) 2973, 2880, 1692, 1126, 1092, 1063 cm^–1^; ^1^H NMR (400 MHz, CDCl_3_) δ 6.01–5.90 (m, 1H, =CH), 5.11 (ddt, *J* = 17.0, 0.8, 1.6 Hz, 1H, =CH_2_ H-*trans*), 5.01 (ddt, *J* = 10.4, 1.6, 1.6, 1H, =CH_2_ H-*cis*), 4.58 (dd, *J* = 6.6, 4.4 Hz, 1H, CH), 3.96–3.85 (m, 5H, NCH_2_, OCH_2_) 3.72, 3.69, 3.56, and 3.48 (4 dq, *J* = 9.4, 7.0 Hz, 1H each), 3.30 (t, *J* = 3.0 Hz, 1H, H-7a), 2.85 (dd, *J* = 14.2, 6.4 Hz, 1H, NCH_2_), 2.60–2.50 (m, 1H, 3-CH_2_), 2.20–2.04 (m, 3H, 3-CH_2_, 1H-7, H-3), 1.89 (ddt, *J* = 15.4, 14.4, 3.6 Hz, 1H, H-7ax), 1.51 (dq, *J* = 13.4, 3.2 Hz, 1H, H-6), 1.48 and 1.39 (2 d, *J* = 14.8 Hz, 1H each, H-4), 1.36 (dd, *J* = 13.4, 3.4 Hz, 1H, H-6), 1.27 (s, 3H, Me), 1.20 and 1.18 (2t, *J* = 7.0 Hz, 3H each, CH_3_); ^13^C{^1^H} NMR (101 MHz, CDCl_3_) δ 176.9 (C-2), 137.6 (=CH), 115.7 (=CH_2_), 108.2 (C-5), 100.7 (CH), 64.5 and 63.7 (OCH_2_), 63.5 and 62.8 (OCH_2_CH_3_), 60.8 (C-7a), 54.9 (C-3), 42.5 (NCH_2_), 42.0 (C-3a), 36.5 (C-4), 28.8 (3-CH_2_), 28.4 (C-6), 22.9 (Me), 19.8 (C-7), 15.4 and 15.3 (CH_3_); HRMS (ESI-TOF) *m*/*z* [M + H]^+^ calcd for C_20_H_34_NO_5_ 368.2437, found 368.2447.

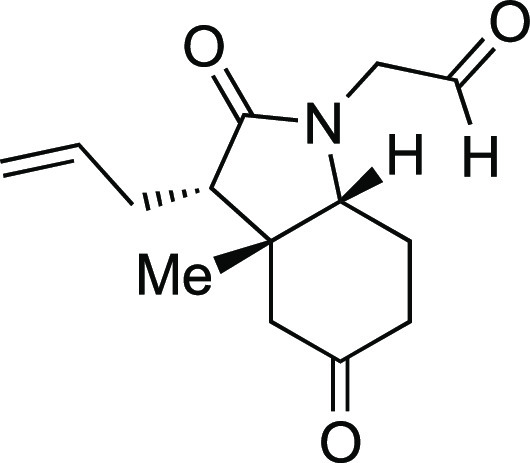



### (3*RS*,3a*SR*,7a*SR*)-3-Allyl-1-(2-oxoethyl)tetrahydro-1*H*-indole-2,5(3*H*,6*H*)dione (**6**)

A solution of **5** (259 mg, 0.7 mmol) in 10% HCl:THF (1:4, 14 mL) was stirred overnight at room temperature. The mixture was diluted with water (10 mL) and extracted with CH_2_Cl_2_ (3 × 15 mL). The combined organic extracts were dried, concentrated, and purified by chromatography (hexane:EtOAc, 3:1) to provide aldehyde **6** as a colorless oil (172 mg, 98%). IR (neat) 3404, 2934, 1716, 1686, 1430, 1059 cm^–1^; ^1^H NMR (400 MHz, CDCl_3_) δ 9.65 (s, 1H, CHO), 5.90 (dddd, J = 17.1, 10.1, 8.7, 5.2 Hz, 1H, =CH), 5.14 (d, *J* = 17.1 Hz, 1H, =CH_2_ H-*trans*), 5.07 (d, *J* = 10 Hz, 1H, =CH_2_ H-*cis*), 4.56 and 3.94 (2 d, *J* = 18.9 Hz, 1H each, NCH_2_), 3.57 (t, *J* = 3.6 Hz, 1H, H-7a), 2.66 (dm, *J* = 15.0 Hz, 1H, 3-CH_2_), 2.50–2.44 (m, 2H, H-3, H-4), 2.35–2.25 and 2.22–2.20 (m, 1H, H-6), 2.19 (dtd, *J* = 14.6, 4.3, 1.8 Hz, 1H, H-6), 2.17–2.05 (m, 4H, 3-CH_2_, H-4, H-7), 1.24 (s, 3H, Me); ^13^C{^1^H} NMR (101 MHz, CDCl_3_) δ 209.3 (CHO), 195.9 (C-5), 176.6 (C-2), 136.2 (=CH), 116.8 (=CH_2_), 60.5 (C-7a), 52.9 (C-3), 50.6 (NCH_2_), 45.7 (C-3a), 44.6 (C-4), 35.1 (C-6), 29.5 (CH_2_–3), 23.9 (Me), 23.4 (C-7); HRMS (ESI-TOF) *m*/*z* [M + H]^+^ calcd for C_14_H_20_NO_3_ 250.1443, found 250.1451.

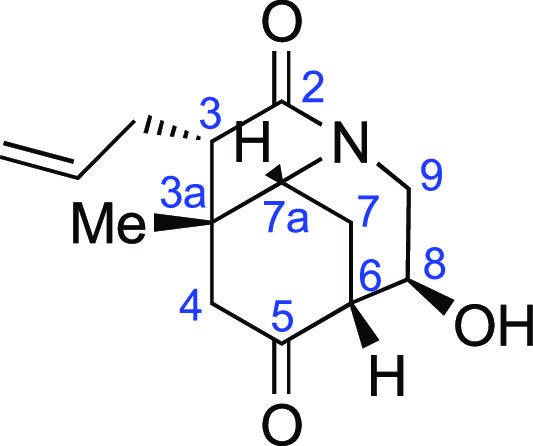



### (3*RS*,3a*SR*,6*SR*,7a*SR*,8*RS*)-3-Allyl-8-hydroxy-3a-methyltetrahydro-6,1-ethanoindole-2,5(3*H*,6*H*)-dione (**7**)

A solution of compound **6** (172 mg, 0.93 mmol) and *p*TsOH·H_2_O (180 g, 0.93 mmol) in benzene (10 mL) was heated to reflux with a heating block for 15 min. The mixture was cooled to room temperature, and after the addition of water, it was extracted with CH_2_Cl_2_ (3 × 20 mL) and CHCl_3_:*i*-PrOH (4:1, 2 × 15 mL). The combined organic extracts were concentrated and purified by chromatography (CH_2_Cl_2_:EtOAc 9.5:0.5 → CH_2_Cl_2_:MeOH 9.5:0.5) to obtain **7** as a solid (141 mg, 82%) and subsequently *epi*-**7** (16 mg, 9%).

Compound **7**: mp 100–103 °C; IR (neat): 3407, 2925, 1705, 1451, 1423, 1125, 1063, 1044 cm^–1^; ^1^H NMR (400 MHz, CDCl_3_) δ 5.89 (dddd, *J* = 17.0, 10.1, 8.9, 5.2 Hz, 1H, =CH), 5.14 (ddt, *J* = 17, 0.8, 0.8 Hz, 1H, =CH_2_ H-*trans*), 5.04 (ddt, *J* = 10.1, 0.8, 0.8 Hz, 1H, =CH_2_ H-*cis*), 4.67 (t, *J* = 8.1 Hz, 1H, H-8), 4.30 (dd, *J* = 13.8, 8.6 Hz, 1H, H-9eq), 3.71 (d, *J* = 5.6 Hz, 1H, H-7a), 2.72 (br s, 1H, OH), 2.63–2.53 (m, 1H, 3-CH_2_) 2.55 (dd, *J* = 13.8, 7.2 Hz, 1H, H-9ax) 2.44 (d, *J* = 14.8 Hz, 1H H-4), 2.40 (ddd, *J* = 14.6, 5.7, 1.3 Hz, 1H, H-7), 2.35–2.30 (m, 2H, H-3, H-6), 2.13 (d, *J* = 14.8 Hz, 1H, H-4), 2.15–2.05 (m, 2H, H-7, 3-CH_2_), 1.29 (s, 3H, Me); ^13^C{^1^H} NMR (101 MHz, CDCl_3_) δ 210.0 (C-5), 174.6 (C-2), 136.5 (=CH), 116.4 (=CH_2_), 72.0 (C-8), 60.2 (C-7a), 50.2 (C-3), 49.0 (C-6), 47.5 (C-3a), 44.2 (C-4), 41.0 (C-9), 29.7 (3-CH_2_), 24.2 (Me), 18.8 (C-7). HRMS (ESI-TOF) *m*/*z*: [M + H]^+^ calcd for C_14_H_20_NO_3_ 250.1440, found 250.1438.

Compound *epi*-**7**: IR (neat) 3420, 2923, 1688, 1423, 1077, 909 cm^–1^; ^1^H NMR (400 MHz, CDCl_3_) δ 5.97–5.87 (m, 1H, =CH), 5.15 (d, *J* = 17.2 Hz, 1H, =CH_2_ H-*trans*), 5.06 (d, *J* = 10.4 Hz, 1H, =CH_2_ H-*cis*), 4.22 (t, *J* = 6.4 Hz, 1H, H-8), 4.05 (dd, *J* = 14.8, 1.2 Hz, 1H, H-9eq), 3.52 (d, *J* = 5.6 Hz, 1H, H-7a), 3.11 (dd, *J* = 14.8, 6.6 Hz, 1H, H-9ax), 2.67 (dddt, *J* = 14.8, 6.4, 5.2, 2.0 Hz, 1H, 3-CH_2_), 2.54 (t, *J* = 6.0 Hz, 1H, H-6), 2.48 (d, *J* = 14.4 Hz, 1H, H-4), 2.35 (ddd, *J* = 8.2, 6.6, 1.2 Hz, 1H, H-3), 2.28 (dd, *J* = 14.8, 5.2 Hz, 1H, H-7), 2.16 (d, *J* = 14.4 Hz, 1H, H-4), 2.19–2.12 (m, 1H,3-CH_2_), 1.75 (ddd, *J* = 14.8, 5.9, 1.2 Hz, 1H, H-7), 1.31 (s, 3H, Me); ^13^C{^1^H} NMR δ 215.9 (C-5), 174.6 (C-2), 136.4 (=CH), 116.5 (=CH_2_), 76.6 (C-8), 59.0 (C-7a), 50.4 (C-3), 47.9 (C-3a), 45.0 (C-4), 44.3 (C-9), 41.7 (C-6), 29.9 (CH_2_–3), 23.4 (Me), 23.0 (C-7); HRMS (ESI-TOF) *m*/*z* [M + H]^+^ calcd for C_14_H_20_NO_3_ 250.1440, found 250.1435.

### Compound **8**

To a cooled (0 °C) stirred solution of tricyclic alcohol **7** (89.4 mg, 0.36 mmol) in CH_2_Cl_2_ (2.6 mL) was sequentially added TsCl (205 mg, 1.08 mmol), Et_3_N (40 μL, 0.54 mmol), and DMAP (110 mg, 0.90 mmol), and the reaction was stirred at room temperature overnight. After quenching with NaHCO_3_, the mixture was extracted with CH_2_Cl_2_ (4 × 10 mL); the combined organics were dried, filtered, concentrated, and purified by chromatography (CH_2_Cl_2_:EtOAc, 1:0 → 9.5:0.5) to afford **8** (126 mg, 87%) as a white solid.

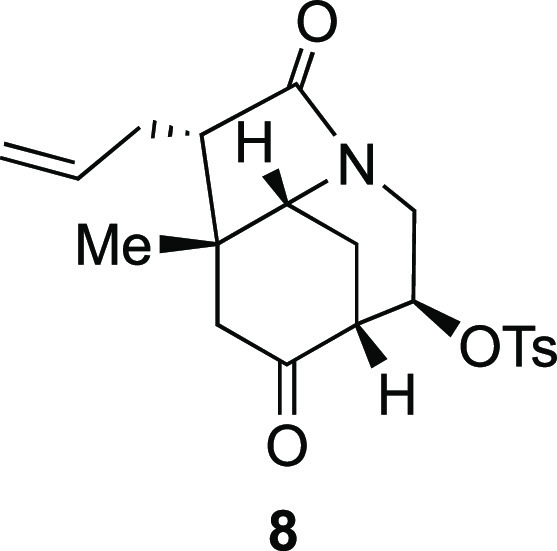



Mp 70–71 °C. IR (neat) 2959, 1711, 1698, 1415, 1362, 1177, 910 cm^–1^; ^1^H NMR (400 MHz, CDCl_3_) δ 7.81 and 7.37 (2d, *J* = 8.4 Hz, 2H each, Ts), 5.85 (dddd, *J* = 17.0, 10.0, 8.8, 5.2 Hz, 1H, =CH), 5.12 (dm, *J* = 17.0 Hz, 1H, =CH_2_ H-*trans*), 5.06 (ddt, *J* = 8.7, 7.0, 1.5 Hz, 1H, H-8), 5.04 (dm, *J* = 10.0 Hz, 1H, =CH_2_ H-*cis*), 4.23 (dd, *J* = 14.4, 9.0 Hz, H-9), 3.69 (br d, *J* = 5.6 Hz, 1H, H-7a), 2.68 (dd, *J* = 14.4, 7.0 Hz, H-9), 2.59 (br d, *J* = 5.0 Hz, 1H, H-6), 2.57–2.52 (m, 1H, 3-CH_2_), 2.46 (s, 3H, Me-Ts), 2.41 (d, *J* = 14.4 Hz, 1H, H-4), 2.36 (ddd, *J* = 15.0, 5.8, 1.4 Hz, 1H, H-7), 2.28 (dd, *J* = 8.4, 6.5 Hz, 1H, H-3), 2.17 (ddd, *J* = 15.0, 4.8, 1.4 Hz, H-7), 2.11 (d, *J* = 14.4 Hz, 1H, H-4), 2.09–2.04 (m, 1H, 3-CH_2_), 1.27 (s, 3H, Me); ^13^C{^1^H} NMR (101 MHz, CDCl_3_) δ 206.9 (C-6), 174.6 (C-2), 145.5, 132.6, 130.2, and 128.0 (Ph), 136.2 (=CH), 116.6 (=CH_2_), 79.7 (C-8), 59.6 (C-7a), 49.9 (C-3), 47.9 (C-3a), 45.6 (C-6), 43.8 (C-4), 38.6 (C-9), 29.5 (CH_2_–3), 24.0 (Me), 21.7 (Me-Ts), 19.0 (C-7); HRMS (ESI-TOF) *m*/*z* [M + H]^+^ calcd for C_21_H_26_NO_5_S 404.1532, found 404.1537.

### (3*RS*,3a*SR*,6S*R*,7a*SR*,8*RS*)-3-Allyl-3a,8-dimethyltetrahydro-6,1-ethanoindole-2,5(3*H*, 4*H*)-dione (**9**)

To a suspension of CuI (213 mg, 1.11 mmol) in Et_2_O (4.0 mL) was added dropwise a MeLi solution (1.6 M in Et_2_O, 1.26 mL) at −20 °C. The reaction was warmed to 0 °C, and after stirring for 30 min, a solution of tosylate **8** (45.1 mg, 0.11 mmol) in a 9:1 mixture of Et_2_O-THF (5 mL) was added dropwise via a cannula. The stirring was prolonged for 1 h 30 min, an Na_2_CO_3_ aqueous saturated solution was added, and the mixture was extracted with Et_2_O (4 × 10 mL). The combined organic extracts were dried over Na_2_SO_4_, filtered, and concentrated under vacuum to afford the crude product, which was purified by chromatography (hexane:EtOAc, 4:1→ 7:3) to obtain tricycle ring **9** (14 mg, 51%) as a white solid.

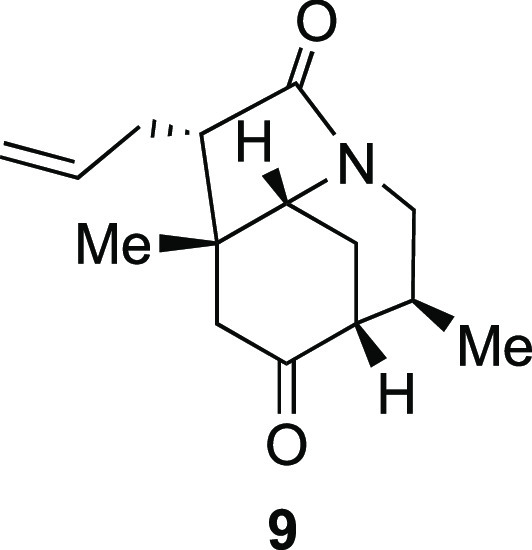



Mp 95–97 °C. IR (neat) 2957, 1708, 1691, 1423, 1290, 911 cm^–1^. The NMR data were identical with those previously reported by Gao:^4^^1^H NMR (400 MHz, CDCl_3_) δ 5.95–5.85 (m, 1H, =CH), 5.15 (d, *J* = 16.8 Hz, 1H, =CH_2_ H-*trans*), 5.03 (d, *J* = 9.8 Hz, 1H, =CH_2_ H-*cis*), 4.02 (dd, *J* = 13.6, 8.8 Hz, 1H, H-9), 3.66 (t, *J* = 3.0 Hz, 1H, H-7a), 2.91 (tq *J* = 8.8, 6.4 Hz, 1H, H-8), 2.62 (dm, *J* = 15.0 Hz, 1H, 3-CH_2_), 2.47 (d, *J* = 14.4 Hz, 1H, H-4), 2.32 (dd, *J* = 8.4, 6.4 Hz, 1H, H-3), 2.21 (dd, *J* = 13.6, 9.0 Hz, 1H, H-9), 2.14 (d, *J* = 14.4 Hz, 1H, H-4), 2.17–2.09 (m, 3H, H-7 and 3-CH_2_), 1.93 (t, *J* = 3.1 Hz, 1H, H-6), 1.28 (s, 3H, 3a-Me), 1.01 (d, *J* = 6.8 Hz, 3H, 8-Me); ^13^C{^1^H} NMR (101 MHz, CDCl_3_) δ 213.0 (C-5), 174.2 (C-2), 136.7 (=CH), 116.3 (=CH_2_), 60.2 (C-7a), 50.4 (C-3), 47.3 (C-3a), 46.0 (C-6), 44.4 (C-4), 40.6 (C-9), 36.6 (C-8), 30.0 (3-CH_2_), 24.4 (3a-Me), 19.6 (C-7), 17.8 (8-Me); HRMS (ESI-TOF) *m*/*z* [M + H]^+^ calcd for C_15_H_22_NO_2_ 248.1651, found 248.1660.

## Supporting Information Available

^1^H and ^13^C NMR spectra copies of all reported compounds; X-ray crystallographic data of tosylate **8**, and DFT calculations for the diastereoselectivity in the aldol cyclization of **6** (PDF)
FAIR data, including the primary NMR FID files, for compounds **2**–**10** (ZIP)
