# Supplementary material for: Synthesis of the ABC Ring of Calyciphylline A-Type Alkaloids by a Stereocontrolled Aldol Cyclization: Formal Synthesis of (±)-Himalensine A
Source: J Org Chem. 2022 Jul 21;87(15):10516–22. doi: 10.1021/acs.joc.2c01171 (PMC9881646; doi:10.1021/acs.joc.2c01171)
Supplement: Supplementary file 1 — jo2c01171_si_001.zip [file jo2c01171_si_001.zip › FID for publication/structure file_BONJOCH.docx]

The spectrometers used to collect the data are:

- **Bruker 400 MHz**
- **Varian VNMRS 400 MHz**
- **Varian Mercury 400 MHz**

The acquisition software used is VnmrJ.

The processing program used to analyze the data is MestreNova.

**Structure file**

**Compound (2)**

Compound identifier (MolFile):

O=C(C(Cl)(Cl)Cl)N(CC(OCC)OCC)C1=C(C)CC2(OCCO2)CC1

OpenBabel05162212553D

52 53 0 0 0 0 0 0 0 0999 V2000

10.0253 2.4370 -1.8167 O 0 0 0 0 0 0 0 0 0 0 0 0

9.0499 2.5824 -1.0338 C 0 0 0 0 0 0 0 0 0 0 0 0

8.5625 4.0476 -0.8319 C 0 0 0 0 0 0 0 0 0 0 0 0

7.2003 4.4434 0.2139 Cl 0 0 0 0 0 0 0 0 0 0 0 0

9.9733 4.9969 -0.2196 Cl 0 0 0 0 0 0 0 0 0 0 0 0

8.1515 4.6768 -2.4866 Cl 0 0 0 0 0 0 0 0 0 0 0 0

8.5012 1.4074 -0.4703 N 0 0 0 0 0 0 0 0 0 0 0 0

7.2377 1.2965 0.2958 C 0 0 0 0 0 0 0 0 0 0 0 0

7.3844 0.9038 1.7978 C 0 0 0 0 0 0 0 0 0 0 0 0

8.1343 1.9264 2.4755 O 0 0 0 0 0 0 0 0 0 0 0 0

8.4917 1.5948 3.8240 C 0 0 0 0 0 0 0 0 0 0 0 0

7.3447 1.7141 4.8177 C 0 0 0 0 0 0 0 0 0 0 0 0

6.0926 0.8113 2.4049 O 0 0 0 0 0 0 0 0 0 0 0 0

5.2953 -0.2761 1.9596 C 0 0 0 0 0 0 0 0 0 0 0 0

4.0737 -0.3831 2.8553 C 0 0 0 0 0 0 0 0 0 0 0 0

9.0874 0.1716 -0.8428 C 0 0 0 0 0 0 0 0 0 0 0 0

10.3524 -0.1542 -0.5046 C 0 0 0 0 0 0 0 0 0 0 0 0

11.2572 0.6277 0.4110 C 0 0 0 0 0 0 0 0 0 0 0 0

10.9897 -1.4374 -1.0024 C 0 0 0 0 0 0 0 0 0 0 0 0

9.9625 -2.5387 -1.3112 C 0 0 0 0 0 0 0 0 0 0 0 0

10.6362 -3.6807 -1.8616 O 0 0 0 0 0 0 0 0 0 0 0 0

10.9558 -4.4668 -0.7124 C 0 0 0 0 0 0 0 0 0 0 0 0

9.7504 -4.2732 0.1681 C 0 0 0 0 0 0 0 0 0 0 0 0

9.3146 -2.9369 -0.0868 O 0 0 0 0 0 0 0 0 0 0 0 0

8.9456 -1.9821 -2.2937 C 0 0 0 0 0 0 0 0 0 0 0 0

8.2362 -0.7451 -1.7253 C 0 0 0 0 0 0 0 0 0 0 0 0

6.6124 0.5576 -0.2095 H 0 0 0 0 0 0 0 0 0 0 0 0

6.6332 2.1851 0.2115 H 0 0 0 0 0 0 0 0 0 0 0 0

7.9195 -0.0492 1.8966 H 0 0 0 0 0 0 0 0 0 0 0 0

9.2643 2.3144 4.1203 H 0 0 0 0 0 0 0 0 0 0 0 0

8.9512 0.6001 3.8695 H 0 0 0 0 0 0 0 0 0 0 0 0

6.6537 0.8694 4.7366 H 0 0 0 0 0 0 0 0 0 0 0 0

6.7702 2.6301 4.6517 H 0 0 0 0 0 0 0 0 0 0 0 0

7.7212 1.7132 5.8461 H 0 0 0 0 0 0 0 0 0 0 0 0

5.8666 -1.2113 1.9832 H 0 0 0 0 0 0 0 0 0 0 0 0

4.9579 -0.0832 0.9371 H 0 0 0 0 0 0 0 0 0 0 0 0

4.2791 -1.0518 3.6989 H 0 0 0 0 0 0 0 0 0 0 0 0

3.2279 -0.8017 2.3007 H 0 0 0 0 0 0 0 0 0 0 0 0

3.7836 0.5873 3.2699 H 0 0 0 0 0 0 0 0 0 0 0 0

10.8087 1.5498 0.7834 H 0 0 0 0 0 0 0 0 0 0 0 0

12.1894 0.8790 -0.1011 H 0 0 0 0 0 0 0 0 0 0 0 0

11.4987 0.0157 1.2854 H 0 0 0 0 0 0 0 0 0 0 0 0

11.6979 -1.8065 -0.2489 H 0 0 0 0 0 0 0 0 0 0 0 0

11.5868 -1.2183 -1.8965 H 0 0 0 0 0 0 0 0 0 0 0 0

11.8654 -4.0648 -0.2505 H 0 0 0 0 0 0 0 0 0 0 0 0

11.1341 -5.5100 -0.9817 H 0 0 0 0 0 0 0 0 0 0 0 0

9.9676 -4.4049 1.2318 H 0 0 0 0 0 0 0 0 0 0 0 0

8.9382 -4.9478 -0.1176 H 0 0 0 0 0 0 0 0 0 0 0 0

8.1916 -2.7462 -2.5407 H 0 0 0 0 0 0 0 0 0 0 0 0

9.4353 -1.7347 -3.2451 H 0 0 0 0 0 0 0 0 0 0 0 0

7.3800 -1.1085 -1.1460 H 0 0 0 0 0 0 0 0 0 0 0 0

7.8325 -0.1640 -2.5636 H 0 0 0 0 0 0 0 0 0 0 0 0

1 2 2 0 0 0 0

2 3 1 0 0 0 0

2 7 1 0 0 0 0

3 4 1 0 0 0 0

3 5 1 0 0 0 0

3 6 1 0 0 0 0

7 8 1 0 0 0 0

7 16 1 0 0 0 0

8 9 1 0 0 0 0

8 27 1 0 0 0 0

8 28 1 0 0 0 0

9 10 1 0 0 0 0

9 13 1 0 0 0 0

9 29 1 0 0 0 0

10 11 1 0 0 0 0

11 12 1 0 0 0 0

11 30 1 0 0 0 0

11 31 1 0 0 0 0

12 32 1 0 0 0 0

12 33 1 0 0 0 0

12 34 1 0 0 0 0

13 14 1 0 0 0 0

14 15 1 0 0 0 0

14 35 1 0 0 0 0

14 36 1 0 0 0 0

15 37 1 0 0 0 0

15 38 1 0 0 0 0

15 39 1 0 0 0 0

16 17 2 0 0 0 0

16 26 1 0 0 0 0

17 18 1 0 0 0 0

17 19 1 0 0 0 0

18 40 1 0 0 0 0

18 41 1 0 0 0 0

18 42 1 0 0 0 0

19 20 1 0 0 0 0

19 43 1 0 0 0 0

19 44 1 0 0 0 0

20 21 1 0 0 0 0

20 24 1 0 0 0 0

20 25 1 0 0 0 0

21 22 1 0 0 0 0

22 23 1 0 0 0 0

22 45 1 0 0 0 0

22 46 1 0 0 0 0

23 24 1 0 0 0 0

23 47 1 0 0 0 0

23 48 1 0 0 0 0

25 26 1 0 0 0 0

25 49 1 0 0 0 0

25 50 1 0 0 0 0

26 51 1 0 0 0 0

26 52 1 0 0 0 0

M END

**Compound (3).**

Compound identifier (MolFile):

CC(CCc1ccccc1)C(O)c2ccc(C#N)cc2

OpenBabel05162212453D

50 52 0 0 1 0 0 0 0 0999 V2000

3.7620 0.9747 3.7916 O 0 0 0 0 0 0 0 0 0 0 0 0

4.8440 0.5525 3.3893 C 0 0 0 0 0 0 0 0 0 0 0 0

5.0618 -0.3229 2.3295 N 0 0 0 0 0 0 0 0 0 0 0 0

3.9870 -0.7817 1.4541 C 0 0 0 0 0 0 0 0 0 0 0 0

3.1647 0.3282 0.7269 C 0 0 0 0 0 0 0 0 0 0 0 0

4.0290 0.9965 -0.2193 O 0 0 0 0 0 0 0 0 0 0 0 0

3.3635 2.0356 -0.9410 C 0 0 0 0 0 0 0 0 0 0 0 0

4.3598 2.8033 -1.8007 C 0 0 0 0 0 0 0 0 0 0 0 0

2.0344 -0.2491 0.0358 O 0 0 0 0 0 0 0 0 0 0 0 0

1.0125 -0.7198 0.9163 C 0 0 0 0 0 0 0 0 0 0 0 0

-0.1952 -1.1870 0.1223 C 0 0 0 0 0 0 0 0 0 0 0 0

6.3925 -0.6511 2.2227 C 0 0 0 0 0 0 0 0 0 0 0 0

7.0101 -1.1955 1.1689 C 0 0 0 0 0 0 0 0 0 0 0 0

8.5020 -1.0545 1.0525 C 0 0 0 0 0 0 0 0 0 0 0 0

8.9905 0.2465 1.7402 C 0 0 0 0 0 0 0 0 0 0 0 0

10.4197 0.3458 1.6038 O 0 0 0 0 0 0 0 0 0 0 0 0

10.5969 1.1920 0.4648 C 0 0 0 0 0 0 0 0 0 0 0 0

9.4744 2.1797 0.6132 C 0 0 0 0 0 0 0 0 0 0 0 0

8.3900 1.3917 1.0992 O 0 0 0 0 0 0 0 0 0 0 0 0

8.5988 0.2375 3.2250 C 0 0 0 0 0 0 0 0 0 0 0 0

7.1221 -0.1702 3.4625 C 0 0 1 0 0 0 0 0 0 0 0 0

7.0758 -1.2987 4.5225 C 0 0 0 0 0 0 0 0 0 0 0 0

6.2044 0.9738 3.9178 C 0 0 0 0 0 0 0 0 0 0 0 0

3.3275 -1.3683 2.1003 H 0 0 0 0 0 0 0 0 0 0 0 0

4.3766 -1.4845 0.7109 H 0 0 0 0 0 0 0 0 0 0 0 0

2.8028 1.0728 1.4442 H 0 0 0 0 0 0 0 0 0 0 0 0

2.8699 2.7275 -0.2494 H 0 0 0 0 0 0 0 0 0 0 0 0

2.6118 1.5928 -1.6022 H 0 0 0 0 0 0 0 0 0 0 0 0

5.2405 3.1144 -1.2295 H 0 0 0 0 0 0 0 0 0 0 0 0

3.8910 3.6932 -2.2358 H 0 0 0 0 0 0 0 0 0 0 0 0

4.7158 2.1760 -2.6249 H 0 0 0 0 0 0 0 0 0 0 0 0

0.7023 0.0774 1.5992 H 0 0 0 0 0 0 0 0 0 0 0 0

1.3832 -1.5686 1.4956 H 0 0 0 0 0 0 0 0 0 0 0 0

-0.6310 -0.3571 -0.4416 H 0 0 0 0 0 0 0 0 0 0 0 0

-0.9594 -1.5879 0.7969 H 0 0 0 0 0 0 0 0 0 0 0 0

0.0760 -1.9656 -0.5982 H 0 0 0 0 0 0 0 0 0 0 0 0

6.4819 -1.5481 0.2921 H 0 0 0 0 0 0 0 0 0 0 0 0

8.7569 -1.0150 -0.0128 H 0 0 0 0 0 0 0 0 0 0 0 0

9.0104 -1.9339 1.4656 H 0 0 0 0 0 0 0 0 0 0 0 0

10.4837 0.5870 -0.4422 H 0 0 0 0 0 0 0 0 0 0 0 0

11.5871 1.6530 0.4655 H 0 0 0 0 0 0 0 0 0 0 0 0

9.2007 2.6744 -0.3222 H 0 0 0 0 0 0 0 0 0 0 0 0

9.7057 2.9379 1.3685 H 0 0 0 0 0 0 0 0 0 0 0 0

8.8029 1.2169 3.6766 H 0 0 0 0 0 0 0 0 0 0 0 0

9.2713 -0.4603 3.7442 H 0 0 0 0 0 0 0 0 0 0 0 0

7.5477 -0.9796 5.4586 H 0 0 0 0 0 0 0 0 0 0 0 0

7.5988 -2.1950 4.1685 H 0 0 0 0 0 0 0 0 0 0 0 0

6.0480 -1.5990 4.7615 H 0 0 0 0 0 0 0 0 0 0 0 0

6.4699 1.9305 3.4582 H 0 0 0 0 0 0 0 0 0 0 0 0

6.1793 1.0856 5.0078 H 0 0 0 0 0 0 0 0 0 0 0 0

1 2 2 0 0 0 0

2 3 1 0 0 0 0

2 23 1 0 0 0 0

3 4 1 0 0 0 0

3 12 1 0 0 0 0

4 5 1 0 0 0 0

4 24 1 0 0 0 0

4 25 1 0 0 0 0

5 6 1 0 0 0 0

5 9 1 0 0 0 0

5 26 1 0 0 0 0

6 7 1 0 0 0 0

7 8 1 0 0 0 0

7 27 1 0 0 0 0

7 28 1 0 0 0 0

8 29 1 0 0 0 0

8 30 1 0 0 0 0

8 31 1 0 0 0 0

9 10 1 0 0 0 0

10 11 1 0 0 0 0

10 32 1 0 0 0 0

10 33 1 0 0 0 0

11 34 1 0 0 0 0

11 35 1 0 0 0 0

11 36 1 0 0 0 0

12 13 2 0 0 0 0

12 21 1 0 0 0 0

13 14 1 0 0 0 0

13 37 1 0 0 0 0

14 15 1 0 0 0 0

14 38 1 0 0 0 0

14 39 1 0 0 0 0

15 16 1 0 0 0 0

15 19 1 0 0 0 0

15 20 1 0 0 0 0

16 17 1 0 0 0 0

17 18 1 0 0 0 0

17 40 1 0 0 0 0

17 41 1 0 0 0 0

18 19 1 0 0 0 0

18 42 1 0 0 0 0

18 43 1 0 0 0 0

20 21 1 0 0 0 0

20 44 1 0 0 0 0

20 45 1 0 0 0 0

21 22 1 6 0 0 0

21 23 1 0 0 0 0

22 46 1 0 0 0 0

22 47 1 0 0 0 0

22 48 1 0 0 0 0

23 49 1 0 0 0 0

23 50 1 0 0 0 0

M END

**Compound (3’)**

Compound identifier (MolFile):

O=C1N(CC(OCC)OCC)C2(O)CCC3(OCCO3)C[C@]2(C)C1

OpenBabel05162213003D

53 55 0 0 1 0 0 0 0 0999 V2000

3.3139 -1.7487 -2.2944 O 0 0 0 0 0 0 0 0 0 0 0 0

4.3455 -1.5035 -1.6790 C 0 0 0 0 0 0 0 0 0 0 0 0

4.5119 -1.4841 -0.3001 N 0 0 0 0 0 0 0 0 0 0 0 0

3.3777 -1.5396 0.6186 C 0 0 0 0 0 0 0 0 0 0 0 0

2.8769 -0.1821 1.1847 C 0 0 0 0 0 0 0 0 0 0 0 0

2.3402 0.6619 0.1580 O 0 0 0 0 0 0 0 0 0 0 0 0

2.0588 1.9703 0.6540 C 0 0 0 0 0 0 0 0 0 0 0 0

1.5095 2.8458 -0.4568 C 0 0 0 0 0 0 0 0 0 0 0 0

1.9345 -0.4003 2.2563 O 0 0 0 0 0 0 0 0 0 0 0 0

0.7091 -0.9847 1.8168 C 0 0 0 0 0 0 0 0 0 0 0 0

-0.2541 -1.0880 2.9836 C 0 0 0 0 0 0 0 0 0 0 0 0

5.8224 -1.0082 0.0647 C 0 0 3 0 0 0 0 0 0 0 0 0

5.7004 0.4021 0.1664 O 0 0 0 0 0 0 0 0 0 0 0 0

6.3604 -1.5538 1.3894 C 0 0 0 0 0 0 0 0 0 0 0 0

6.6103 -3.0542 1.3405 C 0 0 0 0 0 0 0 0 0 0 0 0

7.5922 -3.4154 0.2236 C 0 0 0 0 0 0 0 0 0 0 0 0

7.7166 -4.8448 0.1951 O 0 0 0 0 0 0 0 0 0 0 0 0

9.0831 -5.1254 0.5010 C 0 0 0 0 0 0 0 0 0 0 0 0

9.5808 -3.8816 1.1853 C 0 0 0 0 0 0 0 0 0 0 0 0

8.8735 -2.8396 0.5125 O 0 0 0 0 0 0 0 0 0 0 0 0

7.0823 -2.9137 -1.1325 C 0 0 0 0 0 0 0 0 0 0 0 0

6.6785 -1.4059 -1.1635 C 0 0 1 0 0 0 0 0 0 0 0 0

7.9123 -0.5125 -1.3556 C 0 0 0 0 0 0 0 0 0 0 0 0

5.6747 -1.1752 -2.3184 C 0 0 0 0 0 0 0 0 0 0 0 0

3.6509 -2.1986 1.4506 H 0 0 0 0 0 0 0 0 0 0 0 0

2.5608 -2.0390 0.0877 H 0 0 0 0 0 0 0 0 0 0 0 0

3.7289 0.3303 1.6450 H 0 0 0 0 0 0 0 0 0 0 0 0

2.9715 2.4223 1.0568 H 0 0 0 0 0 0 0 0 0 0 0 0

1.3108 1.9078 1.4509 H 0 0 0 0 0 0 0 0 0 0 0 0

0.6025 2.4111 -0.8894 H 0 0 0 0 0 0 0 0 0 0 0 0

2.2361 2.9548 -1.2678 H 0 0 0 0 0 0 0 0 0 0 0 0

1.2676 3.8404 -0.0682 H 0 0 0 0 0 0 0 0 0 0 0 0

0.8892 -1.9894 1.4268 H 0 0 0 0 0 0 0 0 0 0 0 0

0.2562 -0.3681 1.0348 H 0 0 0 0 0 0 0 0 0 0 0 0

-0.3514 -0.1320 3.5072 H 0 0 0 0 0 0 0 0 0 0 0 0

0.0997 -1.8241 3.7129 H 0 0 0 0 0 0 0 0 0 0 0 0

-1.2402 -1.4021 2.6284 H 0 0 0 0 0 0 0 0 0 0 0 0

6.5362 0.7574 0.5088 H 0 0 0 0 0 0 0 0 0 0 0 0

5.6793 -1.3168 2.2146 H 0 0 0 0 0 0 0 0 0 0 0 0

7.2956 -1.0364 1.6412 H 0 0 0 0 0 0 0 0 0 0 0 0

5.6704 -3.6025 1.2031 H 0 0 0 0 0 0 0 0 0 0 0 0

7.0029 -3.3766 2.3122 H 0 0 0 0 0 0 0 0 0 0 0 0

9.6106 -5.3010 -0.4418 H 0 0 0 0 0 0 0 0 0 0 0 0

9.1593 -6.0190 1.1265 H 0 0 0 0 0 0 0 0 0 0 0 0

10.6607 -3.7402 1.1003 H 0 0 0 0 0 0 0 0 0 0 0 0

9.3026 -3.8622 2.2448 H 0 0 0 0 0 0 0 0 0 0 0 0

6.2269 -3.5398 -1.4230 H 0 0 0 0 0 0 0 0 0 0 0 0

7.8428 -3.1035 -1.9026 H 0 0 0 0 0 0 0 0 0 0 0 0

8.5102 -0.8419 -2.2118 H 0 0 0 0 0 0 0 0 0 0 0 0

8.5551 -0.5169 -0.4708 H 0 0 0 0 0 0 0 0 0 0 0 0

7.6313 0.5299 -1.5440 H 0 0 0 0 0 0 0 0 0 0 0 0

5.8653 -1.8250 -3.1775 H 0 0 0 0 0 0 0 0 0 0 0 0

5.6417 -0.1307 -2.6471 H 0 0 0 0 0 0 0 0 0 0 0 0

1 2 2 0 0 0 0

2 3 1 0 0 0 0

2 24 1 0 0 0 0

3 4 1 0 0 0 0

3 12 1 0 0 0 0

4 5 1 0 0 0 0

4 25 1 0 0 0 0

4 26 1 0 0 0 0

5 6 1 0 0 0 0

5 9 1 0 0 0 0

5 27 1 0 0 0 0

6 7 1 0 0 0 0

7 8 1 0 0 0 0

7 28 1 0 0 0 0

7 29 1 0 0 0 0

8 30 1 0 0 0 0

8 31 1 0 0 0 0

8 32 1 0 0 0 0

9 10 1 0 0 0 0

10 11 1 0 0 0 0

10 33 1 0 0 0 0

10 34 1 0 0 0 0

11 35 1 0 0 0 0

11 36 1 0 0 0 0

11 37 1 0 0 0 0

12 13 1 0 0 0 0

12 14 1 0 0 0 0

12 22 1 0 0 0 0

13 38 1 0 0 0 0

14 15 1 0 0 0 0

14 39 1 0 0 0 0

14 40 1 0 0 0 0

15 16 1 0 0 0 0

15 41 1 0 0 0 0

15 42 1 0 0 0 0

16 17 1 0 0 0 0

16 20 1 0 0 0 0

16 21 1 0 0 0 0

17 18 1 0 0 0 0

18 19 1 0 0 0 0

18 43 1 0 0 0 0

18 44 1 0 0 0 0

19 20 1 0 0 0 0

19 45 1 0 0 0 0

19 46 1 0 0 0 0

21 22 1 0 0 0 0

21 47 1 0 0 0 0

21 48 1 0 0 0 0

22 23 1 1 0 0 0

22 24 1 0 0 0 0

23 49 1 0 0 0 0

23 50 1 0 0 0 0

23 51 1 0 0 0 0

24 52 1 0 0 0 0

24 53 1 0 0 0 0

M END

**Compound (4).**

Compound identifier (MolFile):

O=C1N(CC(OCC)OCC)C2=CCC3(OCCO3)C[C@]2(C)[C@@H]1CC=C

OpenBabel05162213103D

57 59 0 0 1 0 0 0 0 0999 V2000

3.2446 0.7554 -1.3734 O 0 0 0 0 0 0 0 0 0 0 0 0

4.1867 0.3078 -0.7577 C 0 0 0 0 0 0 0 0 0 0 0 0

4.1630 -0.3868 0.4638 N 0 0 0 0 0 0 0 0 0 0 0 0

2.9226 -0.6160 1.2027 C 0 0 0 0 0 0 0 0 0 0 0 0

2.5470 -2.1169 1.3955 C 0 0 0 0 0 0 0 0 0 0 0 0

1.3500 -2.2197 2.1850 O 0 0 0 0 0 0 0 0 0 0 0 0

0.9401 -3.5633 2.4636 C 0 0 0 0 0 0 0 0 0 0 0 0

1.9274 -4.3096 3.3431 C 0 0 0 0 0 0 0 0 0 0 0 0

2.4309 -2.8302 0.1573 O 0 0 0 0 0 0 0 0 0 0 0 0

1.4315 -2.2933 -0.7036 C 0 0 0 0 0 0 0 0 0 0 0 0

1.3868 -3.0897 -1.9879 C 0 0 0 0 0 0 0 0 0 0 0 0

5.4300 -0.7330 0.8802 C 0 0 0 0 0 0 0 0 0 0 0 0

5.8136 -1.0407 2.1196 C 0 0 0 0 0 0 0 0 0 0 0 0

7.2643 -1.2263 2.4968 C 0 0 0 0 0 0 0 0 0 0 0 0

8.2986 -1.1102 1.3481 C 0 0 0 0 0 0 0 0 0 0 0 0

9.4913 -0.4813 1.8747 O 0 0 0 0 0 0 0 0 0 0 0 0

10.5713 -1.2678 1.3765 C 0 0 0 0 0 0 0 0 0 0 0 0

9.9771 -2.6460 1.3343 C 0 0 0 0 0 0 0 0 0 0 0 0

8.6513 -2.4144 0.8550 O 0 0 0 0 0 0 0 0 0 0 0 0

7.7970 -0.2290 0.1926 C 0 0 0 0 0 0 0 0 0 0 0 0

6.3779 -0.5803 -0.2869 C 0 0 1 0 0 0 0 0 0 0 0 0

6.3738 -1.9093 -1.0862 C 0 0 0 0 0 0 0 0 0 0 0 0

5.6629 0.5086 -1.1402 C 0 0 2 0 0 0 0 0 0 0 0 0

5.7761 0.2802 -2.2061 H 0 0 0 0 0 0 0 0 0 0 0 0

6.0537 1.9853 -0.9196 C 0 0 0 0 0 0 0 0 0 0 0 0

5.7693 2.5421 0.4525 C 0 0 0 0 0 0 0 0 0 0 0 0

4.9496 3.5697 0.7006 C 0 0 0 0 0 0 0 0 0 0 0 0

2.1055 -0.1012 0.6865 H 0 0 0 0 0 0 0 0 0 0 0 0

2.9966 -0.1084 2.1718 H 0 0 0 0 0 0 0 0 0 0 0 0

3.3576 -2.5869 1.9611 H 0 0 0 0 0 0 0 0 0 0 0 0

0.7522 -4.1154 1.5359 H 0 0 0 0 0 0 0 0 0 0 0 0

-0.0214 -3.4994 2.9864 H 0 0 0 0 0 0 0 0 0 0 0 0

1.4849 -5.2314 3.7321 H 0 0 0 0 0 0 0 0 0 0 0 0

2.2540 -3.6851 4.1800 H 0 0 0 0 0 0 0 0 0 0 0 0

2.8181 -4.5878 2.7712 H 0 0 0 0 0 0 0 0 0 0 0 0

1.6699 -1.2634 -0.9646 H 0 0 0 0 0 0 0 0 0 0 0 0

0.4535 -2.3200 -0.2129 H 0 0 0 0 0 0 0 0 0 0 0 0

2.3943 -3.2890 -2.3660 H 0 0 0 0 0 0 0 0 0 0 0 0

0.8230 -2.5369 -2.7451 H 0 0 0 0 0 0 0 0 0 0 0 0

0.8957 -4.0533 -1.8248 H 0 0 0 0 0 0 0 0 0 0 0 0

5.1002 -1.0787 2.9370 H 0 0 0 0 0 0 0 0 0 0 0 0

7.3684 -2.1991 2.9917 H 0 0 0 0 0 0 0 0 0 0 0 0

7.4912 -0.4647 3.2546 H 0 0 0 0 0 0 0 0 0 0 0 0

11.4425 -1.1841 2.0302 H 0 0 0 0 0 0 0 0 0 0 0 0

10.8313 -0.9045 0.3758 H 0 0 0 0 0 0 0 0 0 0 0 0

9.9046 -3.0798 2.3375 H 0 0 0 0 0 0 0 0 0 0 0 0

10.5155 -3.3337 0.6781 H 0 0 0 0 0 0 0 0 0 0 0 0

8.4959 -0.2711 -0.6527 H 0 0 0 0 0 0 0 0 0 0 0 0

7.8468 0.8008 0.5561 H 0 0 0 0 0 0 0 0 0 0 0 0

6.6026 -2.7743 -0.4559 H 0 0 0 0 0 0 0 0 0 0 0 0

7.1043 -1.8770 -1.9020 H 0 0 0 0 0 0 0 0 0 0 0 0

5.3941 -2.1124 -1.5377 H 0 0 0 0 0 0 0 0 0 0 0 0

5.5371 2.5962 -1.6730 H 0 0 0 0 0 0 0 0 0 0 0 0

7.1234 2.1135 -1.1333 H 0 0 0 0 0 0 0 0 0 0 0 0

6.2441 2.0618 1.3039 H 0 0 0 0 0 0 0 0 0 0 0 0

4.4338 4.0997 -0.0936 H 0 0 0 0 0 0 0 0 0 0 0 0

4.7673 3.8903 1.7219 H 0 0 0 0 0 0 0 0 0 0 0 0

1 2 2 0 0 0 0

2 3 1 0 0 0 0

2 23 1 0 0 0 0

3 4 1 0 0 0 0

3 12 1 0 0 0 0

4 5 1 0 0 0 0

4 28 1 0 0 0 0

4 29 1 0 0 0 0

5 6 1 0 0 0 0

5 9 1 0 0 0 0

5 30 1 0 0 0 0

6 7 1 0 0 0 0

7 8 1 0 0 0 0

7 31 1 0 0 0 0

7 32 1 0 0 0 0

8 33 1 0 0 0 0

8 34 1 0 0 0 0

8 35 1 0 0 0 0

9 10 1 0 0 0 0

10 11 1 0 0 0 0

10 36 1 0 0 0 0

10 37 1 0 0 0 0

11 38 1 0 0 0 0

11 39 1 0 0 0 0

11 40 1 0 0 0 0

12 13 2 0 0 0 0

12 21 1 0 0 0 0

13 14 1 0 0 0 0

13 41 1 0 0 0 0

14 15 1 0 0 0 0

14 42 1 0 0 0 0

14 43 1 0 0 0 0

15 16 1 0 0 0 0

15 19 1 0 0 0 0

15 20 1 0 0 0 0

16 17 1 0 0 0 0

17 18 1 0 0 0 0

17 44 1 0 0 0 0

17 45 1 0 0 0 0

18 19 1 0 0 0 0

18 46 1 0 0 0 0

18 47 1 0 0 0 0

20 21 1 0 0 0 0

20 48 1 0 0 0 0

20 49 1 0 0 0 0

21 22 1 6 0 0 0

21 23 1 0 0 0 0

22 50 1 0 0 0 0

22 51 1 0 0 0 0

22 52 1 0 0 0 0

23 24 1 6 0 0 0

23 25 1 0 0 0 0

25 26 1 0 0 0 0

25 53 1 0 0 0 0

25 54 1 0 0 0 0

26 27 2 0 0 0 0

26 55 1 0 0 0 0

27 56 1 0 0 0 0

27 57 1 0 0 0 0

M END

**Compound (5)**

Compound identifier (MolFile):

O=C1N(CC(OCC)OCC)[C@]2([H])CCC3(OCCO3)C[C@]2(C)[C@@H]1CC=C

OpenBabel05162213123D

59 61 0 0 1 0 0 0 0 0999 V2000

3.8386 1.8802 1.0941 O 0 0 0 0 0 0 0 0 0 0 0 0

4.6879 2.0029 0.2434 C 0 0 0 0 0 0 0 0 0 0 0 0

4.7827 1.2747 -0.9420 N 0 0 0 0 0 0 0 0 0 0 0 0

3.7712 0.2881 -1.3566 C 0 0 0 0 0 0 0 0 0 0 0 0

3.1043 0.5589 -2.7445 C 0 0 0 0 0 0 0 0 0 0 0 0

2.6229 1.9057 -2.8613 O 0 0 0 0 0 0 0 0 0 0 0 0

1.6360 2.2437 -1.8921 C 0 0 0 0 0 0 0 0 0 0 0 0

1.0704 3.6137 -2.2070 C 0 0 0 0 0 0 0 0 0 0 0 0

4.0095 0.2366 -3.8296 O 0 0 0 0 0 0 0 0 0 0 0 0

3.4321 0.5696 -5.0989 C 0 0 0 0 0 0 0 0 0 0 0 0

4.3330 0.1860 -6.2637 C 0 0 0 0 0 0 0 0 0 0 0 0

5.9290 1.6212 -1.7550 C 0 0 2 0 0 0 0 0 0 0 0 0

5.5680 1.8038 -2.7705 H 0 0 0 0 0 0 0 0 0 0 0 0

6.9772 0.5016 -1.7968 C 0 0 0 0 0 0 0 0 0 0 0 0

8.3312 0.9032 -2.3986 C 0 0 0 0 0 0 0 0 0 0 0 0

8.5684 2.4136 -2.4757 C 0 0 0 0 0 0 0 0 0 0 0 0

8.0125 2.9031 -3.6975 O 0 0 0 0 0 0 0 0 0 0 0 0

9.0820 3.5789 -4.3560 C 0 0 0 0 0 0 0 0 0 0 0 0

10.3205 2.9256 -3.8077 C 0 0 0 0 0 0 0 0 0 0 0 0

9.9811 2.6747 -2.4505 O 0 0 0 0 0 0 0 0 0 0 0 0

7.9616 3.0938 -1.2596 C 0 0 0 0 0 0 0 0 0 0 0 0

6.4417 2.9514 -1.1440 C 0 0 1 0 0 0 0 0 0 0 0 0

5.8069 4.1420 -1.9155 C 0 0 0 0 0 0 0 0 0 0 0 0

5.8791 2.9613 0.3219 C 0 0 2 0 0 0 0 0 0 0 0 0

5.4777 3.9547 0.5622 H 0 0 0 0 0 0 0 0 0 0 0 0

6.7251 2.4557 1.5102 C 0 0 0 0 0 0 0 0 0 0 0 0

7.9848 3.2133 1.8384 C 0 0 0 0 0 0 0 0 0 0 0 0

9.2021 2.6482 1.8566 C 0 0 0 0 0 0 0 0 0 0 0 0

2.9859 0.2863 -0.5919 H 0 0 0 0 0 0 0 0 0 0 0 0

4.2316 -0.7055 -1.3369 H 0 0 0 0 0 0 0 0 0 0 0 0

2.2590 -0.1343 -2.8456 H 0 0 0 0 0 0 0 0 0 0 0 0

2.0896 2.2874 -0.9009 H 0 0 0 0 0 0 0 0 0 0 0 0

0.8347 1.4973 -1.8910 H 0 0 0 0 0 0 0 0 0 0 0 0

1.8243 4.3864 -2.0216 H 0 0 0 0 0 0 0 0 0 0 0 0

0.2042 3.8214 -1.5718 H 0 0 0 0 0 0 0 0 0 0 0 0

0.7751 3.6940 -3.2573 H 0 0 0 0 0 0 0 0 0 0 0 0

3.2826 1.6523 -5.1465 H 0 0 0 0 0 0 0 0 0 0 0 0

2.4653 0.0663 -5.2090 H 0 0 0 0 0 0 0 0 0 0 0 0

4.0067 0.7056 -7.1730 H 0 0 0 0 0 0 0 0 0 0 0 0

4.2837 -0.8887 -6.4677 H 0 0 0 0 0 0 0 0 0 0 0 0

5.3786 0.4424 -6.0714 H 0 0 0 0 0 0 0 0 0 0 0 0

6.5825 -0.3517 -2.3634 H 0 0 0 0 0 0 0 0 0 0 0 0

7.1511 0.1155 -0.7839 H 0 0 0 0 0 0 0 0 0 0 0 0

8.4222 0.4727 -3.4034 H 0 0 0 0 0 0 0 0 0 0 0 0

9.1246 0.4341 -1.8017 H 0 0 0 0 0 0 0 0 0 0 0 0

9.0290 4.6389 -4.0859 H 0 0 0 0 0 0 0 0 0 0 0 0

8.9874 3.4755 -5.4392 H 0 0 0 0 0 0 0 0 0 0 0 0

11.2105 3.5553 -3.8737 H 0 0 0 0 0 0 0 0 0 0 0 0

10.5185 1.9603 -4.2836 H 0 0 0 0 0 0 0 0 0 0 0 0

8.2551 4.1504 -1.2086 H 0 0 0 0 0 0 0 0 0 0 0 0

8.4581 2.6065 -0.4319 H 0 0 0 0 0 0 0 0 0 0 0 0

6.1602 4.1918 -2.9498 H 0 0 0 0 0 0 0 0 0 0 0 0

6.0767 5.0951 -1.4458 H 0 0 0 0 0 0 0 0 0 0 0 0

4.7154 4.0782 -1.9549 H 0 0 0 0 0 0 0 0 0 0 0 0

6.1028 2.5178 2.4149 H 0 0 0 0 0 0 0 0 0 0 0 0

6.9408 1.3877 1.3834 H 0 0 0 0 0 0 0 0 0 0 0 0

7.8893 4.2703 2.0739 H 0 0 0 0 0 0 0 0 0 0 0 0

9.3498 1.6031 1.5930 H 0 0 0 0 0 0 0 0 0 0 0 0

10.0905 3.2332 2.0703 H 0 0 0 0 0 0 0 0 0 0 0 0

1 2 2 0 0 0 0

2 3 1 0 0 0 0

2 24 1 0 0 0 0

3 4 1 0 0 0 0

3 12 1 0 0 0 0

4 5 1 0 0 0 0

4 29 1 0 0 0 0

4 30 1 0 0 0 0

5 6 1 0 0 0 0

5 9 1 0 0 0 0

5 31 1 0 0 0 0

6 7 1 0 0 0 0

7 8 1 0 0 0 0

7 32 1 0 0 0 0

7 33 1 0 0 0 0

8 34 1 0 0 0 0

8 35 1 0 0 0 0

8 36 1 0 0 0 0

9 10 1 0 0 0 0

10 11 1 0 0 0 0

10 37 1 0 0 0 0

10 38 1 0 0 0 0

11 39 1 0 0 0 0

11 40 1 0 0 0 0

11 41 1 0 0 0 0

12 13 1 6 0 0 0

12 14 1 0 0 0 0

12 22 1 0 0 0 0

14 15 1 0 0 0 0

14 42 1 0 0 0 0

14 43 1 0 0 0 0

15 16 1 0 0 0 0

15 44 1 0 0 0 0

15 45 1 0 0 0 0

16 17 1 0 0 0 0

16 20 1 0 0 0 0

16 21 1 0 0 0 0

17 18 1 0 0 0 0

18 19 1 0 0 0 0

18 46 1 0 0 0 0

18 47 1 0 0 0 0

19 20 1 0 0 0 0

19 48 1 0 0 0 0

19 49 1 0 0 0 0

21 22 1 0 0 0 0

21 50 1 0 0 0 0

21 51 1 0 0 0 0

22 23 1 6 0 0 0

22 24 1 0 0 0 0

23 52 1 0 0 0 0

23 53 1 0 0 0 0

23 54 1 0 0 0 0

24 26 1 0 0 0 0

24 25 1 1 0 0 0

26 27 1 0 0 0 0

26 55 1 0 0 0 0

26 56 1 0 0 0 0

27 28 2 0 0 0 0

27 57 1 0 0 0 0

28 58 1 0 0 0 0

28 59 1 0 0 0 0

M END

**Compound (6).**

Compound identifier (MolFile):

[H]C(CN(C([C@H]1CC=C)=O)[C@]([C@]1(C)C2)([H])CCC2=O)=O

OpenBabel05162213153D

37 38 0 0 1 0 0 0 0 0999 V2000

4.3247 1.8956 -1.3664 H 0 0 0 0 0 0 0 0 0 0 0 0

3.9796 1.3384 -2.2545 C 0 0 0 0 0 0 0 0 0 0 0 0

2.5037 1.4380 -2.5645 C 0 0 0 0 0 0 0 0 0 0 0 0

1.9586 2.7516 -2.2293 N 0 0 0 0 0 0 0 0 0 0 0 0

0.9005 2.9191 -1.3449 C 0 0 0 0 0 0 0 0 0 0 0 0

0.5547 4.3931 -1.2691 C 0 0 1 0 0 0 0 0 0 0 0 0

-0.5382 4.4913 -1.2383 H 0 0 0 0 0 0 0 0 0 0 0 0

1.1522 4.8994 0.0599 C 0 0 0 0 0 0 0 0 0 0 0 0

0.8579 6.3321 0.4108 C 0 0 0 0 0 0 0 0 0 0 0 0

-0.2368 6.7637 1.0483 C 0 0 0 0 0 0 0 0 0 0 0 0

0.3685 2.0304 -0.6947 O 0 0 0 0 0 0 0 0 0 0 0 0

2.2391 3.9384 -3.0132 C 0 0 1 0 0 0 0 0 0 0 0 0

1.1127 4.9401 -2.6296 C 0 0 1 0 0 0 0 0 0 0 0 0

-0.0142 4.8509 -3.6905 C 0 0 0 0 0 0 0 0 0 0 0 0

1.6161 6.3912 -2.5873 C 0 0 0 0 0 0 0 0 0 0 0 0

2.1565 3.6555 -4.0709 H 0 0 0 0 0 0 0 0 0 0 0 0

3.6387 4.5166 -2.7731 C 0 0 0 0 0 0 0 0 0 0 0 0

3.8732 5.7700 -3.6139 C 0 0 0 0 0 0 0 0 0 0 0 0

2.6415 6.6472 -3.6559 C 0 0 0 0 0 0 0 0 0 0 0 0

2.5315 7.5730 -4.4599 O 0 0 0 0 0 0 0 0 0 0 0 0

4.7496 0.5860 -2.8480 O 0 0 0 0 0 0 0 0 0 0 0 0

2.0073 0.6737 -1.9582 H 0 0 0 0 0 0 0 0 0 0 0 0

2.3201 1.2313 -3.6233 H 0 0 0 0 0 0 0 0 0 0 0 0

0.7816 4.2646 0.8788 H 0 0 0 0 0 0 0 0 0 0 0 0

2.2405 4.7543 0.0698 H 0 0 0 0 0 0 0 0 0 0 0 0

1.6130 7.0789 0.1754 H 0 0 0 0 0 0 0 0 0 0 0 0

-1.0228 6.0896 1.3676 H 0 0 0 0 0 0 0 0 0 0 0 0

-0.3564 7.8226 1.2626 H 0 0 0 0 0 0 0 0 0 0 0 0

-0.3866 3.8273 -3.8072 H 0 0 0 0 0 0 0 0 0 0 0 0

0.3389 5.1882 -4.6720 H 0 0 0 0 0 0 0 0 0 0 0 0

-0.8654 5.4835 -3.4112 H 0 0 0 0 0 0 0 0 0 0 0 0

2.1718 6.6113 -1.6803 H 0 0 0 0 0 0 0 0 0 0 0 0

0.7945 7.1163 -2.6635 H 0 0 0 0 0 0 0 0 0 0 0 0

4.4164 3.7879 -3.0270 H 0 0 0 0 0 0 0 0 0 0 0 0

3.7742 4.7451 -1.7096 H 0 0 0 0 0 0 0 0 0 0 0 0

4.1223 5.4894 -4.6410 H 0 0 0 0 0 0 0 0 0 0 0 0

4.7005 6.3488 -3.1897 H 0 0 0 0 0 0 0 0 0 0 0 0

1 2 1 0 0 0 0

2 3 1 0 0 0 0

2 21 2 0 0 0 0

3 4 1 0 0 0 0

3 22 1 0 0 0 0

3 23 1 0 0 0 0

4 5 1 0 0 0 0

4 12 1 0 0 0 0

5 6 1 0 0 0 0

5 11 2 0 0 0 0

6 13 1 0 0 0 0

6 8 1 0 0 0 0

6 7 1 1 0 0 0

8 9 1 0 0 0 0

8 24 1 0 0 0 0

8 25 1 0 0 0 0

9 10 2 0 0 0 0

9 26 1 0 0 0 0

10 27 1 0 0 0 0

10 28 1 0 0 0 0

12 13 1 0 0 0 0

12 16 1 6 0 0 0

12 17 1 0 0 0 0

13 14 1 6 0 0 0

13 15 1 0 0 0 0

14 29 1 0 0 0 0

14 30 1 0 0 0 0

14 31 1 0 0 0 0

15 19 1 0 0 0 0

15 32 1 0 0 0 0

15 33 1 0 0 0 0

17 18 1 0 0 0 0

17 34 1 0 0 0 0

17 35 1 0 0 0 0

18 19 1 0 0 0 0

18 36 1 0 0 0 0

18 37 1 0 0 0 0

19 20 2 0 0 0 0

M END

**Compound (7).**

Compound identifier (MolFile):

O=C1C[C@@]([C@@H]2CC=C)(C)[C@@]3([H])C[C@]1([H])[C@H](O)CN3C2=O

OpenBabel05162213173D

37 39 0 0 1 0 0 0 0 0999 V2000

1.5198 0.1904 -0.3775 O 0 0 0 0 0 0 0 0 0 0 0 0

2.6401 0.0028 0.1011 C 0 0 0 0 0 0 0 0 0 0 0 0

3.8339 0.8041 -0.4181 C 0 0 0 0 0 0 0 0 0 0 0 0

5.0046 0.9827 0.5862 C 0 0 1 0 0 0 0 0 0 0 0 0

6.4199 0.9127 -0.0793 C 0 0 2 0 0 0 0 0 0 0 0 0

7.1942 1.2162 0.6396 H 0 0 0 0 0 0 0 0 0 0 0 0

6.6485 1.6506 -1.4039 C 0 0 0 0 0 0 0 0 0 0 0 0

6.4794 3.1388 -1.3164 C 0 0 0 0 0 0 0 0 0 0 0 0

5.6100 3.8358 -2.0592 C 0 0 0 0 0 0 0 0 0 0 0 0

4.8109 2.2591 1.4166 C 0 0 0 0 0 0 0 0 0 0 0 0

5.1202 -0.2486 1.4929 C 0 0 1 0 0 0 0 0 0 0 0 0

5.8783 -0.0655 2.2683 H 0 0 0 0 0 0 0 0 0 0 0 0

3.8322 -0.6505 2.1752 C 0 0 0 0 0 0 0 0 0 0 0 0

2.8692 -1.1195 1.1064 C 0 0 2 0 0 0 0 0 0 0 0 0

1.9083 -1.3351 1.5910 H 0 0 0 0 0 0 0 0 0 0 0 0

3.4102 -2.3974 0.4589 C 0 0 2 0 0 0 0 0 0 0 0 0

2.7606 -2.7030 -0.3693 H 0 0 0 0 0 0 0 0 0 0 0 0

3.3421 -3.4623 1.4197 O 0 0 0 0 0 0 0 0 0 0 0 0

4.8520 -2.2639 -0.0657 C 0 0 0 0 0 0 0 0 0 0 0 0

5.6811 -1.2481 0.5986 N 0 0 0 0 0 0 0 0 0 0 0 0

6.5760 -0.5941 -0.2842 C 0 0 0 0 0 0 0 0 0 0 0 0

7.2402 -1.1372 -1.1529 O 0 0 0 0 0 0 0 0 0 0 0 0

3.4663 1.7847 -0.7432 H 0 0 0 0 0 0 0 0 0 0 0 0

4.1607 0.2806 -1.3231 H 0 0 0 0 0 0 0 0 0 0 0 0

7.6768 1.4590 -1.7390 H 0 0 0 0 0 0 0 0 0 0 0 0

6.0100 1.2350 -2.1931 H 0 0 0 0 0 0 0 0 0 0 0 0

7.1261 3.6727 -0.6225 H 0 0 0 0 0 0 0 0 0 0 0 0

4.9562 3.3540 -2.7803 H 0 0 0 0 0 0 0 0 0 0 0 0

5.5393 4.9156 -1.9670 H 0 0 0 0 0 0 0 0 0 0 0 0

4.7801 3.1530 0.7885 H 0 0 0 0 0 0 0 0 0 0 0 0

5.6219 2.3903 2.1414 H 0 0 0 0 0 0 0 0 0 0 0 0

3.8688 2.2373 1.9739 H 0 0 0 0 0 0 0 0 0 0 0 0

3.4083 0.1865 2.7403 H 0 0 0 0 0 0 0 0 0 0 0 0

4.0228 -1.4469 2.9041 H 0 0 0 0 0 0 0 0 0 0 0 0

4.1671 -3.4689 1.9373 H 0 0 0 0 0 0 0 0 0 0 0 0

4.7936 -2.0084 -1.1317 H 0 0 0 0 0 0 0 0 0 0 0 0

5.3955 -3.2132 0.0025 H 0 0 0 0 0 0 0 0 0 0 0 0

1 2 2 0 0 0 0

2 3 1 0 0 0 0

2 14 1 0 0 0 0

3 4 1 0 0 0 0

3 23 1 0 0 0 0

3 24 1 0 0 0 0

4 5 1 0 0 0 0

4 10 1 1 0 0 0

4 11 1 0 0 0 0

5 21 1 0 0 0 0

5 7 1 0 0 0 0

5 6 1 1 0 0 0

7 8 1 0 0 0 0

7 25 1 0 0 0 0

7 26 1 0 0 0 0

8 9 2 0 0 0 0

8 27 1 0 0 0 0

9 28 1 0 0 0 0

9 29 1 0 0 0 0

10 30 1 0 0 0 0

10 31 1 0 0 0 0

10 32 1 0 0 0 0

11 12 1 1 0 0 0

11 13 1 0 0 0 0

11 20 1 0 0 0 0

13 14 1 0 0 0 0

13 33 1 0 0 0 0

13 34 1 0 0 0 0

14 15 1 1 0 0 0

14 16 1 0 0 0 0

16 19 1 0 0 0 0

16 18 1 0 0 0 0

16 17 1 6 0 0 0

18 35 1 0 0 0 0

19 20 1 0 0 0 0

19 36 1 0 0 0 0

19 37 1 0 0 0 0

20 21 1 0 0 0 0

21 22 2 0 0 0 0

M END

**Compound *epi*-7**

Compound identifier (MolFile):

O=C1C[C@@]([C@@H]2CC=C)(C)[C@@]3([H])C[C@]1([H])[C@@H](O)CN3C2=O

OpenBabel05162213203D

37 39 0 0 1 0 0 0 0 0999 V2000

1.3075 0.0563 0.1318 O 0 0 0 0 0 0 0 0 0 0 0 0

2.5130 -0.1061 0.3300 C 0 0 0 0 0 0 0 0 0 0 0 0

3.5396 0.8533 -0.2471 C 0 0 0 0 0 0 0 0 0 0 0 0

4.7753 1.0996 0.6619 C 0 0 1 0 0 0 0 0 0 0 0 0

6.0977 1.2164 -0.1700 C 0 0 2 0 0 0 0 0 0 0 0 0

6.9198 1.5544 0.4775 H 0 0 0 0 0 0 0 0 0 0 0 0

6.1019 2.0511 -1.4552 C 0 0 0 0 0 0 0 0 0 0 0 0

5.8429 3.5131 -1.2333 C 0 0 0 0 0 0 0 0 0 0 0 0

4.8294 4.1858 -1.7932 C 0 0 0 0 0 0 0 0 0 0 0 0

4.5380 2.3072 1.5773 C 0 0 0 0 0 0 0 0 0 0 0 0

5.1198 -0.1441 1.5087 C 0 0 1 0 0 0 0 0 0 0 0 0

5.8738 0.0997 2.2691 H 0 0 0 0 0 0 0 0 0 0 0 0

3.9289 -0.8197 2.1775 C 0 0 0 0 0 0 0 0 0 0 0 0

3.0114 -1.3321 1.0776 C 0 0 2 0 0 0 0 0 0 0 0 0

2.1347 -1.8072 1.5358 H 0 0 0 0 0 0 0 0 0 0 0 0

3.7201 -2.3662 0.1719 C 0 0 1 0 0 0 0 0 0 0 0 0

3.3923 -3.3734 0.4579 H 0 0 0 0 0 0 0 0 0 0 0 0

3.3450 -2.2050 -1.1942 O 0 0 0 0 0 0 0 0 0 0 0 0

5.2656 -2.3481 0.3093 C 0 0 0 0 0 0 0 0 0 0 0 0

5.7630 -0.9986 0.5359 N 0 0 0 0 0 0 0 0 0 0 0 0

6.3617 -0.2542 -0.4822 C 0 0 0 0 0 0 0 0 0 0 0 0

6.9475 -0.6933 -1.4563 O 0 0 0 0 0 0 0 0 0 0 0 0

3.0366 1.8016 -0.4697 H 0 0 0 0 0 0 0 0 0 0 0 0

3.8278 0.4213 -1.2113 H 0 0 0 0 0 0 0 0 0 0 0 0

7.0903 1.9642 -1.9265 H 0 0 0 0 0 0 0 0 0 0 0 0

5.4007 1.6404 -2.1913 H 0 0 0 0 0 0 0 0 0 0 0 0

6.5446 4.0495 -0.5975 H 0 0 0 0 0 0 0 0 0 0 0 0

4.1128 3.6999 -2.4490 H 0 0 0 0 0 0 0 0 0 0 0 0

4.6978 5.2480 -1.6102 H 0 0 0 0 0 0 0 0 0 0 0 0

4.3103 3.2128 1.0095 H 0 0 0 0 0 0 0 0 0 0 0 0

5.4174 2.5163 2.1962 H 0 0 0 0 0 0 0 0 0 0 0 0

3.6924 2.1343 2.2514 H 0 0 0 0 0 0 0 0 0 0 0 0

3.4046 -0.1162 2.8342 H 0 0 0 0 0 0 0 0 0 0 0 0

4.2621 -1.6539 2.8070 H 0 0 0 0 0 0 0 0 0 0 0 0

2.3807 -2.3332 -1.2659 H 0 0 0 0 0 0 0 0 0 0 0 0

5.7431 -2.7777 -0.5773 H 0 0 0 0 0 0 0 0 0 0 0 0

5.5641 -2.9368 1.1841 H 0 0 0 0 0 0 0 0 0 0 0 0

1 2 2 0 0 0 0

2 3 1 0 0 0 0

2 14 1 0 0 0 0

3 4 1 0 0 0 0

3 23 1 0 0 0 0

3 24 1 0 0 0 0

4 5 1 0 0 0 0

4 10 1 1 0 0 0

4 11 1 0 0 0 0

5 21 1 0 0 0 0

5 7 1 0 0 0 0

5 6 1 1 0 0 0

7 8 1 0 0 0 0

7 25 1 0 0 0 0

7 26 1 0 0 0 0

8 9 2 0 0 0 0

8 27 1 0 0 0 0

9 28 1 0 0 0 0

9 29 1 0 0 0 0

10 30 1 0 0 0 0

10 31 1 0 0 0 0

10 32 1 0 0 0 0

11 12 1 1 0 0 0

11 13 1 0 0 0 0

11 20 1 0 0 0 0

13 14 1 0 0 0 0

13 33 1 0 0 0 0

13 34 1 0 0 0 0

14 15 1 1 0 0 0

14 16 1 0 0 0 0

16 17 1 1 0 0 0

16 18 1 0 0 0 0

16 19 1 0 0 0 0

18 35 1 0 0 0 0

19 20 1 0 0 0 0

19 36 1 0 0 0 0

19 37 1 0 0 0 0

20 21 1 0 0 0 0

21 22 2 0 0 0 0

M END

**Compound 8.**

Compound identifier (MolFile):

O=C1[C@@H](CC=C)[C@@]2(C)CC([C@]3([H])C[C@@]2([H])N1C[C@H]3OS(C4=CC=C(C=C4)C)(=O)=O)=O

OpenBabel05162213223D

53 56 0 0 1 0 0 0 0 0999 V2000

1.8371 -1.5606 0.7701 O 0 0 0 0 0 0 0 0 0 0 0 0

2.5294 -0.6488 0.3492 C 0 0 0 0 0 0 0 0 0 0 0 0

2.1929 0.8265 0.2592 C 0 0 1 0 0 0 0 0 0 0 0 0

1.8004 0.9738 -0.7605 H 0 0 0 0 0 0 0 0 0 0 0 0

1.1500 1.3180 1.2506 C 0 0 0 0 0 0 0 0 0 0 0 0

-0.1966 0.6674 1.0652 C 0 0 0 0 0 0 0 0 0 0 0 0

-0.6517 -0.3267 1.8402 C 0 0 0 0 0 0 0 0 0 0 0 0

3.6056 1.4880 0.3425 C 0 0 1 0 0 0 0 0 0 0 0 0

3.6187 2.8708 -0.3337 C 0 0 0 0 0 0 0 0 0 0 0 0

4.3405 1.5701 1.7336 C 0 0 0 0 0 0 0 0 0 0 0 0

5.2443 0.3535 2.0804 C 0 0 0 0 0 0 0 0 0 0 0 0

6.2138 -0.1254 0.9887 C 0 0 2 0 0 0 0 0 0 0 0 0

7.2471 0.0925 1.2814 H 0 0 0 0 0 0 0 0 0 0 0 0

5.8271 0.6802 -0.2252 C 0 0 0 0 0 0 0 0 0 0 0 0

4.3698 0.4983 -0.5119 C 0 0 1 0 0 0 0 0 0 0 0 0

4.1667 0.6118 -1.5860 H 0 0 0 0 0 0 0 0 0 0 0 0

3.8267 -0.7897 -0.1092 N 0 0 0 0 0 0 0 0 0 0 0 0

4.6141 -1.9682 0.2649 C 0 0 0 0 0 0 0 0 0 0 0 0

6.0584 -1.6255 0.6644 C 0 0 1 0 0 0 0 0 0 0 0 0

6.2756 -2.1983 1.5812 H 0 0 0 0 0 0 0 0 0 0 0 0

7.0473 -2.0838 -0.2907 O 0 0 0 0 0 0 0 0 0 0 0 0

7.0081 -1.7182 -1.8905 S 0 0 0 0 0 0 0 0 0 0 0 0

8.2519 -2.6901 -2.6096 C 0 0 0 0 0 0 0 0 0 0 0 0

7.9006 -3.8345 -3.3018 C 0 0 0 0 0 0 0 0 0 0 0 0

8.9168 -4.5079 -3.9590 C 0 0 0 0 0 0 0 0 0 0 0 0

10.2350 -4.0121 -3.9333 C 0 0 0 0 0 0 0 0 0 0 0 0

10.5418 -2.8181 -3.2778 C 0 0 0 0 0 0 0 0 0 0 0 0

9.5457 -2.1563 -2.6154 C 0 0 0 0 0 0 0 0 0 0 0 0

11.3258 -4.7410 -4.6429 C 0 0 0 0 0 0 0 0 0 0 0 0

5.7449 -2.0494 -2.4699 O 0 0 0 0 0 0 0 0 0 0 0 0

7.6197 -0.3944 -2.1023 O 0 0 0 0 0 0 0 0 0 0 0 0

5.2237 -0.1665 3.1963 O 0 0 0 0 0 0 0 0 0 0 0 0

1.4967 1.1749 2.2783 H 0 0 0 0 0 0 0 0 0 0 0 0

1.0090 2.3963 1.1095 H 0 0 0 0 0 0 0 0 0 0 0 0

-0.8194 1.0170 0.2453 H 0 0 0 0 0 0 0 0 0 0 0 0

-0.0420 -0.7521 2.6337 H 0 0 0 0 0 0 0 0 0 0 0 0

-1.6143 -0.7915 1.6551 H 0 0 0 0 0 0 0 0 0 0 0 0

3.0667 3.5991 0.2636 H 0 0 0 0 0 0 0 0 0 0 0 0

3.1726 2.8312 -1.3343 H 0 0 0 0 0 0 0 0 0 0 0 0

4.6387 3.2541 -0.4652 H 0 0 0 0 0 0 0 0 0 0 0 0

3.6490 1.7658 2.5517 H 0 0 0 0 0 0 0 0 0 0 0 0

5.0474 2.4160 1.7180 H 0 0 0 0 0 0 0 0 0 0 0 0

6.2164 0.3290 -1.1239 H 0 0 0 0 0 0 0 0 0 0 0 0

6.2108 1.7036 -0.2012 H 0 0 0 0 0 0 0 0 0 0 0 0

4.5473 -2.7147 -0.5285 H 0 0 0 0 0 0 0 0 0 0 0 0

4.1153 -2.4283 1.1319 H 0 0 0 0 0 0 0 0 0 0 0 0

6.8711 -4.1775 -3.3390 H 0 0 0 0 0 0 0 0 0 0 0 0

8.6745 -5.4221 -4.4908 H 0 0 0 0 0 0 0 0 0 0 0 0

11.5455 -2.4027 -3.2723 H 0 0 0 0 0 0 0 0 0 0 0 0

9.7672 -1.1981 -2.1373 H 0 0 0 0 0 0 0 0 0 0 0 0

11.2779 -5.8043 -4.3973 H 0 0 0 0 0 0 0 0 0 0 0 0

12.3052 -4.3677 -4.3262 H 0 0 0 0 0 0 0 0 0 0 0 0

11.2243 -4.5929 -5.7201 H 0 0 0 0 0 0 0 0 0 0 0 0

1 2 2 0 0 0 0

2 3 1 0 0 0 0

2 17 1 0 0 0 0

3 8 1 0 0 0 0

3 4 1 6 0 0 0

3 5 1 0 0 0 0

5 6 1 0 0 0 0

5 33 1 0 0 0 0

5 34 1 0 0 0 0

6 7 2 0 0 0 0

6 35 1 0 0 0 0

7 36 1 0 0 0 0

7 37 1 0 0 0 0

8 10 1 0 0 0 0

8 15 1 0 0 0 0

8 9 1 6 0 0 0

9 38 1 0 0 0 0

9 39 1 0 0 0 0

9 40 1 0 0 0 0

10 11 1 0 0 0 0

10 41 1 0 0 0 0

10 42 1 0 0 0 0

11 12 1 0 0 0 0

11 32 2 0 0 0 0

12 14 1 0 0 0 0

12 19 1 0 0 0 0

12 13 1 1 0 0 0

14 15 1 0 0 0 0

14 43 1 0 0 0 0

14 44 1 0 0 0 0

15 17 1 0 0 0 0

15 16 1 6 0 0 0

17 18 1 0 0 0 0

18 19 1 0 0 0 0

18 45 1 0 0 0 0

18 46 1 0 0 0 0

19 21 1 0 0 0 0

19 20 1 1 0 0 0

21 22 1 0 0 0 0

22 23 1 0 0 0 0

22 30 2 0 0 0 0

22 31 2 0 0 0 0

23 24 2 0 0 0 0

23 28 1 0 0 0 0

24 25 1 0 0 0 0

24 47 1 0 0 0 0

25 26 2 0 0 0 0

25 48 1 0 0 0 0

26 27 1 0 0 0 0

26 29 1 0 0 0 0

27 28 2 0 0 0 0

27 49 1 0 0 0 0

28 50 1 0 0 0 0

29 51 1 0 0 0 0

29 52 1 0 0 0 0

29 53 1 0 0 0 0

M END

**Compound (9).**

Compound identifier (MolFile):

O=C1[C@@H](CC=C)[C@@]2(C)CC([C@]3([H])C[C@@]2([H])N1C[C@H]3C)=O

39 41 0 0 1 0 0 0 0 0999 V2000

5.6911 2.0847 0.6884 O 0 0 0 0 0 0 0 0 0 0 0 0

4.5338 2.4283 0.5062 C 0 0 0 0 0 0 0 0 0 0 0 0

3.2971 1.6172 0.8770 C 0 0 1 0 0 0 0 0 0 0 0 0

3.4966 1.0426 1.7902 H 0 0 0 0 0 0 0 0 0 0 0 0

2.9085 0.6801 -0.2747 C 0 0 0 0 0 0 0 0 0 0 0 0

3.8613 -0.4783 -0.4036 C 0 0 0 0 0 0 0 0 0 0 0 0

3.5350 -1.7537 -0.1599 C 0 0 0 0 0 0 0 0 0 0 0 0

2.2992 2.7862 1.1893 C 0 0 1 0 0 0 0 0 0 0 0 0

0.8218 2.3876 1.1162 C 0 0 0 0 0 0 0 0 0 0 0 0

2.6124 3.3466 2.6134 C 0 0 0 0 0 0 0 0 0 0 0 0

2.4877 4.8561 2.7476 C 0 0 0 0 0 0 0 0 0 0 0 0

2.9727 5.7185 1.5905 C 0 0 2 0 0 0 0 0 0 0 0 0

2.6636 6.7491 1.8045 H 0 0 0 0 0 0 0 0 0 0 0 0

2.2221 5.2211 0.3603 C 0 0 0 0 0 0 0 0 0 0 0 0

2.6637 3.7904 0.0812 C 0 0 1 0 0 0 0 0 0 0 0 0

2.2683 3.4716 -0.8911 H 0 0 0 0 0 0 0 0 0 0 0 0

4.1043 3.6655 0.0198 N 0 0 0 0 0 0 0 0 0 0 0 0

4.9275 4.8535 0.1562 C 0 0 0 0 0 0 0 0 0 0 0 0

4.5191 5.6241 1.4311 C 0 0 1 0 0 0 0 0 0 0 0 0

4.9361 5.0899 2.2955 H 0 0 0 0 0 0 0 0 0 0 0 0

5.1530 7.0154 1.4232 C 0 0 0 0 0 0 0 0 0 0 0 0

2.0819 5.3668 3.7916 O 0 0 0 0 0 0 0 0 0 0 0 0

1.8981 0.2888 -0.1116 H 0 0 0 0 0 0 0 0 0 0 0 0

2.8936 1.2110 -1.2339 H 0 0 0 0 0 0 0 0 0 0 0 0

4.8797 -0.2501 -0.7172 H 0 0 0 0 0 0 0 0 0 0 0 0

2.5382 -2.0403 0.1590 H 0 0 0 0 0 0 0 0 0 0 0 0

4.2737 -2.5426 -0.2700 H 0 0 0 0 0 0 0 0 0 0 0 0

0.6153 1.5327 1.7693 H 0 0 0 0 0 0 0 0 0 0 0 0

0.5205 2.1158 0.0992 H 0 0 0 0 0 0 0 0 0 0 0 0

0.1733 3.2119 1.4330 H 0 0 0 0 0 0 0 0 0 0 0 0

3.6353 3.1036 2.9261 H 0 0 0 0 0 0 0 0 0 0 0 0

1.9509 2.8796 3.3534 H 0 0 0 0 0 0 0 0 0 0 0 0

2.4463 5.8670 -0.4974 H 0 0 0 0 0 0 0 0 0 0 0 0

1.1374 5.2649 0.5139 H 0 0 0 0 0 0 0 0 0 0 0 0

4.7787 5.4632 -0.7426 H 0 0 0 0 0 0 0 0 0 0 0 0

5.9862 4.5759 0.1996 H 0 0 0 0 0 0 0 0 0 0 0 0

4.7775 7.6185 0.5897 H 0 0 0 0 0 0 0 0 0 0 0 0

6.2418 6.9453 1.3287 H 0 0 0 0 0 0 0 0 0 0 0 0

4.9319 7.5467 2.3549 H 0 0 0 0 0 0 0 0 0 0 0 0

1 2 2 0 0 0 0

2 3 1 0 0 0 0

2 17 1 0 0 0 0

3 8 1 0 0 0 0

3 4 1 6 0 0 0

3 5 1 0 0 0 0

5 6 1 0 0 0 0

5 23 1 0 0 0 0

5 24 1 0 0 0 0

6 7 2 0 0 0 0

6 25 1 0 0 0 0

7 26 1 0 0 0 0

7 27 1 0 0 0 0

8 10 1 0 0 0 0

8 15 1 0 0 0 0

8 9 1 6 0 0 0

9 28 1 0 0 0 0

9 29 1 0 0 0 0

9 30 1 0 0 0 0

10 11 1 0 0 0 0

10 31 1 0 0 0 0

10 32 1 0 0 0 0

11 12 1 0 0 0 0

11 22 2 0 0 0 0

12 14 1 0 0 0 0

12 19 1 0 0 0 0

12 13 1 1 0 0 0

14 15 1 0 0 0 0

14 33 1 0 0 0 0

14 34 1 0 0 0 0

15 17 1 0 0 0 0

15 16 1 6 0 0 0

17 18 1 0 0 0 0

18 19 1 0 0 0 0

18 35 1 0 0 0 0

18 36 1 0 0 0 0

19 21 1 0 0 0 0

19 20 1 1 0 0 0

21 37 1 0 0 0 0

21 38 1 0 0 0 0

21 39 1 0 0 0 0

M END

**Compound (10)**

Compound identifier (MolFile):

O=C1[C@@H](CC=C)[C@@]2(C)C[C@]3(C)[C@]4([H])C[C@@]2([H])N1C[C@@H]4O3

OpenBabel05162213353D

39 42 0 0 1 0 0 0 0 0999 V2000

5.3785 2.0021 1.2641 O 0 0 0 0 0 0 0 0 0 0 0 0

4.3232 2.2886 0.7206 C 0 0 0 0 0 0 0 0 0 0 0 0

3.0622 1.4480 0.7516 C 0 0 1 0 0 0 0 0 0 0 0 0

2.9720 0.9687 1.7333 H 0 0 0 0 0 0 0 0 0 0 0 0

3.1208 0.3815 -0.3574 C 0 0 0 0 0 0 0 0 0 0 0 0

4.1997 -0.6451 -0.1449 C 0 0 0 0 0 0 0 0 0 0 0 0

5.1750 -0.8958 -1.0277 C 0 0 0 0 0 0 0 0 0 0 0 0

1.9566 2.5486 0.5729 C 0 0 1 0 0 0 0 0 0 0 0 0

0.6698 1.9920 -0.0686 C 0 0 0 0 0 0 0 0 0 0 0 0

1.5593 3.2047 1.9434 C 0 0 0 0 0 0 0 0 0 0 0 0

2.1574 4.5935 2.2449 C 0 0 2 0 0 0 0 0 0 0 0 0

1.4411 5.2750 3.4104 C 0 0 0 0 0 0 0 0 0 0 0 0

2.4197 5.5309 1.0460 C 0 0 2 0 0 0 0 0 0 0 0 0

2.0419 6.5524 1.1682 H 0 0 0 0 0 0 0 0 0 0 0 0

2.0746 4.9489 -0.3132 C 0 0 0 0 0 0 0 0 0 0 0 0

2.6493 3.5358 -0.3850 C 0 0 1 0 0 0 0 0 0 0 0 0

2.5969 3.1783 -1.4208 H 0 0 0 0 0 0 0 0 0 0 0 0

4.0495 3.4620 0.0063 N 0 0 0 0 0 0 0 0 0 0 0 0

4.7723 4.6701 0.3788 C 0 0 0 0 0 0 0 0 0 0 0 0

3.9095 5.3819 1.4265 C 0 0 2 0 0 0 0 0 0 0 0 0

4.3708 6.3079 1.7884 H 0 0 0 0 0 0 0 0 0 0 0 0

3.5720 4.5110 2.5426 O 0 0 0 0 0 0 0 0 0 0 0 0

2.1732 -0.1691 -0.3841 H 0 0 0 0 0 0 0 0 0 0 0 0

3.2436 0.8537 -1.3400 H 0 0 0 0 0 0 0 0 0 0 0 0

4.1800 -1.2024 0.7898 H 0 0 0 0 0 0 0 0 0 0 0 0

5.2498 -0.3584 -1.9678 H 0 0 0 0 0 0 0 0 0 0 0 0

5.9371 -1.6391 -0.8143 H 0 0 0 0 0 0 0 0 0 0 0 0

0.2474 1.1868 0.5430 H 0 0 0 0 0 0 0 0 0 0 0 0

0.8440 1.5995 -1.0758 H 0 0 0 0 0 0 0 0 0 0 0 0

-0.0942 2.7716 -0.1683 H 0 0 0 0 0 0 0 0 0 0 0 0

1.7803 2.5209 2.7728 H 0 0 0 0 0 0 0 0 0 0 0 0

0.4665 3.3207 1.9622 H 0 0 0 0 0 0 0 0 0 0 0 0

0.3993 5.4936 3.1525 H 0 0 0 0 0 0 0 0 0 0 0 0

1.9241 6.2210 3.6790 H 0 0 0 0 0 0 0 0 0 0 0 0

1.4451 4.6361 4.2999 H 0 0 0 0 0 0 0 0 0 0 0 0

2.5078 5.5744 -1.1030 H 0 0 0 0 0 0 0 0 0 0 0 0

0.9907 4.9424 -0.4709 H 0 0 0 0 0 0 0 0 0 0 0 0

4.9006 5.2887 -0.5149 H 0 0 0 0 0 0 0 0 0 0 0 0

5.7595 4.4259 0.7829 H 0 0 0 0 0 0 0 0 0 0 0 0

1 2 2 0 0 0 0

2 3 1 0 0 0 0

2 18 1 0 0 0 0

3 8 1 0 0 0 0

3 4 1 6 0 0 0

3 5 1 0 0 0 0

5 6 1 0 0 0 0

5 23 1 0 0 0 0

5 24 1 0 0 0 0

6 7 2 0 0 0 0

6 25 1 0 0 0 0

7 26 1 0 0 0 0

7 27 1 0 0 0 0

8 10 1 0 0 0 0

8 16 1 0 0 0 0

8 9 1 6 0 0 0

9 28 1 0 0 0 0

9 29 1 0 0 0 0

9 30 1 0 0 0 0

10 11 1 0 0 0 0

10 31 1 0 0 0 0

10 32 1 0 0 0 0

11 13 1 0 0 0 0

11 22 1 0 0 0 0

11 12 1 1 0 0 0

12 33 1 0 0 0 0

12 34 1 0 0 0 0

12 35 1 0 0 0 0

13 15 1 0 0 0 0

13 20 1 0 0 0 0

13 14 1 1 0 0 0

15 16 1 0 0 0 0

15 36 1 0 0 0 0

15 37 1 0 0 0 0

16 18 1 0 0 0 0

16 17 1 6 0 0 0

18 19 1 0 0 0 0

19 20 1 0 0 0 0

19 38 1 0 0 0 0

19 39 1 0 0 0 0

20 22 1 0 0 0 0

20 21 1 1 0 0 0

M END
