# Supplementary material for: Synthesis of the ABC Ring of Calyciphylline A-Type Alkaloids by a Stereocontrolled Aldol Cyclization: Formal Synthesis of (±)-Himalensine A
Source: J Org Chem. 2022 Jul 21;87(15):10516–22. doi: 10.1021/acs.joc.2c01171 (PMC9881646; doi:10.1021/acs.joc.2c01171)

# Synthesis of the ABC Ring of Calyciphylline A Type Alkaloids by a Stereocontrolled Aldol Cyclization: Formal Synthesis of (±)-Himalensine A

Clàudia Marquès, Faïza Diaba*, Enrique Gómez-Bengoa, and Josep Bonjoch*

Laboratori de Química Orgànica, Facultat de Farmàcia, IBUB,
Universitat de Barcelona, Av. Joan XXIII s/n, 08028-Barcelona, Spain
Departamento de Química Orgánica I, Universidad del País Vasco, Manuel Lardizábal 3, 20018 San Sebastián, Spain

josep.bonjoch@ub.edu; faiza.diaba@ub.edu

**Contents**

A chromatography-free, four-step sequence for the transformation of enelactam **3** to azatricycle **7** **S2**

NMR data of by-product compounds **4'** and **10** **S3**

Experimental for dehydration reactions of **3'** and **4'** **S3**

Comparison of $^{13}C$ NMR data for Azatricycle **9** **S4**

DFT calculations for the diastereoselectivity in the aldol cyclization of **6** **S5**

X-ray crystallographic data of tosylate **8** **S10**

Copies of $^{1}H$- and $^{13}C$-NMR spectra of compounds **2–10** **S13**

A chromatography-free, four-step sequence for the transformation of enelactam **3** to azatricycle **7**

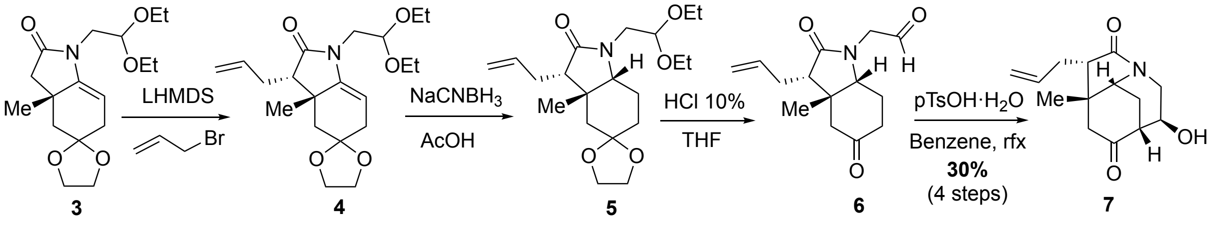


Lactam **3** (1.71 g, 5.24 mmol) in THF (17 mL) was cooled to -78 °C and a solution of LHMDS in THF (1 M, 6.8 mL, 6.8 mmol) was added dropwise. After being stirred for 30 min, allylbromide (0.96 mL, 10.5 mmol) was added and the reaction was left to reach room temperature over 2 h. It was then quenched with $NH_4Cl$ sat. solution (30 mL) and extracted with $Et_2O$ (3 x 30 mL). The organics were dried and concentrated. The obtained crude (1.84) was diluted in AcOH (8.5 mL) and $NaCNBH_3$ (0.63 g, 10.1 mmol) was added portionwise. The stirring was prolonged for 2h 30 min before quenching with 15% NaOH and extracted with EtOAc (3 x 20 mL). The combined organic extracts were dried over $Na_2SO_4$, filtered and concentrated and the residue (1.52 g) was taken up with a solution 10% HCl:THF solution (90 mL, 1:3) and stirred overnight at room temperature. $Na_2CO_3$ sat. solution (70 mL) was added and the mixture was extracted with $CH_2Cl_2$ (4 x 80 mL). Organic phases were combined, washed with brine (50 mL), dried, filtered and concentrated under vacuum. A solution of the residue (0.99 g) and pTsOH·$H_2O$ (0.75 g, 3.97 mmol) in benzene (70 mL) was heated to reflux for 15 min. After being cooled, water was added and extracted with $CH_2Cl_2$ (3 x 30mL) and $CHCl_3$:i-PrOH (4:1, 2 x 30 mL). The combined organic layers were concentrated and purification was carried out by chromatography ($CH_2Cl_2$:MeOH, 1:0 → 9.5:0.5) to obtain **7** as a solid (392 mg, 30% over 4 steps).

## NMR data of compounds **4'** and **10**

Compound **4':** $^{1}H$ NMR (400 MHz, $CDCl_3$) δ 6.00-5.91, 5.11, 5.02, 4.61, 3.99-3.95, 3.91-3.87, 3.83-3.77, 3.74-3.65, 3.50, 2.91, 2.68, 2.63, 2.09, 1.78, 1.63-1.51, 1.47, 1.31, 1.25, 1.22; $^{13}C$ NMR (101 MHz, $CDCl_3$) δ 177.8, 137.6, 115.7, 107.7, 100.0, 89.3, 64.6, 64.4, 63.8, 63.6, 50.8, 45.4, 42.3, 39.5, 30.8, 28.3, 28.0, 18.8, 15.3, 14.7.

Compound **10:** IR (NaCl): 2926, 1701, 1393, 1349, 1302, 972 $cm^{-1}$; $^{1}H$ NMR (400 MHz, $CDCl_3$) δ 6.05-5.95 (m, 1H, =CH), 5.13 (d, *J* = 17.0 Hz, 1H, $=CH_2$ cis), 5.02 (d, *J* = 10.0 Hz, 1H, $CH_2$ trans), 4.67 (dd, *J* = 8.0, 3.0 Hz, 1H, H-8), 4.14 (dd, *J* = 14.4, 3.0 Hz, 1H, H-9), 3.60 (d, *J* = 5.2 Hz, 1H, H-7a), 3.06 (d, *J* = 14.4 Hz, 1H, H-9), 2.77-2.70 (m, 1H, $CH_2$-3), 2.64-2.60 (m, 1H, H-6), 2.52 (dd, *J* = 8.0, 6.4 Hz, 1H, H-3), 2.22 (m, 1H, $CH_2$-3), 2.07 (d, *J* = 16.4 Hz, 1H, H-4), 1.97 (ddd, *J* = 14.8, 3.4, 2.6 Hz, 1H, H-10), 1.69 (ddd, *J* = 14.8, 3.4, 1.2 Hz, 1H, H-10), 1.39 (s, 3H, Me-5), 1.17 (s, 3H, Me-3a), 1.10 (d, *J* = 16.4 Hz, 1H, H-10); $^{13}C$ NMR (101 MHz, $CDCl_3$) δ 182.7 (C-2), 137.6 (=CH), 115.6 ($CH_2$), 82.5 (C-5), 74.2 (C-8), 59.2 (C-7a), 52.9 (C-3), 44.3 (C-3a), 43.8 (C-9), 39.6 (C-4), 34.1 (C-6), 30.5 ($CH_2$-3), 29.9 (Me-5), 27.6 (Me-3a), 18.0 (C-10); HRMS (ESI-TOF) *m/z*: $[M+H]^+$ calcd for $C_{15}H_{22}NO_2$ 248.1645, found 248.1649.

## Experimental for dehydration reactions:

Treatment of the mixture with camphorsulphonic acid (0.04 equiv) and molecular sieves (5:1 in weight) in $CH_2Cl_2$ (0.04 M) and heating to reflux for 4 h reverted the process and product was recovered with almost quantitative yield.

**Table S1. Comparison of $^{13}$C NMR data for azatricycle 9 (this work and Gao`s work)**

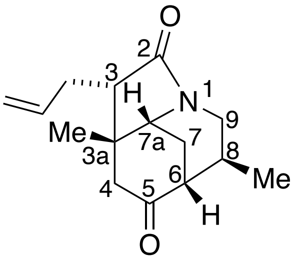


**9**

| carbon | Synthetic **9**[1] | Gao´s Compound[2] |
|---|---|---|
| | δ $^{13}$C | δ $^{13}$C |
| 2 | 174.2 | 174.3 |
| 3 | 50.4 | 50.5 |
| 3a | 47.3 | 47.4 |
| 4 | 44.4 | 44.4 |
| 5 | 213.0 | 213.1 |
| 6 | 46.0 | 46.0 |
| 7 | 19.6 | 19.6 |
| 7a | 60.2 | 60.3 |
| 8 | 36.6 | 36.6 |
| 9 | 40.6 | 40.6 |
| 3a-Me | 24.4 | 24.5 |
| 8-Me | 17.8 | 17.8 |
| =$CH_2$ | 116.3 | 116.3 |
| =CH | 136.7 | 136.7 |
| 3-$CH_2$ | 30.0 | 30.0 |

[1] Recorded at 101 MHz. Assignments were aided by gCOSY and gHSQCAD spectra.
[2] Recorded at 126 MHz (*Org. Lett.,* 2019, **21**, 3741-3745).

## DFT calculations of the two modes for the aldol cyclization of 6

All structures were optimized using density functional theory (DFT) as implemented in Gaussian 16,[1] with M06-2X[2] as functional, and 6-311++G(d,p) as basis set, introducing solvation factors with the IEF-PCM[3] method, and benzene as solvent, as in the optimized experimental conditions. The stationary points were characterized by frequency calculations in order to verify that they have the right number of imaginary frequencies.

**Table S2**. Energies of the structures included in the main manuscript

| Compound | G (M06-2X) (kcal/mol) | ΔG (kcal/mol) | Negative Frequency ($cm^{-1}$) |
|---|---|---|---|
| **7** | -824.978625 | 0 | |
| *epi*-**7** | -824.978309 | 0.1 | |
| **6**-*p*TsOH | -1720.155492 | 0 | |
| **TS1** | -1720.138304 | 10.8 | -290.4 |
| *epi*-**TS1** | -1720.134628 | 13.1 | -290.9 |
| **7**-*p*TsOH | -1720.174573 | -12.0 | |
| *epi*-**7**-*p*TsOH | -1720.176310 | -13.1 | |

Cartesian Coordinates of the optimized structures are shown below:

[1] Gaussian 16, Revision C.01, Frisch, M. J.; Trucks, G. W.; Schlegel, H. B.; Scuseria, G. E.; Robb, M. A.; Cheeseman, J. R.; Scalmani, G.; Barone, V.; Petersson, G. A.; Nakatsuji, H.; Li, X.; Caricato, M.; Marenich, A. V.; Bloino, J.; Janesko, B. G.; Gomperts, R.; Mennucci, B.; Hratchian, H. P.; Ortiz, J. V.; Izmaylov, A. F.; Sonnenberg, J. L.; Williams-Young, D.; Ding, F.; Lipparini, F.; Egidi, F.; Goings, J.; Peng, B.; Petrone, A.; Henderson, T.; Ranasinghe, D.; Zakrzewski, V. G.; Gao, J.; Rega, N.; Zheng, G.; Liang, W.; Hada, M.; Ehara, M.; Toyota, K.; Fukuda, R.; Hasegawa, J.; Ishida, M.; Nakajima, T.; Honda, Y.; Kitao, O.; Nakai, H.; Vreven, T.; Throssell, K.; Montgomery, J. A., Jr.; Peralta, J. E.; Ogliaro, F.; Bearpark, M. J.; Heyd, J. J.; Brothers, E. N.; Kudin, K. N.; Staroverov, V. N.; Keith, T. A.; Kobayashi, R.; Normand, J.; Raghavachari, K.; Rendell, A. P.; Burant, J. C.; Iyengar, S. S.; Tomasi, J.; Cossi, M.; Millam, J. M.; Klene, M.; Adamo, C.; Cammi, R.; Ochterski, J. W.; Martin, R. L.; Morokuma, K.; Farkas, O.; Foresman, J. B.; Fox, D. J. Gaussian, Inc., Wallingford CT, **2016**.

[2] Zhao, Y.; Truhlar, D. G. *Theor. Chem. Acc.*, **2008**, *120*, 215-41

[3] (a) Cancés, E.; Mennucci, B.; Tomasi, J. *J. Chem. Phys.*, **1997**, *107*, 3032-3041; (b) Cossi, M.; Barone, V.; Mennucci, B.; Tomasi, J. *Chem. Phys. Lett.*, **1998**, *286*, 253-260; (c) Tomasi, J.; Mennucci, B.; Cancès, E. *J. Mol.Struc.: THEOCHEM*, **1999**, *464*, 211-226.

## 7

Standard orientation:

| Center Number | Atomic Number | Atomic Type | Coordinates (Angstroms) X | Y | Z |
|---|---|---|---|---|---|
| 1 | 6 | 0 | -0.518937 | -0.697284 | -1.281137 |
| 2 | 6 | 0 | -2.158031 | 1.171824 | -0.867731 |
| 3 | 6 | 0 | -2.057863 | -0.865139 | 0.613091 |
| 4 | 6 | 0 | -2.787314 | 0.462507 | 0.359878 |
| 5 | 1 | 0 | -2.754807 | 0.928780 | -1.751740 |
| 6 | 1 | 0 | -2.133307 | 2.254894 | -0.744977 |
| 7 | 1 | 0 | -2.725908 | -1.494681 | 1.207736 |
| 8 | 7 | 0 | -0.795155 | 0.721379 | -1.053891 |
| 9 | 6 | 0 | 0.287896 | 1.428138 | -0.612739 |
| 10 | 6 | 0 | 1.475774 | 0.467444 | -0.577724 |
| 11 | 6 | 0 | 0.809609 | -0.931571 | -0.506199 |
| 12 | 6 | 0 | -0.794864 | -0.630382 | 1.452665 |
| 13 | 6 | 0 | 0.454188 | -1.329936 | 0.949769 |
| 14 | 1 | 0 | 0.246949 | -2.406234 | 0.967685 |
| 15 | 1 | 0 | 1.272493 | -1.140556 | 1.643140 |
| 16 | 8 | 0 | 0.288463 | 2.594150 | -0.290086 |
| 17 | 6 | 0 | 2.464236 | 0.895415 | 0.512708 |
| 18 | 1 | 0 | 2.762818 | 1.921471 | 0.286726 |
| 19 | 1 | 0 | 1.943297 | 0.934534 | 1.474891 |
| 20 | 6 | 0 | 3.678571 | 0.016299 | 0.604003 |
| 21 | 1 | 0 | 3.548243 | -0.964073 | 1.058554 |
| 22 | 6 | 0 | 4.885258 | 0.361339 | 0.168567 |
| 23 | 1 | 0 | 5.063105 | 1.334262 | -0.279330 |
| 24 | 1 | 0 | 5.732875 | -0.308645 | 0.250498 |
| 25 | 6 | 0 | -1.688135 | -1.515161 | -0.723274 |
| 26 | 1 | 0 | -1.396705 | -2.562242 | -0.614948 |
| 27 | 1 | 0 | -2.548143 | -1.490289 | -1.395970 |
| 28 | 1 | 0 | 1.980964 | 0.544802 | -1.549585 |
| 29 | 8 | 0 | -0.806875 | 0.051488 | 2.445869 |
| 30 | 1 | 0 | -0.368739 | -0.892420 | -2.349928 |
| 31 | 6 | 0 | 1.637232 | -2.033831 | -1.161415 |
| 32 | 1 | 0 | 1.954092 | -1.736930 | -2.163980 |
| 33 | 1 | 0 | 2.531960 | -2.260309 | -0.579497 |
| 34 | 1 | 0 | 1.049431 | -2.953221 | -1.247394 |
| 35 | 8 | 0 | -4.151866 | 0.153902 | 0.117249 |
| 36 | 1 | 0 | -4.667008 | 0.964056 | 0.143417 |
| 37 | 1 | 0 | -2.689021 | 1.082852 | 1.254891 |

## *epi*-7

Standard orientation:

| Center Number | Atomic Number | Atomic Type | Coordinates (Angstroms) X | Y | Z |
|---|---|---|---|---|---|
| 1 | 6 | 0 | -0.438694 | 1.052978 | 1.177204 |
| 2 | 6 | 0 | -2.207213 | -0.759650 | 1.261195 |
| 3 | 6 | 0 | -2.120091 | 0.947163 | -0.600496 |
| 4 | 6 | 0 | -2.982375 | -0.161350 | 0.047158 |
| 5 | 1 | 0 | -2.668290 | -0.447588 | 2.202633 |
| 6 | 1 | 0 | -2.236300 | -1.848026 | 1.207983 |
| 7 | 1 | 0 | -2.741950 | 1.502657 | -1.309064 |
| 8 | 1 | 0 | -3.888673 | 0.319110 | 0.426932 |
| 9 | 8 | 0 | -3.428901 | -1.133777 | -0.858605 |
| 10 | 1 | 0 | -2.697810 | -1.368551 | -1.445843 |
| 11 | 7 | 0 | -0.814307 | -0.358083 | 1.242390 |
| 12 | 6 | 0 | 0.210922 | -1.209387 | 0.936328 |
| 13 | 6 | 0 | 1.442168 | -0.351873 | 0.645013 |
| 14 | 6 | 0 | 0.839273 | 1.031911 | 0.293711 |
| 15 | 6 | 0 | -0.937570 | 0.385179 | -1.397668 |
| 16 | 6 | 0 | 0.383636 | 1.098563 | -1.188010 |
| 17 | 1 | 0 | 0.232137 | 2.150704 | -1.455058 |
| 18 | 1 | 0 | 1.124837 | 0.684967 | -1.870421 |
| 19 | 8 | 0 | 0.140477 | -2.414750 | 0.879473 |
| 20 | 6 | 0 | 2.352899 | -1.065808 | -0.360004 |
| 21 | 1 | 0 | 2.630056 | -2.023103 | 0.086815 |
| 22 | 1 | 0 | 1.776880 | -1.306695 | -1.259192 |
| 23 | 6 | 0 | 3.587658 | -0.287850 | -0.715063 |
| 24 | 1 | 0 | 3.464935 | 0.554377 | -1.393679 |
| 25 | 6 | 0 | 4.803148 | -0.564986 | -0.256609 |
| 26 | 1 | 0 | 4.973001 | -1.404280 | 0.410888 |
| 27 | 1 | 0 | 5.665961 | 0.027675 | -0.536430 |
| 28 | 6 | 0 | -1.587726 | 1.835454 | 0.531896 |
| 29 | 1 | 0 | -1.231423 | 2.805729 | 0.179111 |
| 30 | 1 | 0 | -2.381962 | 2.033151 | 1.256407 |
| 31 | 1 | 0 | 1.990945 | -0.241479 | 1.589788 |
| 32 | 8 | 0 | -1.049756 | -0.554710 | -2.151648 |
| 33 | 1 | 0 | -0.200667 | 1.441543 | 2.174909 |
| 34 | 6 | 0 | 1.757959 | 2.205051 | 0.620060 |
| 35 | 1 | 0 | 2.138375 | 2.126127 | 1.641180 |
| 36 | 1 | 0 | 2.614225 | 2.241309 | -0.054840 |
| 37 | 1 | 0 | 1.217348 | 3.152483 | 0.529098 |

**6**-*p*TsOH

Standard orientation:

---------------------------------------------------------------------

| Center Number | Atomic Number | Atomic Type | Coordinates (Angstroms) X | Y | Z |
|---|---|---|---|---|---|
| 1 | 6 | 0 | -3.075042 | 0.844624 | 1.255282 |
| 2 | 6 | 0 | -1.984260 | 2.664430 | -0.214244 |
| 3 | 6 | 0 | -0.867917 | -0.374445 | 1.357238 |
| 4 | 6 | 0 | -0.648547 | 2.056398 | -0.565081 |
| 5 | 1 | 0 | -1.873458 | 3.340545 | 0.636412 |
| 6 | 1 | 0 | -2.319364 | 3.240975 | -1.084756 |
| 7 | 1 | 0 | 0.166178 | -0.412575 | 1.682800 |
| 8 | 7 | 0 | -2.957045 | 1.632707 | 0.021343 |
| 9 | 6 | 0 | -3.953394 | 1.333721 | -0.863095 |
| 10 | 6 | 0 | -4.766919 | 0.185629 | -0.259471 |
| 11 | 6 | 0 | -3.742426 | -0.441125 | 0.718500 |
| 12 | 6 | 0 | -1.311298 | -1.234001 | 0.435385 |
| 13 | 6 | 0 | -2.717506 | -1.270031 | -0.084049 |
| 14 | 1 | 0 | -3.041064 | -2.315261 | -0.129659 |
| 15 | 1 | 0 | -2.665070 | -0.934299 | -1.126852 |
| 16 | 8 | 0 | -4.134378 | 1.880291 | -1.932780 |
| 17 | 6 | 0 | -5.418083 | -0.666083 | -1.353650 |
| 18 | 1 | 0 | -6.018495 | 0.007958 | -1.969331 |
| 19 | 1 | 0 | -4.644709 | -1.077777 | -2.008446 |
| 20 | 6 | 0 | -6.281560 | -1.776886 | -0.825083 |
| 21 | 1 | 0 | -5.771541 | -2.665521 | -0.460266 |
| 22 | 6 | 0 | -7.609100 | -1.735026 | -0.787301 |
| 23 | 1 | 0 | -8.154286 | -0.871505 | -1.156912 |
| 24 | 1 | 0 | -8.192932 | -2.558409 | -0.393057 |
| 25 | 6 | 0 | -1.773492 | 0.628170 | 2.011769 |
| 26 | 1 | 0 | -2.034035 | 0.307982 | 3.027252 |
| 27 | 1 | 0 | -1.255274 | 1.584810 | 2.143132 |
| 28 | 1 | 0 | -5.565476 | 0.645841 | 0.340100 |
| 29 | 8 | 0 | -0.535142 | -2.149271 | -0.196517 |
| 30 | 1 | 0 | -3.782584 | 1.352258 | 1.925519 |
| 31 | 6 | 0 | -4.338180 | -1.246547 | 1.867734 |
| 32 | 1 | 0 | -5.051300 | -0.644727 | 2.437351 |
| 33 | 1 | 0 | -4.857482 | -2.134992 | 1.509280 |
| 34 | 1 | 0 | -3.544523 | -1.576217 | 2.544661 |
| 35 | 8 | 0 | 0.407148 | 2.502448 | -0.170106 |
| 36 | 1 | 0 | -0.679937 | 1.168382 | -1.219199 |
| 37 | 1 | 0 | 0.402203 | -1.977217 | -0.003121 |
| 38 | 1 | 0 | 1.631953 | 1.563072 | -0.535700 |
| 39 | 8 | 0 | 2.302874 | 0.876580 | -0.847472 |
| 40 | 16 | 0 | 2.760629 | -0.083645 | 0.324322 |
| 41 | 8 | 0 | 2.534779 | 0.588732 | 1.588845 |
| 42 | 8 | 0 | 2.148699 | -1.388192 | 0.111040 |
| 43 | 6 | 0 | 4.491938 | -0.234012 | 0.013998 |
| 44 | 6 | 0 | 5.347603 | 0.731357 | 0.533743 |
| 45 | 6 | 0 | 4.959073 | -1.296669 | -0.746569 |
| 46 | 6 | 0 | 6.705219 | 0.621835 | 0.275040 |
| 47 | 1 | 0 | 4.953845 | 1.543416 | 1.133209 |
| 48 | 6 | 0 | 6.323658 | -1.387078 | -0.993311 |
| 49 | 1 | 0 | 4.267415 | -2.037500 | -1.127967 |
| 50 | 6 | 0 | 7.211254 | -0.435860 | -0.488054 |
| 51 | 1 | 0 | 7.386080 | 1.365529 | 0.674124 |
| 52 | 1 | 0 | 6.703800 | -2.211626 | -1.585638 |
| 53 | 6 | 0 | 8.691387 | -0.554855 | -0.731802 |
| 54 | 1 | 0 | 9.135488 | 0.422576 | -0.925950 |
| 55 | 1 | 0 | 9.186636 | -0.974772 | 0.147783 |
| 56 | 1 | 0 | 8.900886 | -1.207563 | -1.579260 |

---------------------------------------------------------------------

## TS1

Standard orientation:

| Center Number | Atomic Number | Atomic Type | Coordinates (Angstroms) X | Y | Z |
|---|---|---|---|---|---|
| 1 | 6 | 0 | -3.269614 | 1.166397 | 1.013171 |
| 2 | 6 | 0 | -1.962843 | 2.363984 | -0.814239 |
| 3 | 6 | 0 | -0.905528 | 0.459860 | 1.184030 |
| 4 | 6 | 0 | -0.665400 | 1.589729 | -0.790132 |
| 5 | 1 | 0 | -1.835783 | 3.279667 | -0.233525 |
| 6 | 1 | 0 | -2.129563 | 2.644274 | -1.860942 |
| 7 | 1 | 0 | 0.126190 | 0.639500 | 1.470322 |
| 8 | 7 | 0 | -3.093984 | 1.599088 | -0.376835 |
| 9 | 6 | 0 | -3.943307 | 0.944607 | -1.223698 |
| 10 | 6 | 0 | -4.775119 | -0.012572 | -0.361533 |
| 11 | 6 | 0 | -3.790426 | -0.272737 | 0.811097 |
| 12 | 6 | 0 | -1.232224 | -0.744440 | 0.620872 |
| 13 | 6 | 0 | -2.636449 | -1.175645 | 0.310914 |
| 14 | 1 | 0 | -2.751727 | -2.192851 | 0.697894 |
| 15 | 1 | 0 | -2.656771 | -1.287694 | -0.780261 |
| 16 | 8 | 0 | -3.985708 | 1.078296 | -2.428318 |
| 17 | 6 | 0 | -5.335517 | -1.170503 | -1.191807 |
| 18 | 1 | 0 | -5.892988 | -0.725870 | -2.019950 |
| 19 | 1 | 0 | -4.518048 | -1.738602 | -1.644562 |
| 20 | 6 | 0 | -6.232988 | -2.094543 | -0.415989 |
| 21 | 1 | 0 | -5.750998 | -2.837955 | 0.214282 |
| 22 | 6 | 0 | -7.560102 | -2.055059 | -0.464793 |
| 23 | 1 | 0 | -8.077875 | -1.338202 | -1.095305 |
| 24 | 1 | 0 | -8.170694 | -2.736614 | 0.115524 |
| 25 | 6 | 0 | -1.985666 | 1.292148 | 1.814448 |
| 26 | 1 | 0 | -2.179318 | 0.989867 | 2.848068 |
| 27 | 1 | 0 | -1.675353 | 2.339550 | 1.864486 |
| 28 | 1 | 0 | -5.621922 | 0.561019 | 0.040346 |
| 29 | 8 | 0 | -0.354635 | -1.616385 | 0.170877 |
| 30 | 1 | 0 | -4.056208 | 1.765266 | 1.489042 |
| 31 | 6 | 0 | -4.422086 | -0.810674 | 2.088908 |
| 32 | 1 | 0 | -5.246267 | -0.170557 | 2.413758 |
| 33 | 1 | 0 | -4.812319 | -1.818601 | 1.950575 |
| 34 | 1 | 0 | -3.680120 | -0.852701 | 2.891663 |
| 35 | 8 | 0 | 0.385634 | 2.272917 | -0.607189 |
| 36 | 1 | 0 | -0.592912 | 0.650356 | -1.344256 |
| 37 | 1 | 0 | 0.590818 | -1.382318 | 0.397065 |
| 38 | 1 | 0 | 1.283585 | 1.658334 | -0.727397 |
| 39 | 8 | 0 | 2.269671 | 0.783743 | -0.835769 |
| 40 | 16 | 0 | 2.806475 | 0.186900 | 0.450726 |
| 41 | 8 | 0 | 2.753809 | 1.146967 | 1.547228 |
| 42 | 8 | 0 | 2.127030 | -1.102121 | 0.716624 |
| 43 | 6 | 0 | 4.501780 | -0.200555 | 0.106352 |
| 44 | 6 | 0 | 5.483772 | 0.716012 | 0.455992 |
| 45 | 6 | 0 | 4.814785 | -1.390120 | -0.540481 |
| 46 | 6 | 0 | 6.808125 | 0.429532 | 0.146838 |
| 47 | 1 | 0 | 5.211484 | 1.631016 | 0.967859 |
| 48 | 6 | 0 | 6.142814 | -1.658430 | -0.841358 |
| 49 | 1 | 0 | 4.029564 | -2.092434 | -0.792551 |
| 50 | 6 | 0 | 7.155549 | -0.756648 | -0.502059 |
| 51 | 1 | 0 | 7.583704 | 1.137872 | 0.416586 |
| 52 | 1 | 0 | 6.399594 | -2.584044 | -1.345024 |
| 53 | 6 | 0 | 8.595362 | -1.076473 | -0.804401 |
| 54 | 1 | 0 | 8.686639 | -1.629185 | -1.740472 |
| 55 | 1 | 0 | 9.194129 | -0.168065 | -0.877184 |
| 56 | 1 | 0 | 9.021526 | -1.695590 | -0.010229 |

## *epi*-TS1

Standard orientation:

| Center Number | Atomic Number | Atomic Type | Coordinates (Angstroms) X | Y | Z |
|---|---|---|---|---|---|
| 1 | 6 | 0 | 2.347660 | -1.386366 | 1.199430 |
| 2 | 6 | 0 | 0.506714 | -1.888076 | -0.496132 |
| 3 | 6 | 0 | 0.466174 | 0.168373 | 1.635078 |
| 4 | 6 | 0 | -0.287604 | -0.592971 | -0.495201 |
| 5 | 1 | 0 | 0.028722 | -2.608049 | 0.167537 |
| 6 | 1 | 0 | 0.457220 | -2.272995 | -1.519899 |
| 7 | 1 | 0 | -0.530408 | 0.386965 | 2.002840 |
| 8 | 7 | 0 | 1.893345 | -1.714749 | -0.156817 |
| 9 | 6 | 0 | 2.857859 | -1.429820 | -1.087942 |
| 10 | 6 | 0 | 4.052355 | -0.836865 | -0.326767 |
| 11 | 6 | 0 | 3.341144 | -0.239690 | 0.917401 |
| 12 | 6 | 0 | 1.157256 | 1.161109 | 1.006368 |
| 13 | 6 | 0 | 2.556108 | 1.021541 | 0.491336 |
| 14 | 1 | 0 | 3.107862 | 1.929852 | 0.751408 |
| 15 | 1 | 0 | 2.425958 | 1.064785 | -0.599290 |
| 16 | 8 | 0 | 2.755778 | -1.587149 | -2.283872 |
| 17 | 6 | 0 | 4.898720 | 0.051496 | -1.243737 |
| 18 | 1 | 0 | 5.211416 | -0.568347 | -2.087575 |
| 19 | 1 | 0 | 4.278927 | 0.848908 | -1.663263 |
| 20 | 6 | 0 | 6.106883 | 0.642274 | -0.571887 |
| 21 | 1 | 0 | 5.944165 | 1.517055 | 0.053486 |
| 22 | 6 | 0 | 7.340609 | 0.168043 | -0.706885 |
| 23 | 1 | 0 | 7.545485 | -0.692368 | -1.337141 |
| 24 | 1 | 0 | 8.183500 | 0.623887 | -0.200997 |
| 25 | 6 | 0 | 1.203857 | -1.040402 | 2.136087 |
| 26 | 1 | 0 | 1.614500 | -0.872379 | 3.137054 |
| 27 | 1 | 0 | 0.528836 | -1.894051 | 2.235914 |
| 28 | 1 | 0 | 4.677441 | -1.672758 | 0.016834 |
| 29 | 8 | 0 | 0.616853 | 2.302038 | 0.622336 |
| 30 | 1 | 0 | 2.902561 | -2.238929 | 1.610571 |
| 31 | 6 | 0 | 4.234977 | 0.019808 | 2.123528 |
| 32 | 1 | 0 | 4.776920 | -0.886308 | 2.406603 |
| 33 | 1 | 0 | 4.967932 | 0.800159 | 1.921090 |
| 34 | 1 | 0 | 3.635802 | 0.345016 | 2.979196 |
| 35 | 8 | 0 | -0.044628 | 0.273079 | -1.376318 |
| 36 | 1 | 0 | -1.276310 | -0.590312 | -0.024918 |
| 37 | 1 | 0 | -0.348608 | 2.338316 | 0.838281 |
| 38 | 1 | 0 | -0.884934 | 1.018499 | -1.439348 |
| 39 | 8 | 0 | -1.983946 | 2.078188 | 0.925119 |
| 40 | 16 | 0 | -2.816997 | 2.002600 | -0.298951 |
| 41 | 8 | 0 | -1.932399 | 1.758198 | -1.507077 |
| 42 | 6 | 0 | -3.757602 | 0.497128 | -0.128290 |
| 43 | 6 | 0 | -3.986759 | -0.030932 | 1.135388 |
| 44 | 6 | 0 | -4.265421 | -0.116249 | -1.269652 |
| 45 | 6 | 0 | -4.737282 | -1.196768 | 1.253009 |
| 46 | 1 | 0 | -3.575725 | 0.462167 | 2.008148 |
| 47 | 6 | 0 | -5.011661 | -1.278146 | -1.133027 |
| 48 | 1 | 0 | -4.067943 | 0.308003 | -2.247235 |
| 49 | 6 | 0 | -5.260563 | -1.833086 | 0.126711 |
| 50 | 1 | 0 | -4.918957 | -1.616879 | 2.236271 |
| 51 | 1 | 0 | -5.407270 | -1.765479 | -2.017656 |
| 52 | 8 | 0 | -3.722069 | 3.110329 | -0.524500 |
| 53 | 6 | 0 | -6.098207 | -3.077008 | 0.258374 |
| 54 | 1 | 0 | -7.160243 | -2.826713 | 0.190251 |
| 55 | 1 | 0 | -5.875360 | -3.786999 | -0.539606 |
| 56 | 1 | 0 | -5.930954 | -3.567227 | 1.217737 |

## **7**-*p*TsOH

Standard orientation:

| Center Number | Atomic Number | Atomic Type | Coordinates (Angstroms) X | Y | Z |
|---|---|---|---|---|---|
| 1 | 6 | 0 | 3.307089 | -1.255032 | 0.676736 |
| 2 | 6 | 0 | 2.381870 | -2.289319 | -1.424540 |
| 3 | 6 | 0 | 0.875297 | -1.714885 | 0.589166 |
| 4 | 6 | 0 | 0.945521 | -2.012736 | -0.954503 |
| 5 | 1 | 0 | 2.640111 | -3.323434 | -1.185569 |
| 6 | 1 | 0 | 2.459799 | -2.158313 | -2.502644 |
| 7 | 1 | 0 | 0.041009 | -2.291443 | 0.989648 |
| 8 | 7 | 0 | 3.303645 | -1.370484 | -0.785400 |
| 9 | 6 | 0 | 3.733405 | -0.215471 | -1.373537 |
| 10 | 6 | 0 | 4.123121 | 0.753724 | -0.246392 |
| 11 | 6 | 0 | 3.173872 | 0.271356 | 0.880572 |
| 12 | 6 | 0 | 0.636794 | -0.259454 | 0.908348 |
| 13 | 6 | 0 | 1.723943 | 0.718248 | 0.541861 |
| 14 | 1 | 0 | 1.491460 | 1.685910 | 0.989514 |
| 15 | 1 | 0 | 1.617868 | 0.836749 | -0.546002 |
| 16 | 8 | 0 | 3.740487 | 0.021620 | -2.563592 |
| 17 | 6 | 0 | 4.071882 | 2.205532 | -0.729017 |
| 18 | 1 | 0 | 4.723875 | 2.273286 | -1.603092 |
| 19 | 1 | 0 | 3.063427 | 2.441617 | -1.081994 |
| 20 | 6 | 0 | 4.501895 | 3.208651 | 0.305223 |
| 21 | 1 | 0 | 3.770057 | 3.485832 | 1.060432 |
| 22 | 6 | 0 | 5.705652 | 3.769924 | 0.341327 |
| 23 | 1 | 0 | 6.458737 | 3.531866 | -0.404113 |
| 24 | 1 | 0 | 5.976846 | 4.484757 | 1.109203 |
| 25 | 6 | 0 | 2.191676 | -2.095249 | 1.278522 |
| 26 | 1 | 0 | 2.139627 | -1.925741 | 2.356687 |
| 27 | 1 | 0 | 2.389801 | -3.159394 | 1.134490 |
| 28 | 1 | 0 | 5.151962 | 0.524005 | 0.062145 |
| 29 | 8 | 0 | -0.390832 | 0.136247 | 1.437359 |
| 30 | 1 | 0 | 4.275848 | -1.579307 | 1.076226 |
| 31 | 6 | 0 | 3.564226 | 0.683130 | 2.294624 |
| 32 | 1 | 0 | 4.593701 | 0.386822 | 2.509975 |
| 33 | 1 | 0 | 3.487210 | 1.760765 | 2.435894 |
| 34 | 1 | 0 | 2.909682 | 0.208123 | 3.031434 |
| 35 | 8 | 0 | 0.204497 | -3.162980 | -1.270300 |
| 36 | 1 | 0 | 0.550877 | -1.148335 | -1.503079 |
| 37 | 1 | 0 | -1.677918 | -0.788492 | 1.666625 |
| 38 | 1 | 0 | -0.730933 | -2.927844 | -1.193710 |
| 39 | 8 | 0 | -2.006210 | -1.586277 | -0.701197 |
| 40 | 16 | 0 | -3.122218 | -1.473855 | 0.233509 |
| 41 | 8 | 0 | -4.117833 | -2.513873 | 0.292617 |
| 42 | 8 | 0 | -2.512171 | -1.350304 | 1.690101 |
| 43 | 6 | 0 | -3.909491 | 0.084744 | -0.049544 |
| 44 | 6 | 0 | -5.265814 | 0.212630 | 0.219642 |
| 45 | 6 | 0 | -3.150407 | 1.153487 | -0.512898 |
| 46 | 6 | 0 | -5.869685 | 1.447354 | 0.020121 |
| 47 | 1 | 0 | -5.832477 | -0.640906 | 0.570885 |
| 48 | 6 | 0 | -3.773008 | 2.378396 | -0.704136 |
| 49 | 1 | 0 | -2.096003 | 1.022259 | -0.724215 |
| 50 | 6 | 0 | -5.136805 | 2.541845 | -0.443950 |
| 51 | 1 | 0 | -6.928390 | 1.561222 | 0.224347 |
| 52 | 1 | 0 | -3.193854 | 3.220658 | -1.066038 |
| 53 | 6 | 0 | -5.806842 | 3.866506 | -0.690360 |
| 54 | 1 | 0 | -5.125806 | 4.694657 | -0.490668 |
| 55 | 1 | 0 | -6.123650 | 3.940685 | -1.734248 |
| 56 | 1 | 0 | -6.691538 | 3.983311 | -0.063967 |

## *epi*-**7**-*p*TsOH

Standard orientation:

| Center Number | Atomic Number | Atomic Type | Coordinates (Angstroms) X | Y | Z |
|---|---|---|---|---|---|
| 1 | 6 | 0 | -3.255207 | -1.463249 | -0.459227 |
| 2 | 6 | 0 | -2.202098 | -2.173866 | 1.715755 |
| 3 | 6 | 0 | -0.816021 | -1.909083 | -0.443415 |
| 4 | 6 | 0 | -0.796586 | -2.000354 | 1.123563 |
| 5 | 1 | 0 | -2.497158 | -3.225254 | 1.682951 |
| 6 | 1 | 0 | -2.192809 | -1.849871 | 2.756224 |
| 7 | 1 | 0 | 0.005764 | -2.506085 | -0.840196 |
| 8 | 7 | 0 | -3.172058 | -1.365898 | 1.001148 |
| 9 | 6 | 0 | -3.616895 | -0.151415 | 1.441991 |
| 10 | 6 | 0 | -4.061001 | 0.646915 | 0.204963 |
| 11 | 6 | 0 | -3.156821 | 0.017682 | -0.885874 |
| 12 | 6 | 0 | -0.609609 | -0.488572 | -0.926710 |
| 13 | 6 | 0 | -1.701951 | 0.517106 | -0.682041 |
| 14 | 1 | 0 | -1.497776 | 1.405576 | -1.282289 |
| 15 | 1 | 0 | -1.540274 | 0.787738 | 0.370351 |
| 16 | 8 | 0 | -3.614333 | 0.239887 | 2.589302 |
| 17 | 6 | 0 | -4.002820 | 2.151996 | 0.479724 |
| 18 | 1 | 0 | -4.620005 | 2.338548 | 1.361772 |
| 19 | 1 | 0 | -2.982460 | 2.435259 | 0.754971 |
| 20 | 6 | 0 | -4.481279 | 3.001336 | -0.664627 |
| 21 | 1 | 0 | -3.785549 | 3.163687 | -1.484728 |
| 22 | 6 | 0 | -5.685109 | 3.560664 | -0.724053 |
| 23 | 1 | 0 | -6.402293 | 3.435779 | 0.081901 |
| 24 | 1 | 0 | -5.991745 | 4.161836 | -1.571884 |
| 25 | 6 | 0 | -2.165734 | -2.374917 | -1.002364 |
| 26 | 1 | 0 | -2.172668 | -2.346308 | -2.094685 |
| 27 | 1 | 0 | -2.353732 | -3.411744 | -0.714587 |
| 28 | 1 | 0 | -5.100503 | 0.373681 | -0.022490 |
| 29 | 8 | 0 | 0.418498 | -0.148410 | -1.492088 |
| 30 | 1 | 0 | -4.239731 | -1.848089 | -0.753297 |
| 31 | 6 | 0 | -3.622098 | 0.219416 | -2.322708 |
| 32 | 1 | 0 | -4.654579 | -0.117339 | -2.444728 |
| 33 | 1 | 0 | -3.572769 | 1.267536 | -2.616681 |
| 34 | 1 | 0 | -2.992373 | -0.344869 | -3.017096 |
| 35 | 1 | 0 | 1.728932 | -1.047565 | -1.676648 |
| 36 | 8 | 0 | 2.169649 | -1.825468 | 0.708890 |
| 37 | 16 | 0 | 3.242713 | -1.579969 | -0.249656 |
| 38 | 8 | 0 | 4.358690 | -2.489233 | -0.312257 |
| 39 | 8 | 0 | 2.596142 | -1.557795 | -1.694085 |
| 40 | 6 | 0 | 3.837156 | 0.069503 | -0.001631 |
| 41 | 6 | 0 | 5.183852 | 0.335286 | -0.208071 |
| 42 | 6 | 0 | 2.943980 | 1.067173 | 0.376601 |
| 43 | 6 | 0 | 5.642079 | 1.634583 | -0.026821 |
| 44 | 1 | 0 | 5.855483 | -0.463601 | -0.497078 |
| 45 | 6 | 0 | 3.421772 | 2.357276 | 0.550292 |
| 46 | 1 | 0 | 1.894828 | 0.842434 | 0.531116 |
| 47 | 6 | 0 | 4.773514 | 2.658259 | 0.354998 |
| 48 | 1 | 0 | 6.692250 | 1.854752 | -0.181903 |
| 49 | 1 | 0 | 2.736198 | 3.143741 | 0.845722 |
| 50 | 6 | 0 | 5.281349 | 4.056488 | 0.581538 |
| 51 | 1 | 0 | 4.557796 | 4.796741 | 0.237246 |
| 52 | 1 | 0 | 5.449857 | 4.228907 | 1.647962 |
| 53 | 1 | 0 | 6.224956 | 4.222522 | 0.061587 |
| 54 | 8 | 0 | -0.208146 | -0.846419 | 1.697617 |
| 55 | 1 | 0 | 0.748187 | -0.950760 | 1.593803 |
| 56 | 1 | 0 | -0.203661 | -2.880314 | 1.388799 |

X-ray crystallographic data of tosylate **8** (CCDC-2152208)

Compound **8** was crystallized from a mixture of dichloromethane and diethyl ether.

A plate-like specimen of $C_{43}H_{51}Cl_2N_2O_{10}S_2$, approximate dimensions 0.040 mm x 0.080 mm x 0.200 mm, was used for the X-ray crystallographic analysis. The X-ray intensity data were measured on a D8 Venture system equipped with a multilayer monochromator and a Mo microfocus ($\lambda$ = 0.71073 Å).

The frames were integrated with the Bruker SAINT software package using a narrow-frame algorithm. The integration of the data using a monoclinic unit cell yielded a total of 35130 reflections to a maximum $\theta$ angle of 26.39° (0.80 Å resolution), of which 8673 were independent (average redundancy 4.051, completeness = 99.8%, $R_{int}$ = 10.61%, $R_{sig}$ = 9.28%) and 5740 (66.18%) were greater than $2\sigma(F^2)$. The final cell constants of a = 10.9498(9) Å, b = 13.6537(12) Å, c= 14.2253(12) Å, $\beta$ = 93.767(4)°, volume = 2122.2(3) Å$^3$, are based upon the refinement of the XYZ-centroids of reflections above 20 $\sigma$(I). Data were corrected for absorption effects using the Multi-Scan method (SADABS). The calculated minimum and maximum transmission coefficients (based on crystal size) are 0.5993 and0.7454.

The structure was solved and refined using the Bruker SHELXTL Software Package, using the space group P 1 21 1, with Z = 2 or the formula unit, $C_{43}H_{51}Cl_2N_2O_{10}S_2$. The final anisotropic full-matrix least-squares refinement on $F^2$ with 535 variables converged at R1 = 9.00%, for the observed data and wR2 = 27.01% for all data. The goodness-of-fit was 1.044. The largest peak in the final difference electron density synthesis was 0.997 $e^-$/Å$^3$ and the largest hole was -1.091 $e^-$/Å$^3$ with an RMS deviation of 0.120 $e^-$/Å$^3$. On the basis of the final model, the calculated density was 1.394 g/cm$^3$ and F(000), 938 $e^-$.

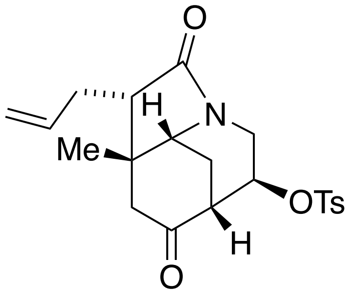


**Datablock D43ZB189_0m_a** - ellipsoid plot

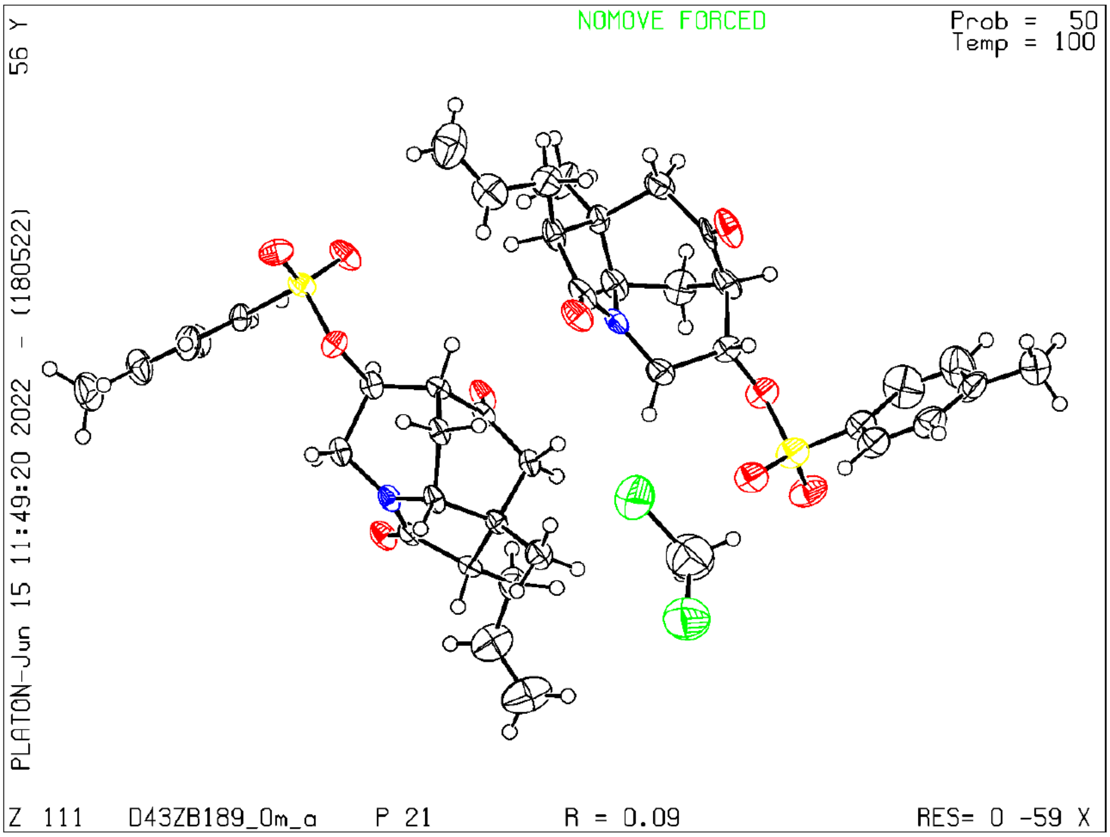

**Table 1.** Crystal data and structure refinement for D43ZB189_0m_A.

| | | |
|---|---|---|
| Identification code | D43ZB189_0m_a | |
| Empirical formula | C43 H51 Cl2 N2 O10 S2 | |
| Formula weight | 890.88 | |
| Temperature | 100(2) K | |
| Wavelength | 0.71073 Å | |
| Crystal system | Monoclinic | |
| Space group | P 21 | |
| Unit cell dimensions | a = 10.9498(9) Å | $\alpha$= 90°. |
| | b = 13.6537(12) Å | $\beta$= 93.767(4)°. |
| | c = 14.2253(12) Å | $\gamma$ = 90°. |
| Volume | 2122.2(3) $Å^3$ | |
| Z | 2 | |
| Density (calculated) | 1.394 $Mg/m^3$ | |
| Absorption coefficient | 0.312 $mm^{-1}$ | |
| F(000) | 938 | |
| Crystal size | 0.200 x 0.080 x 0.040 $mm^3$ | |
| Theta range for data collection | 2.070 to 26.391°. | |
| Index ranges | -13<=h<=13, -17<=k<=17, -17<=l<=17 | |
| Reflections collected | 35130 | |
| Independent reflections | 8673 [R(int) = 0.1061] | |
| Completeness to theta = 25.242° | 99.9 % | |
| Absorption correction | Semi-empirical from equivalents | |
| Max. and min. transmission | 0.7454 and 0.5993 | |
| Refinement method | Full-matrix least-squares on $F^2$ | |
| Data / restraints / parameters | 8673 / 2 / 535 | |
| Goodness-of-fit on $F^2$ | 1.044 | |
| Final R indices [I>2sigma(I)] | R1 = 0.0900, wR2 = 0.2278 | |
| R indices (all data) | R1 = 0.1381, wR2 = 0.2701 | |
| Absolute structure parameter | 0.52(5) | |
| Extinction coefficient | n/a | |
| Largest diff. peak and hole | 0.997 and -1.091 e.$Å^{-3}$ | |

# ¹H- and ¹³C-NMR Spectra of compounds **2-10**

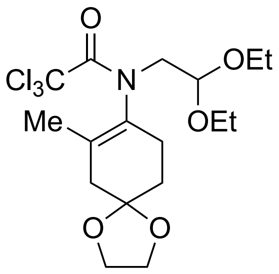


**2**

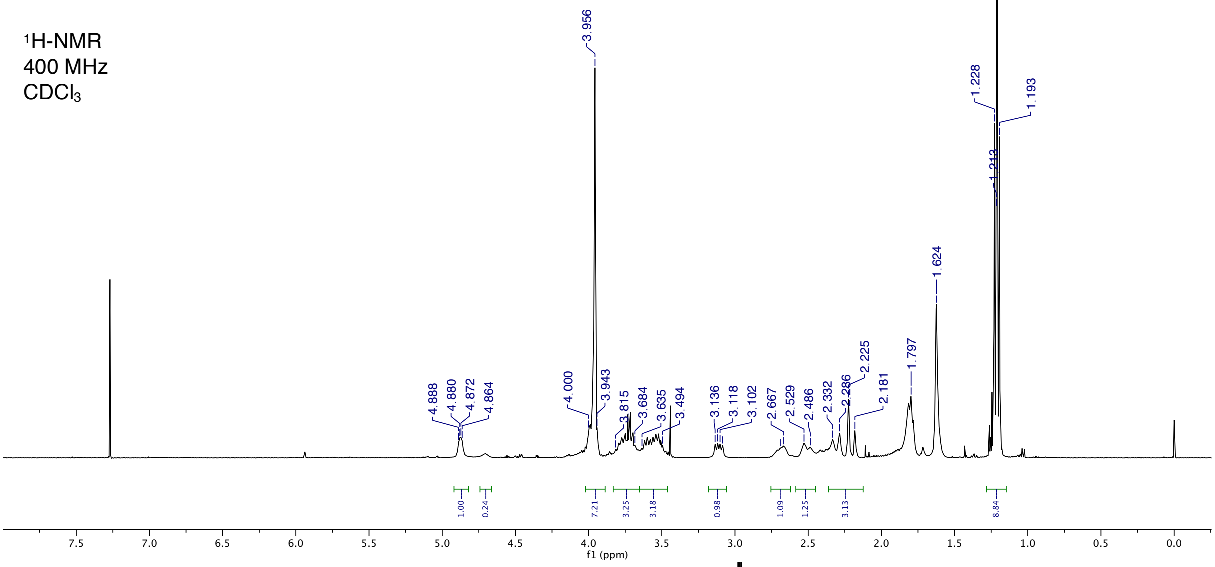


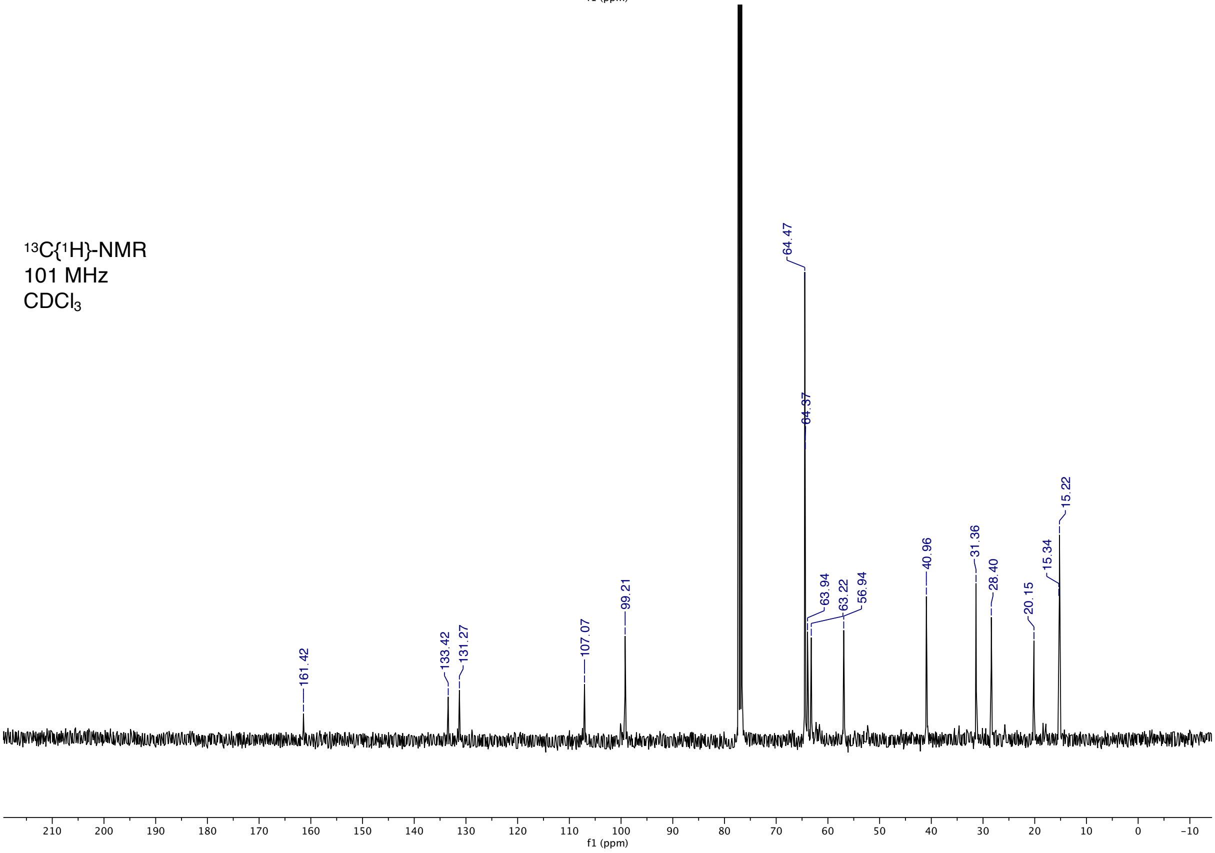

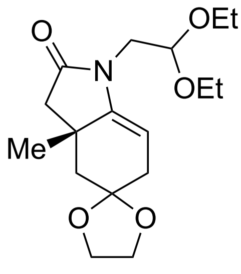


**3**

$^{1}H$-NMR
400 MHz
$CDCl_3$

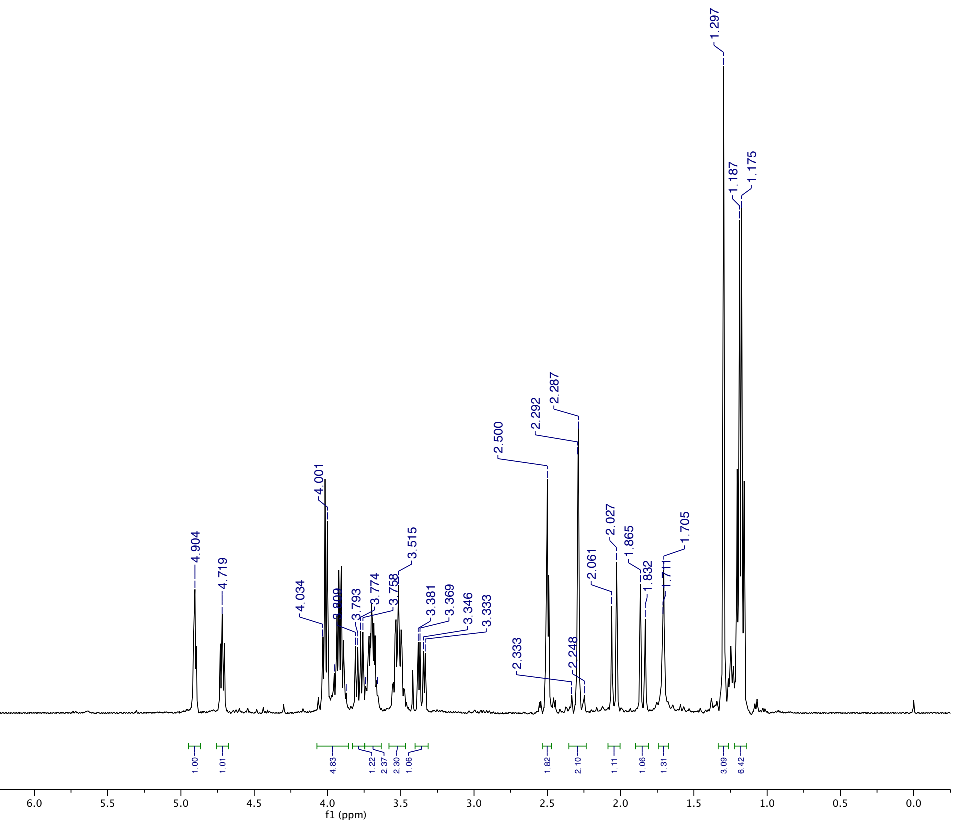


$^{13}C\{^{1}H\}$-NMR
101 MHz
$CDCl_3$

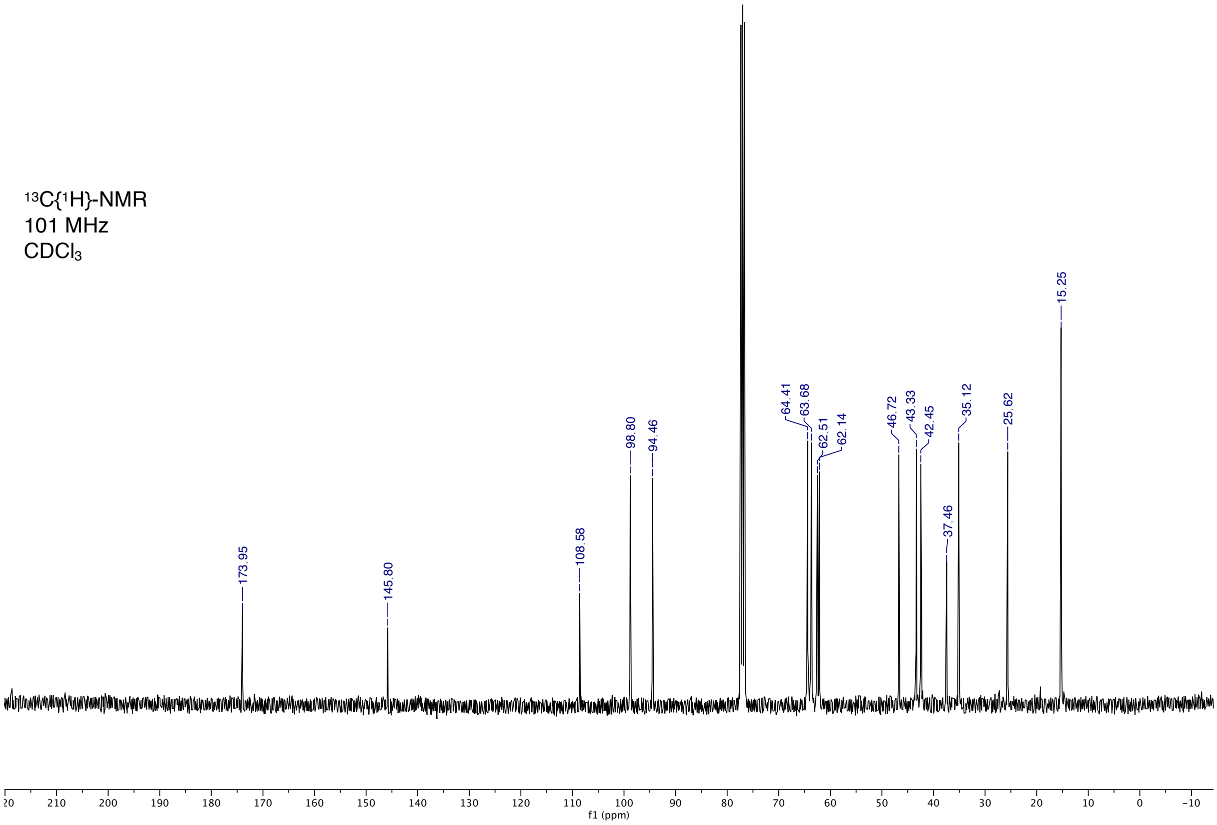

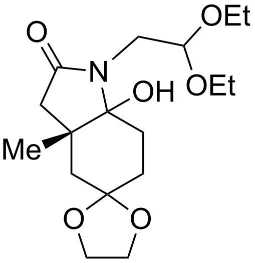


**3’**

$^{1}$H-NMR
400 MHz
$CDCl_3$

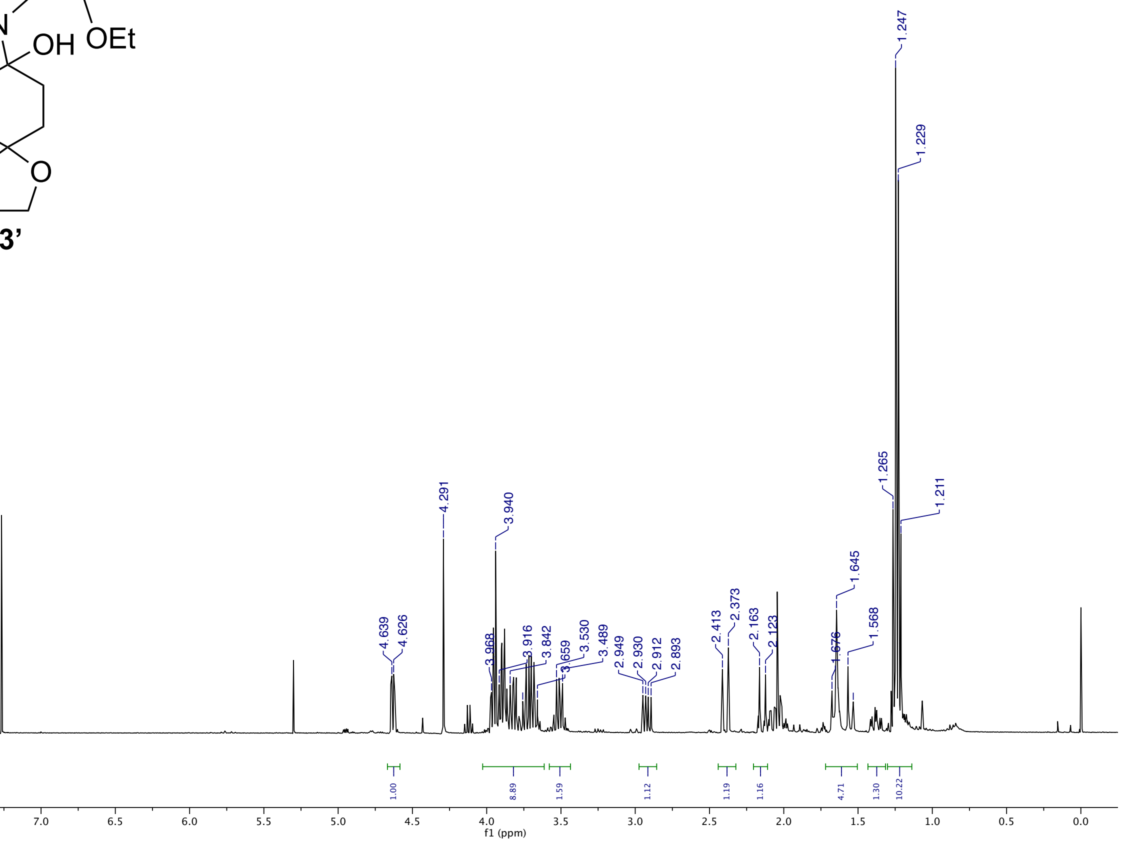


$^{13}$C{$^{1}$H}-NMR
101 MHz
$CDCl_3$

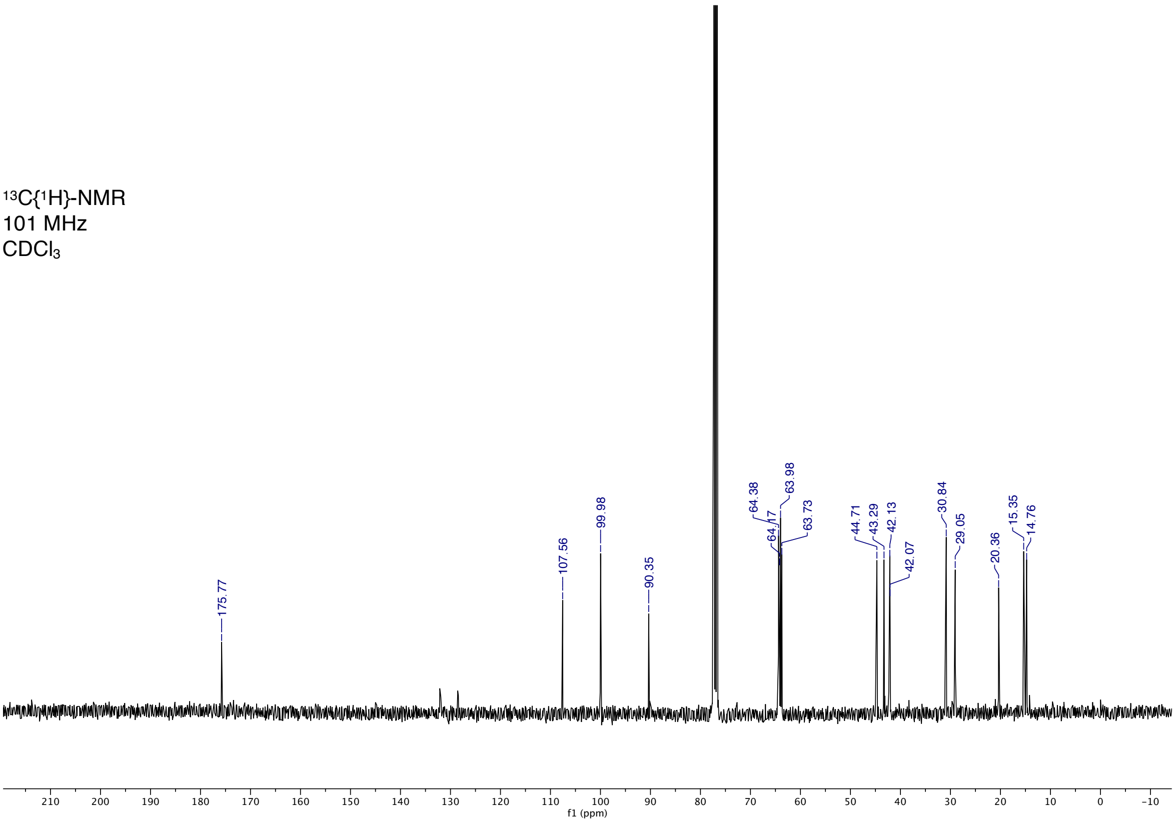

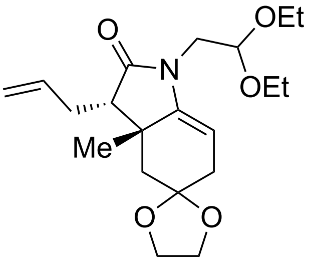

**4**

$^1$H-NMR
400 MHz
$CDCl_3$

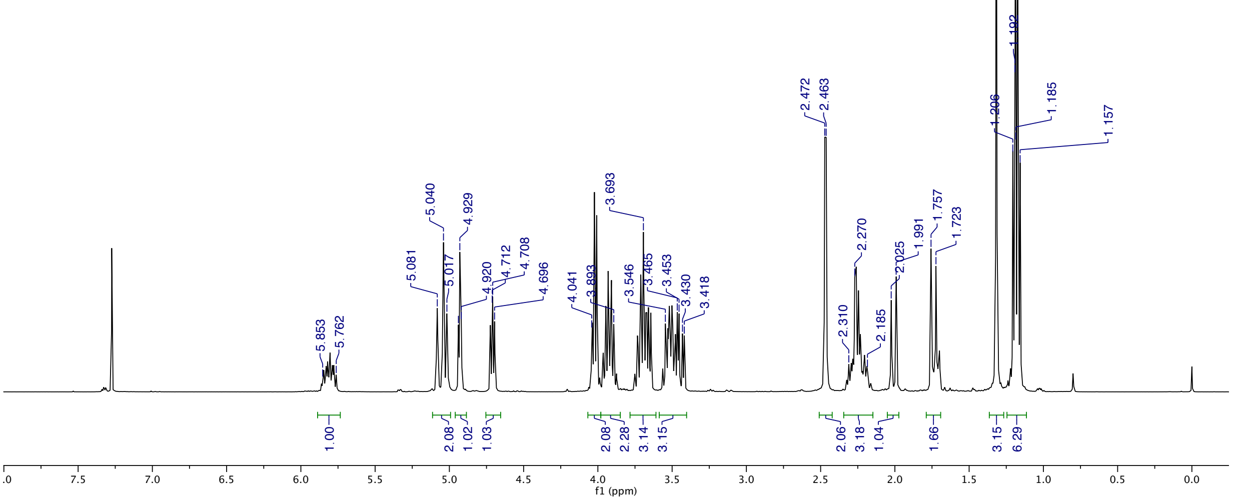


$^{13}$C{$^1$H}-NMR
101 MHz
$CDCl_3$

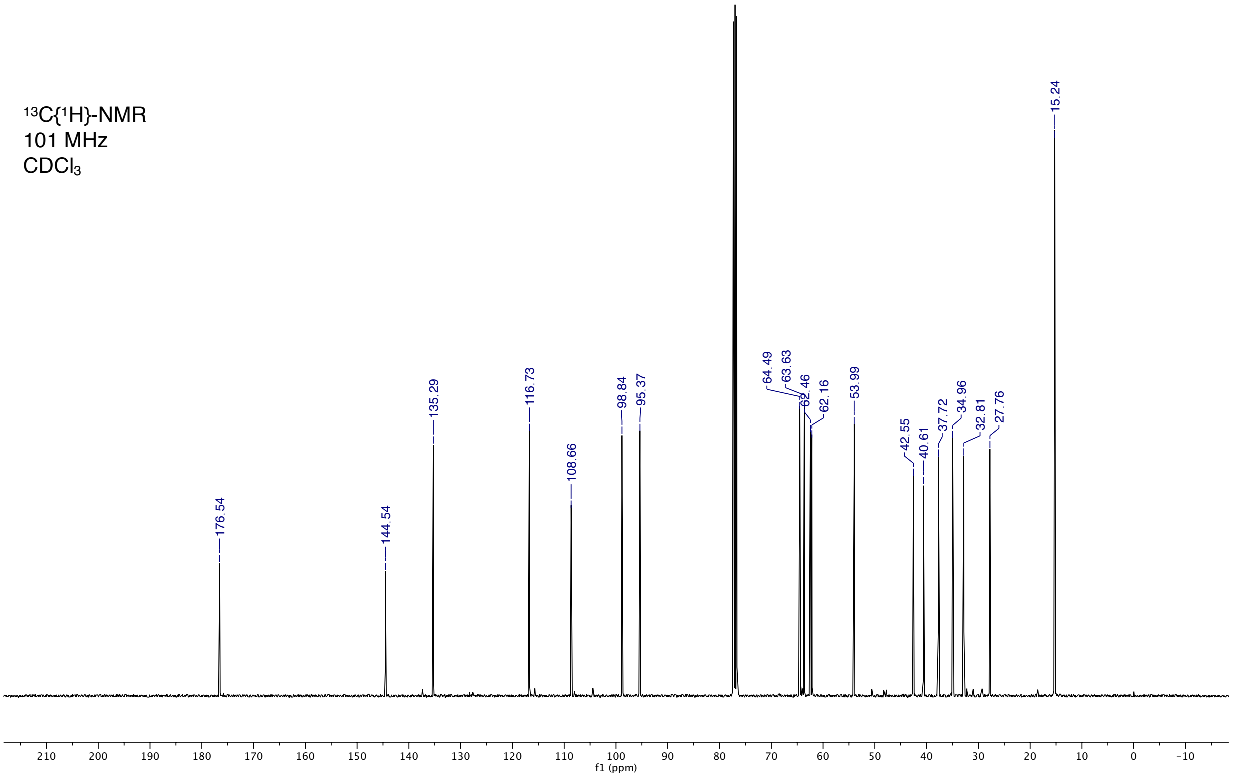

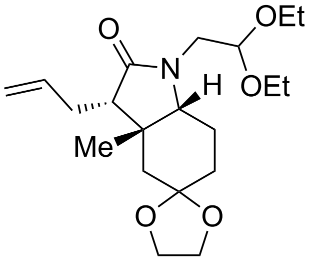


**5**

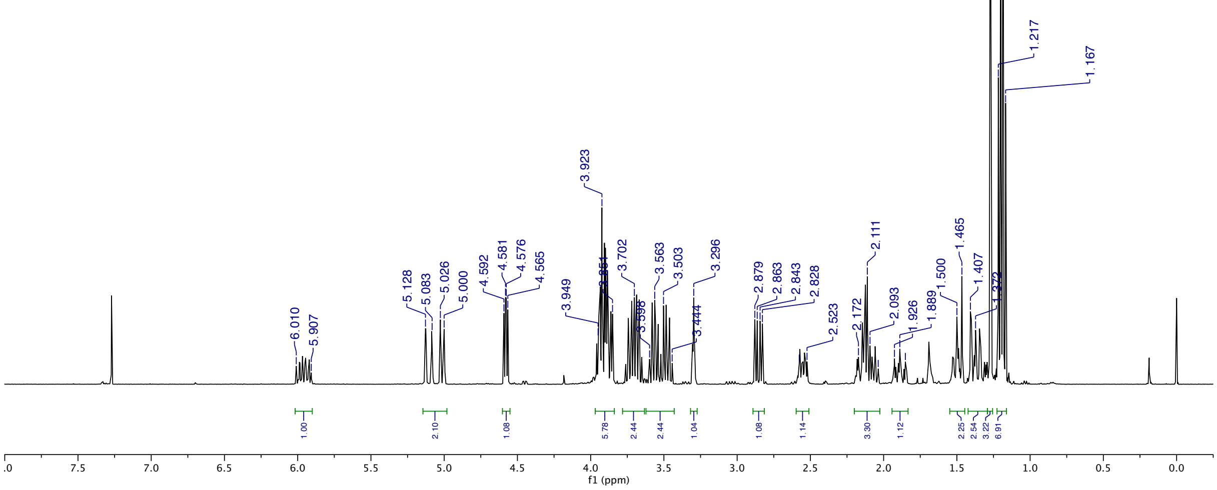


¹H-NMR
400 MHz
$CDCl_3$

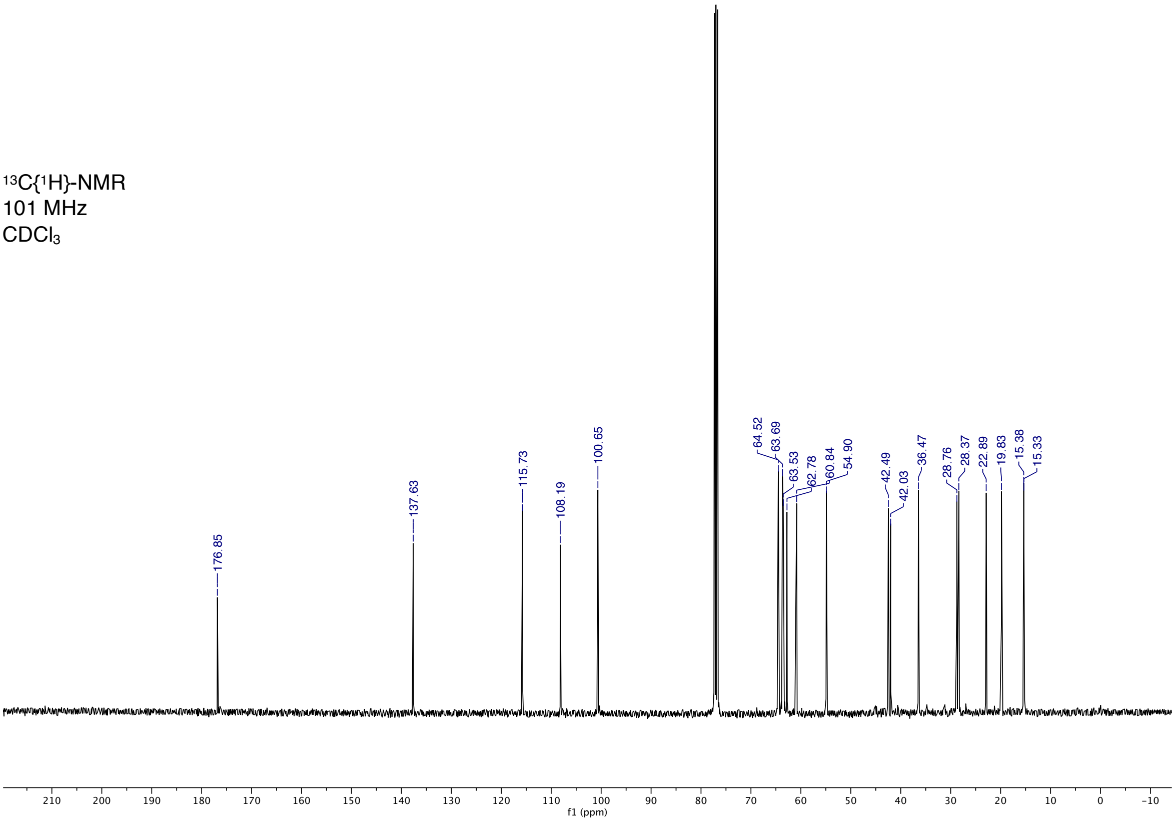


¹³C{¹H}-NMR
101 MHz
$CDCl_3$

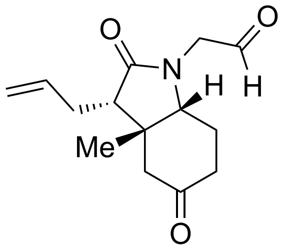

**6**

$^{1}$H-NMR
400 MHz
$CDCl_3$

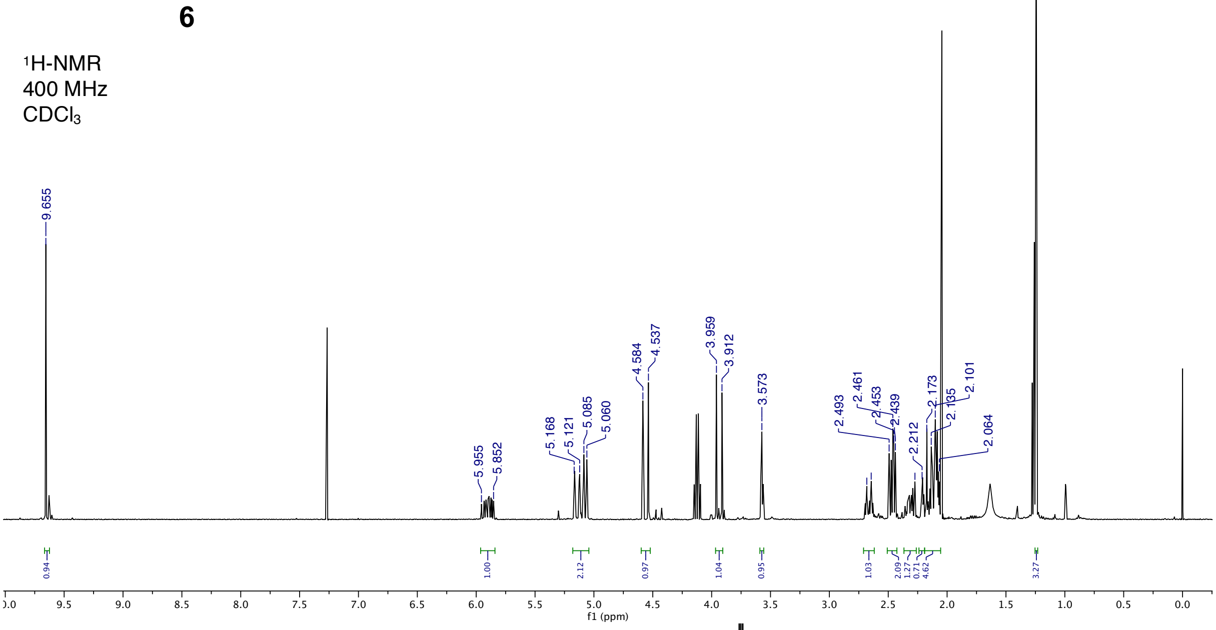


$^{13}$C{$^{1}$H}-NMR
101 MHz
$CDCl_3$

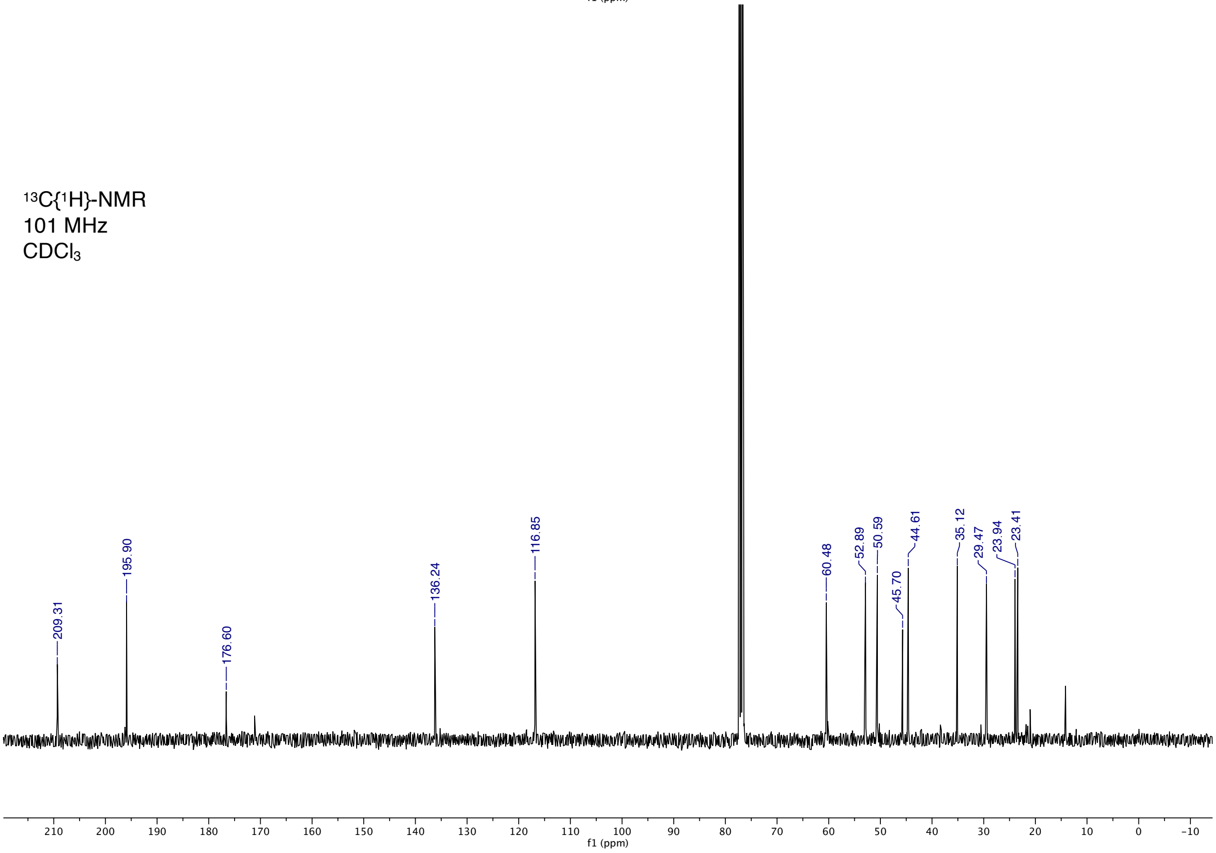

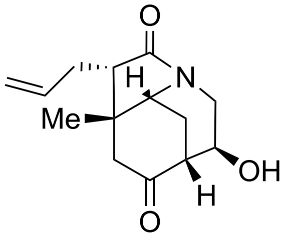


**7**

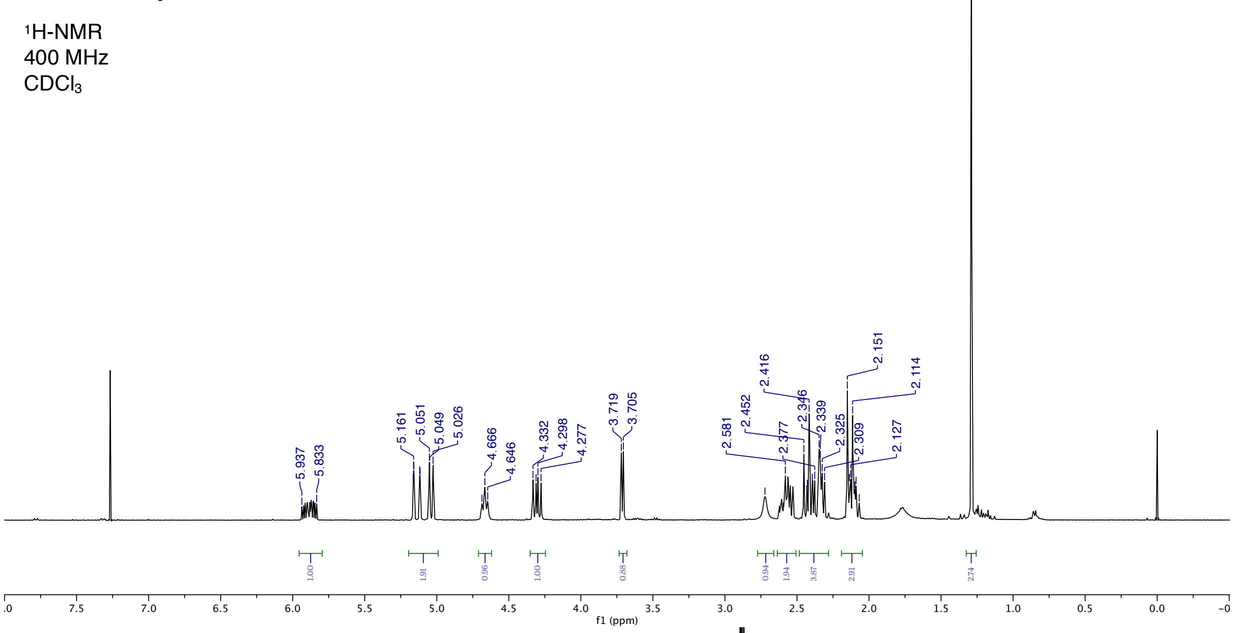


$^1$H-NMR
400 MHz
$CDCl_3$

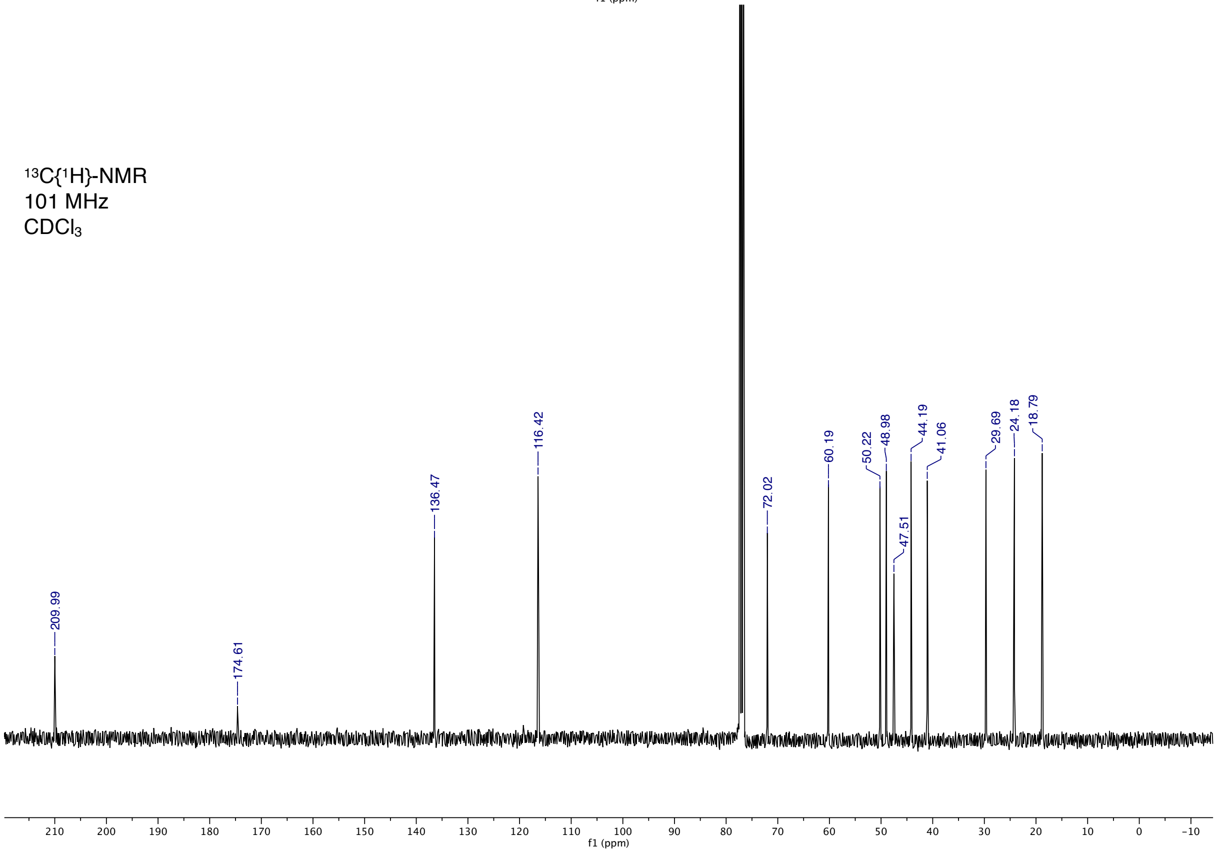


$^{13}C\{^1H\}$-NMR
101 MHz
$CDCl_3$

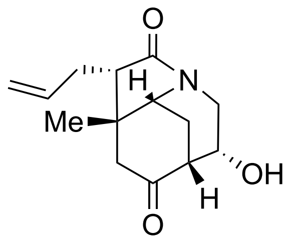


***epi*-7**

$^{1}$H-NMR
400 MHz
$CDCl_3$

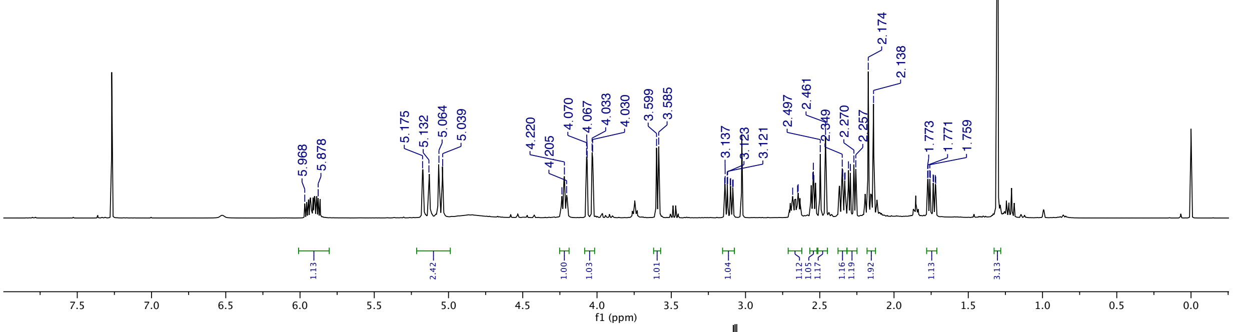


$^{13}$C{$^{1}$H}-NMR
101 MHz
$CDCl_3$

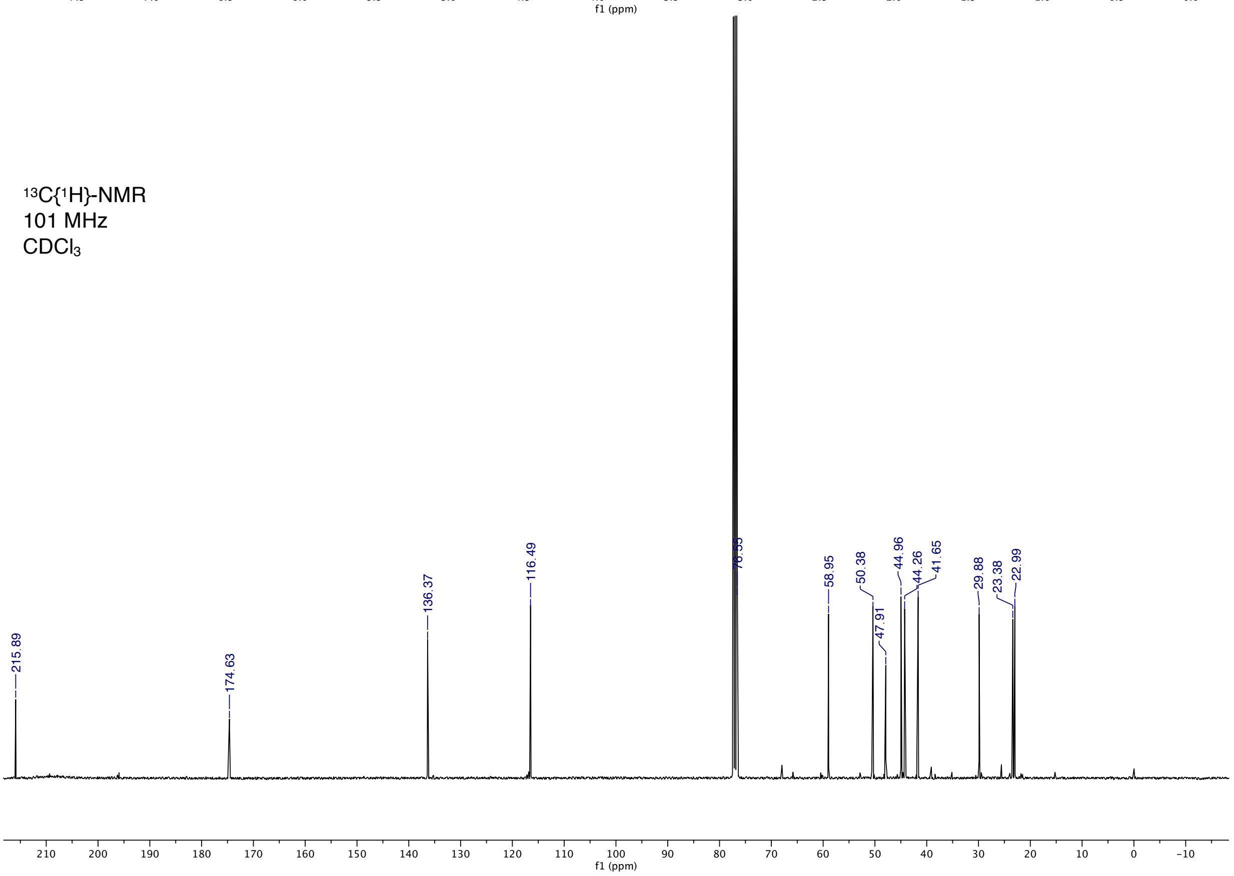

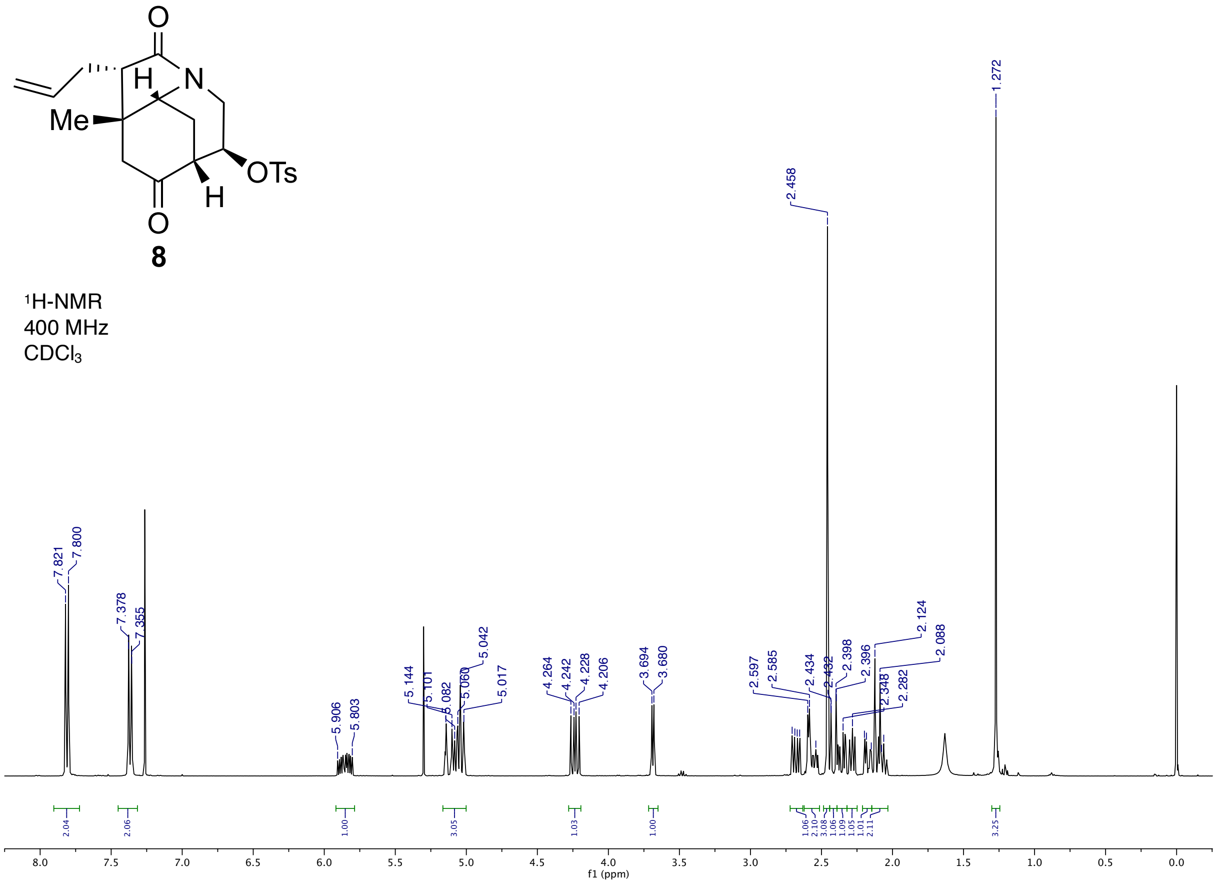


$^{13}$C{$^{1}$H}-NMR
101 MHz
$CDCl_3$

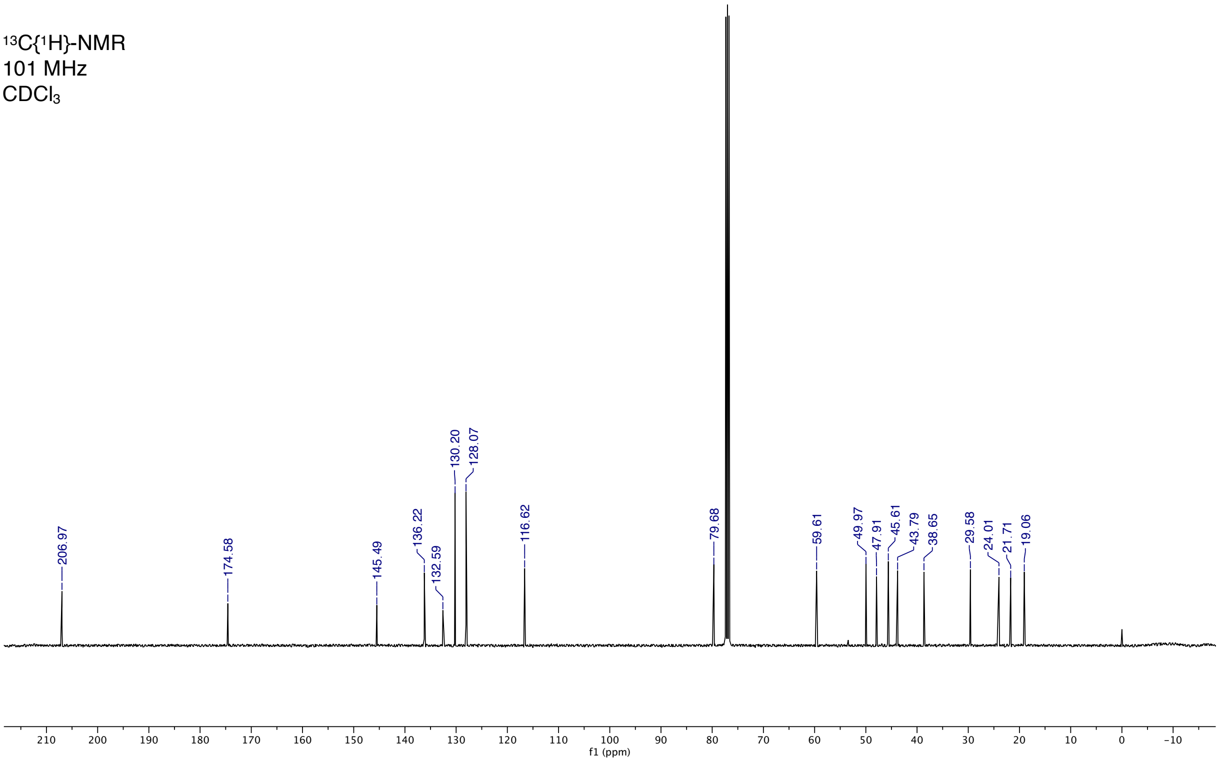

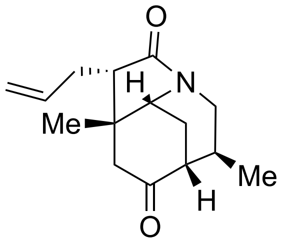

**9**

$^{1}$H-NMR
400 MHz
$CDCl_3$

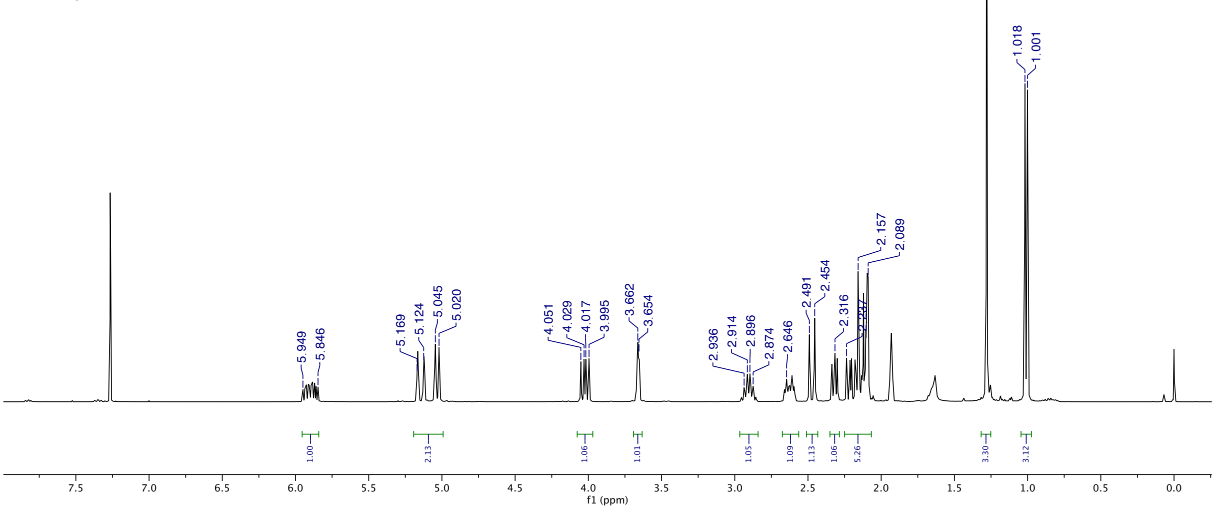


$^{13}$C{$^{1}$H}-NMR
101 MHz
$CDCl_3$

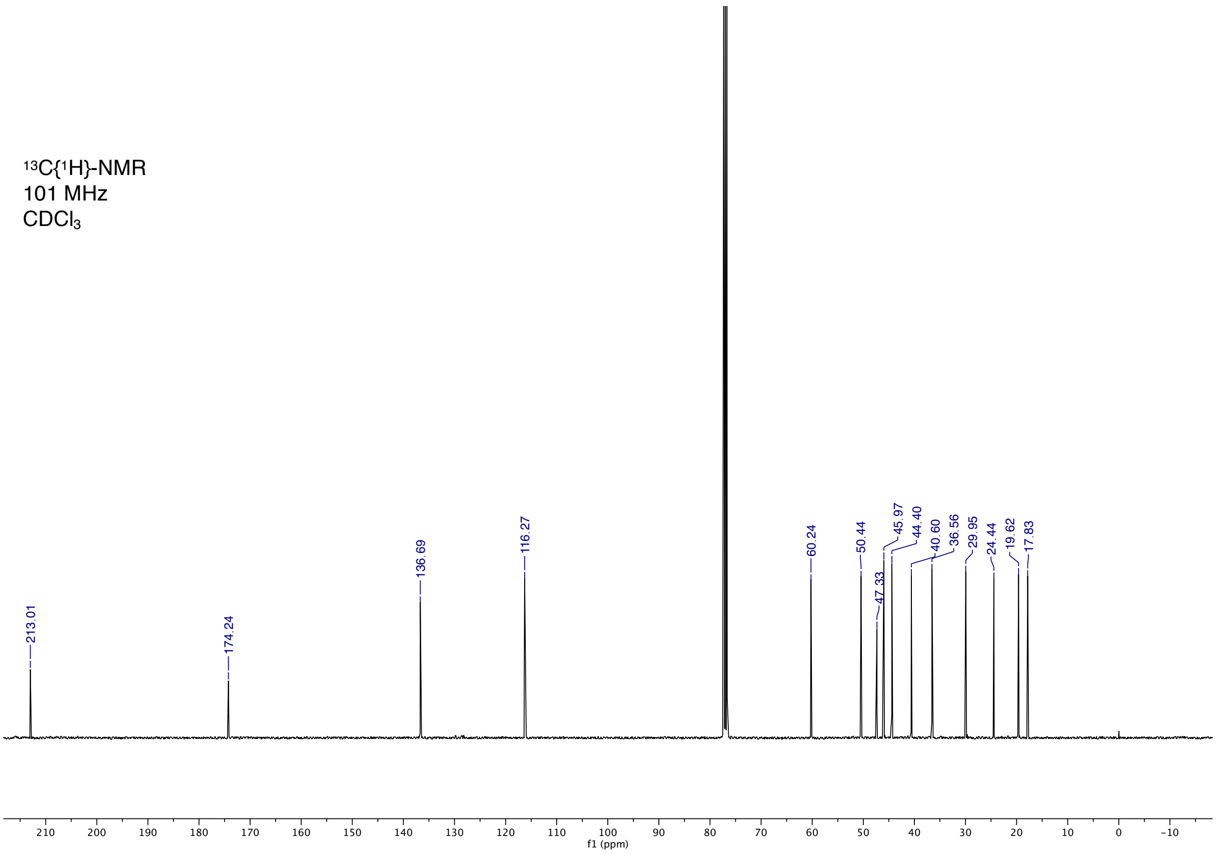

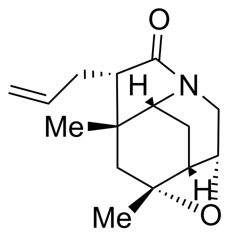

**10**

$^1$H-NMR
400 MHz
$CDCl_3$

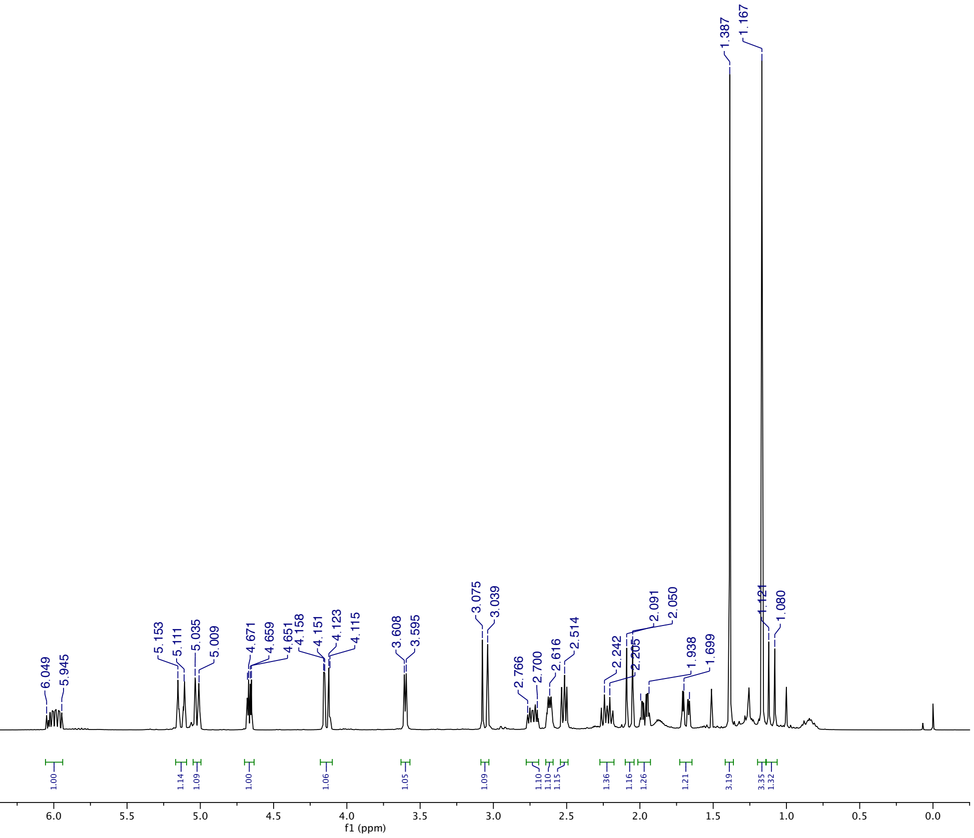


$^{13}C\{^1H\}$-NMR
101 MHz
$CDCl_3$

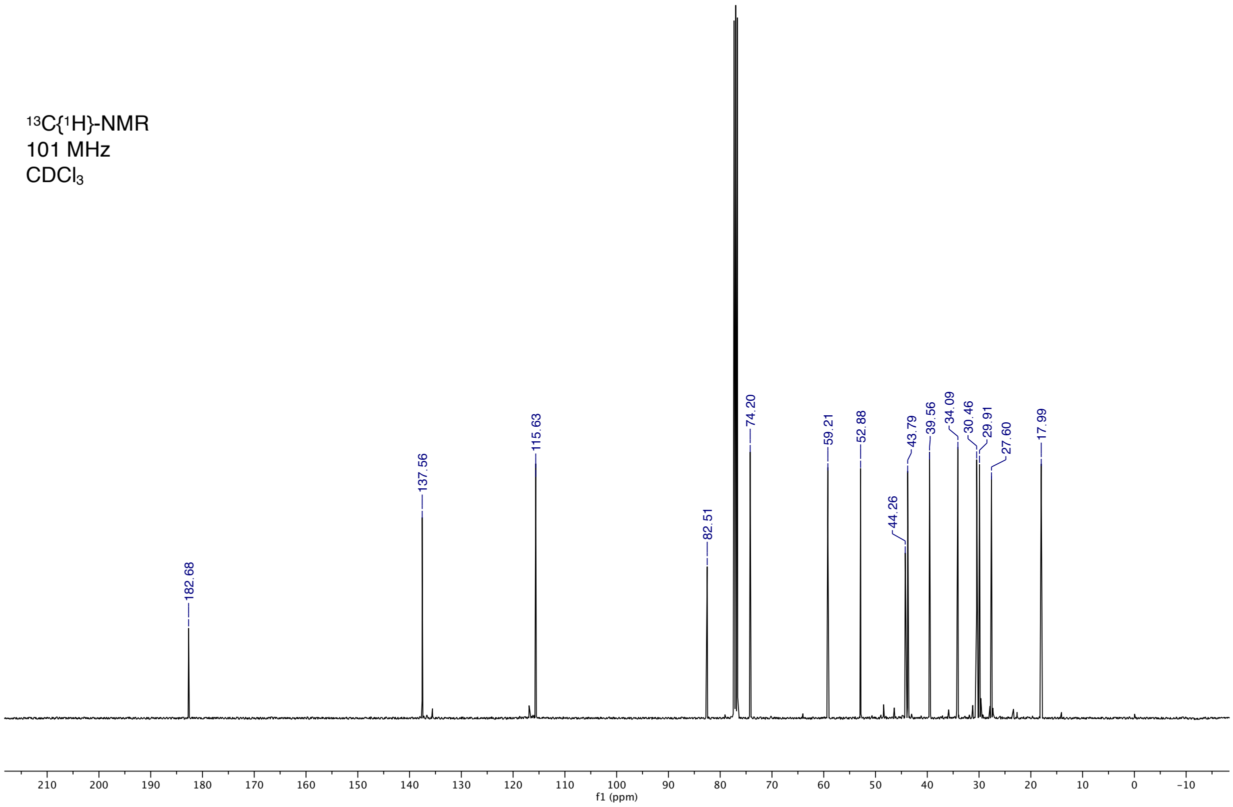

Supplement: Supplementary file 2 — jo2c01171_si_002.pdf [file jo2c01171_si_002.pdf]
